# A biophysically detailed computational model of urinary bladder small DRG neuron soma

**DOI:** 10.1371/journal.pcbi.1006293

**Published:** 2018-07-18

**Authors:** Darshan Mandge, Rohit Manchanda

**Affiliations:** Computational Neurophysiology Lab, Department of Biosciences and Bioengineering, Indian Institute of Technology Bombay, Mumbai, India 400076; SUNY Downstate MC, UNITED STATES

## Abstract

Bladder small DRG neurons, which are putative nociceptors pivotal to urinary bladder function, express more than a dozen different ionic membrane mechanisms: ion channels, pumps and exchangers. Small-conductance Ca^2+^-activated K^+^ (SK_Ca_) channels which were earlier thought to be gated solely by intracellular Ca^2+^ concentration ([Ca]_i_) have recently been shown to exhibit inward rectification with respect to membrane potential. The effect of SK_Ca_ inward rectification on the excitability of these neurons is unknown. Furthermore, studies on the role of K_Ca_ channels in repetitive firing and their contributions to different types of afterhyperpolarization (AHP) in these neurons are lacking. In order to study these phenomena, we first constructed and validated a biophysically detailed single compartment model of bladder small DRG neuron soma constrained by physiological data. The model includes twenty-two major known membrane mechanisms along with intracellular Ca^2+^ dynamics comprising Ca^2+^ diffusion, cytoplasmic buffering, and endoplasmic reticulum (ER) and mitochondrial mechanisms. Using modelling studies, we show that inward rectification of SK_Ca_ is an important parameter regulating neuronal repetitive firing and that its absence reduces action potential (AP) firing frequency. We also show that SK_Ca_ is more potent in reducing AP spiking than the large-conductance K_Ca_ channel (BK_Ca_) in these neurons. Moreover, BK_Ca_ was found to contribute to the fast AHP (fAHP) and SK_Ca_ to the medium-duration (mAHP) and slow AHP (sAHP). We also report that the slow inactivating A-type K^+^ channel (slow K_A_) current in these neurons is composed of 2 components: an initial fast inactivating (time constant ∼ 25-100 ms) and a slow inactivating (time constant ∼ 200-800 ms) current. We discuss the implications of our findings, and how our detailed model can help further our understanding of the role of C-fibre afferents in the physiology of urinary bladder as well as in certain disorders.

## Author summary

The small dorsal root ganglion (DRG) neurons of the bladder carry information related to pain from the bladder to the spinal cord. The level of electrical activity of these neurons is generally low but rises in abnormal conditions such as bladder inflammation and spinal cord injury. In order to investigate the reasons for this hyperactivity, we constructed a model of the bladder small DRG neuron soma including its membrane ion channels and intracellular Ca^2+^ dynamics. One of the channels, the small-conductance Ca^2+^-activated K^+^ (SK_Ca_) channel present in these neurons which was earlier thought to be activated solely by intracellular Ca^2+^ concentration was recently shown to exhibit inward rectification with respect to the neuron’s membrane potential. It was found that SK_Ca_ inward rectification can alter the repetitive firing of the bladder small DRG neurons. We also delineated the role SK_Ca_ and BK_Ca_ (large-conductance Ca^2+^-activated K^+^) channels to the complex afterhyperpolarization (AHP) of the neuron’s action potentials, shedding light on the mechanisms that govern repetitive firing in these sensory neurons. Our findings have implications for the normal biophysics and dysfunctional states of urinary bladder arising from changes in sensory axon function, and these are discussed.

## Introduction

The sensory component of the reflex pathway of the bladder is formed by the dorsal root ganglion (DRG) neurons. Afferent signals such as bladder pressure, volume, temperature, pH, presence of irritants and pain are transmitted to the spinal cord via these neurons. Urinary incontinence—the involuntary voiding of urine from the body—is a major group of disorders including overactive bladder in some of which sensory neuron pathophysiology has been implicated [1–6]. Biophysical changes at the axon terminals in the bladder and at the spinal cord as well as the alterations in the soma located in the DRG are major factors that may under certain conditions contribute to urinary bladder pathophysiology.

Two types of DRG neurons have been found to supply the human, rat and feline urinary bladder [3, 7]: the unmyelinated C-fibre neurons and myelinated A-*δ* fibre neurons. In rats, the C-fibre neurons have small soma diameter (∼24 *μ*m) and are therefore called small DRG neurons while the A-*δ* fibres have a somewhat larger soma diameter range (30-35 *μ*m) and are referred to as medium-sized or medium-diameter DRG neurons [3, 8]. The small DRG neurons are generally considered nociceptors i.e. they convey information related to pain. They may also take up the function of A-*δ* fibres (viz. sensing non-nociceptive stimuli; detrusor pressure, stretch, volume) in bladder dysfunction [3]. Significant changes in bladder small DRG neurons have been reported in various conditions such as bladder inflammation (interstitial cystitis), spinal cord injury (SCI), bladder overactivity, bladder outlet obstruction and hyperexcitability [3, 5, 9, 10].

The somata of these sensory neurons are located within the DRG and do not lie in the direct path of information propagation from the bladder to the spinal cord, the said path being constituted by the axon of the pseudounipolar DRG neuron. Historically, the DRG neuron somata were thought to play a purely metabolic role, for example in providing nutrition to the neurons, and synthesizing ion channels and various other proteins that are subsequently transported to axons and terminals. Recently, however, somata and the T-junction of the DRG neuron (the three-way junction where the axon bifurcates into two axon branches: the peripheral branch which innervates the sensory organ, and the central branch which conveys the electrical signals to the spinal cord) were found to be important players in filtering the signals from sensory branch of the axon [11–13], and in generating ectopic activity during nerve injury [14]. Moreover, the interaction within the ganglia between the DRG neuron somata and the satellite glial cells (SGCs) that sheath the DRG neuron soma has been implicated in the genesis of gastrointestinal pain [15]. Electrical stimulation of DRG, which is emerging as a new therapeutic strategy for pain alleviation [16] suggests a role for DRG neuron somata in regulating the electrical activity of the sensory neurons. For these reasons, the DRG neuron soma merits exploration in its own right.

In-vivo electrophysiological studies on individual somata are difficult to perform since DRG neuron somata are tightly packed with other somata in the dorsal root ganglia, as well as enveloped by the closely investing SGCs, blood vessels and extracellular matrix. A simple in-silico alternative to experiments which are otherwise much difficult to perform in-vivo is by the use of computational models and simulations. A comprehensive electrophysiological model of a DRG neuron soma would be a good starting point for such models. While a few models have been published for DRG neurons, e.g. for the GIT (Gastro-Intestinal Tract), others are for non-specific DRG neurons [13, 17–23]. None so far, however, is specific to bladder small DRG neurons. Furthermore, most existing models have been developed for medium- or other large-diameter DRG neurons and moreover, do not incorporate the effects of mitochondrial and endoplasmic reticulum calcium release and uptake mechanisms.

In order to address these questions, we therefore set out to build a biophysically detailed electrophysiological model of bladder small DRG neuron soma. Our aim was to build a model as tightly constrained as possible by available physiological data. Towards this end, we incorporated all the major membrane mechanisms known to exist in these neurons, including ion channels, pumps and Na^+^/Ca^2+^ exchanger. Although we adapted a number of models from existing reports, most of these models had not previously been validated, and we validated each such model individually. For instance, the simulated ionic currents were validated against the corresponding experimentally recorded currents, both in respect to their temporal dynamics and their current-voltage (I-V) relationship. We also incorporated intracellular Ca^2+^ dynamics including buffering, diffusion, endoplasmic reticulum (ER) and mitochondrial mechanisms. We then validated the action potentials and Ca^2+^ transients obtained from our physiologically constrained model against experimental data on bladder small DRG neuron action potentials and Ca^2+^ transients so as to be able to place confidence in our model’s robustness.

We proceed to employ our validated model in order to address certain questions important in the regulation of bladder small DRG neuron functions such as the following. (1) One of the Ca^2+^-activated K^+^ (K_Ca_) channels, the small-conductance K_Ca_ (SK_Ca_) channel which was earlier thought be activated by intracellular Ca^2+^ only, has been recently shown to exhibit inward rectification in addition to Ca^2+^ dependence [24–26]. Since the range of voltages over which rectification occurs (positive values of membrane potential, V_m_, [27, 28]), the rectifying SK_Ca_ channel would pass a smaller outward current, we hypothesized that incorporation of rectification should result in an increase of excitability of the bladder small DRG soma. Prior studies have also shown that inward rectification influences neuronal excitability markedly in other neurons [29]. Since the effects of SK_Ca_ current rectification on the excitability of bladder small DRG neurons or in any other excitable/non-excitable cell have not been studied yet, we first built a model of SK_Ca_ channel incorporating rectification (see Methods), included this channel in our soma model, and finally employed the soma model to address our hypothesis. (2) SK_Ca_ along with BK_Ca_ (large-conductance K_Ca_) channels can govern the excitability of the neurons by contributing to the afterhyperpolarization (AHP) of the action potentials (APs). The origins and function of the AHPs observed in bladder DRG neuron AHPs is not clearly delineated [30]. The genesis of the AHPs has been proposed to be different in different neurons. For instance, in vagal sensory neurons, BK_Ca_ channels are thought to underlie medium duration AHP (mAHP) [31] while in several central nervous system neurons, the mAHP is controlled by SK_Ca_ channels [32]. In the face of these conflicting proposals, and in view of the importance of AHPs in governing neuronal excitation (especially frequency), we thought to resolve the role of K_Ca_ channels underlying the AHPs in bladder sensory neurons. (3) The A-type K^+^ (K_A_) channels found in the bladder DRG neurons are important regulators of excitability of neurons in conditions such as bladder cystitis and spinal cord injury [3]. K_A_ channels have faster activation time constants than KDR channels, and can alter the depolarizing phase of an AP. The K_A_ channels expressed in bladder DRG neurons are of 2 types: the rapidly-inactivating K_A_ (fast K_A_) which are present in medium-diameter neurons and a slow-inactivating K_A_ (slow K_A_) found in small DRG neurons [3, 7, 33–35]. Both these currents have a transient rising phase and a fast or slow inactivating phase when recorded under rectangular voltage clamps. Even though several studies have been carried out to decipher the molecular components giving rise to this K_A_ current [34–36], the question is as yet unresolved. (4) Another unresolved issue pertains to the relative importance of BK_Ca_ and SK_Ca_ channels in regulating firing rate in these neurons during repetitive firing. We used our model to shed light on both these unsettled biological questions, i.e. (3) and (4).

Using our validated model, we show that incorporation of rectifying as opposed to non-rectifying SK_Ca_ channels increases the excitability of the bladder small DRG neurons. It was also found from conductance-based studies that SK_Ca_ channels are more potent than BK_Ca_ channels in controlling the firing rate of these neurons. By exercising our detailed model, we are able to propose that the fast afterhyperpolarization (fAHP) in bladder small DRG neurons may result primarily from BK_Ca_ channels while SK_Ca_ may contribute primarily to the mAHP and the slow AHP (sAHP). Furthermore, we found the SK_Ca_ channel to be a more potent regulator of repetitive firing than the BK_Ca_ channel. By the use of channel modelling and optimization, we found that the slow inactivating A-type K^+^ (slow K_A_) current in bladder small DRG neurons is composed of two inactivating components: a relatively fast and a relatively slower component. This could arise from contributions of two different molecular constituents of the channel or because of interactions between two different inactivation states of K_v_1.4. In sum, having developed and validated a detailed and physiologically constrained model of the bladder small DRG neuron, we employed in order to illuminate questions of the type that computational models are ideally suited for: (1) hypothesis testing, in regard to our prediction that the inward rectifying SK_Ca_ channel would render the neuron more excitable; (2) taking into account previous conflicting contentions and resolving them in regard to the relative contributions of K_Ca_ channels to the AHP; (3) shedding light on an open biological question, as regards the probable subunit composition of the slow K_A_ channel in these neurons and assessing the relative contributions of SK_Ca_ and BK_Ca_ channels in governing repetitive firing. We discuss the implications of our findings in the framework of bladder small DRG neuron functioning and of bladder physiology as modulated by these afferents. Initial studies related to this work were published in Mandge et al. [37, 38] and Aruljothi et al. [39].

## Methods

A physiologically constrained computational model of bladder small DRG neuron soma was created using the data on the morphology, active and passive membrane mechanisms, and Ca^2+^ dynamics from the literature. The NEURON simulation environment v7.3 [40] was used for modelling and simulations of the DRG neuron. An Intel i5 processor based desktop computer with 4 cores working at 3.10 GHz was used for running the simulations. All the experimental data were obtained from published literature and digitized using the webPlotDigitizer tool (https://automeris.io/WebPlotDigitizer). Data were analysed and plotted using OriginPro (OriginLab Corp.) and MATLAB (MathWorks, Inc).

The soma was modelled as a sphere of diameter 24 *μ*m. The membrane potential was calculated using the following equation:

$$\frac{dV_{m}}{dt}=\frac{1}{C_{m}}(I_{Stim}-I_{membrane})$$ (1)

where *I*_*Stim*_ is the stimulus current, *I*_*membrane*_ is the ionic current through cell membrane mechanisms: ion channels, pumps and exchanger and is carried by Na^+^, K^+^, Ca^2+^ and Cl^−^ ions.

The model description is divided into following subheadings: passive properties, soma membrane mechanisms and Ca^2+^ dynamics.

### Passive properties

Passive parameters such as total membrane capacitance (C_*m*_) and specific membrane resistance (R_m_) along with other model specifications are given in Table 1. Input resistance (R_in_) of the model was found to be 316.05 MΩ. It was found by using a long duration hyperpolarizing current clamp of −0.01 nA and calculated by dividing the change observed in membrane potential, V_m_ (between the resting membrane potential, RMP and the steady state V_m_) and the current amplitude. The observed R_in_ from the model is in range of values reported in literature for dissociated bladder small DRG neurons i.e. 175 MΩ-581 MΩ [7, 41] and is also close to the value reported for intact bladder DRG neurons (332 MΩ) [42].

**Table 1:** Model specifications and ionic concentrations.

Property	Value	Reference
Resting Membrane Potential, RMP	−53.5 mV	[10]
Soma Diameter	24 *μm*	[10]
Total Membrane Capacitance	28 pF	[35]
Specific Membrane Resistance, R_m_	10000 Ω-cm^2^	[43]
Axial Resistance, R_a_	100 Ω-cm	[13]
Intracellular K^+^ Concentration, [K]_i_	140 mM	[7]
Extracellular K^+^ Concentration, [K]_o_	5 mM	[7]
Nernst Potential of K^+^, E_K_	−84.7 mV	Calculated
Intracellular Na^+^ Concentration, [Na]_i_	10 mM	[33]
Extracellular Na^+^ Concentration, [Na]_o_	150 mM	[7]
Nernst Potential of Na^+^, E_Na_	68.9 mV	Calculated
Resting Intracellular Ca^2+^ Concentration, [Ca]_i_	1.36*10^−4^ mM	[44]
Extracellular Ca^2+^ Concentration, [Ca]_o_	2 mM	[45]
Initial Nernst Potential of Ca^2+^, E_Ca_	122 mV	Calculated
Intracellular Cl^-^ Concentration, [Cl]_i_	40 mM	[46]
Extracellular Cl^-^ Concentration, [Cl]_o_	145 mM	Calculated
Nernst Potential of Cl^-^, E_Cl_	−32.7 mV	[46]
Temperature, T	22°C	[7]

A passive channel was added to the model to incorporate the currents through voltage-independent channels reported in small DRG neurons such as the K_2P_ channels: TREK1, TREK2, TRAAK [47]. The channel current was calculated as follows:

$$I_{pas}=g_{pas}(V_{m}-E_{pas})$$ (2)

Where *g*_*pas*_ = 1/R_m_ = 1/(10000 Ω-cm^2^) = 1*10^−4^ S/cm^2^ and *E*_*pas*_ was set to −41.583 mV to have a stable RMP of −53.5 mV.

### Soma membrane mechanisms

Twenty-two ionic membrane mechanisms including ion channels, pumps and exchangers were built/ adapted, validated for their parameters such as time constants and steady state of (in)activation as well as for voltage clamp currents and I-V relationships, and were then incorporated into the bladder small DRG neuron soma model (see S1 Fig). All the known membrane ion channels reported for bladder small DRG neurons along with membrane mechanisms described essential in other small DRG neuron functioning were added. Table 2 shows the plasma membrane mechanisms added to the model along with their data source and animal species from which the data was obtained.

**Table 2:** Plasma membrane ionic mechanisms in the model and their data source.

Mechanisms	Species and Neuron Type	Reference
Passive Channels	Bladder DRG Neurons	[47, 48]
***Na^+^ Channels***		
Tetrodotoxin-Sensitive (TTX-S) Channel	Rat, Bladder small DRG neurons	[7]
	Rat, Small DRG neurons	[20]
	Transfected HEK Cells	[21]
*Tetrodotoxin-Resistant (TTX-R) Channels*		
Na_v_1.8 Channel	Rat, Bladder small DRG neurons	[7, 9, 49]
	Rat, Small DRG Neurons	[50]
Na_v_1.9 Channel	Rat, Bladder DRG neurons	[51]
	Mouse Small DRG neurons	[20]
***K^+^ Channels***		
A-type K^+^ (K_A_) Channel	Rat, Bladder small DRG neurons	[10, 33]
Delayed Rectifier (KDR) Channel	Rat, Bladder DRG neurons	[33]
	Transfected HEK Cells	[21]
*Ca^2+^-Activated K^+^ Channels (K_Ca_)*		
Large-Conductance Ca^2+^-Activated K^+^ Channel (BK_Ca_)	Rat, L6-S1 Small DRG neurons	[52]
	Rat, Small DRG neurons	[53]
	Rat, Cutaneous small DRG neurons	[54]
Small-Conductance Ca^2+^-Activated K^+^ Channel (SK_Ca_): hSK3	Rat, Bladder small DRG neurons	[30]
	Transfected HEK Cells	[27, 30, 55]
*Other K^+^ Channels*		
KCNQ/M Channel	Rat, Bladder Small DRG neurons	[42]
	Rat, Small DRG neurons	[56–58]
Na^+^-activated K^+^ (K_Na_) Channel	Rat, Small DRG neurons	[23, 59]
**Ca^2+^ Channels**		
Voltage-gated Ca^2+^ (Ca_v_) Channels		
L-type Ca^2+^ Channel	Chick, Thoracic-Lumbar DRG neurons	[60]
	Uterine Smooth Muscle cells	[61]
N-type Ca^2+^ Channel	Chick, Thoracic-Lumbar DRG neurons	[60, 62, 63]
	Uterine Smooth Muscle cells	[61]
P/Q-type Ca^2+^ Channel	Mouse, Lumbar (L3-L6) DRG neurons	[64]
R-type Ca^2+^ Channel	Mouse, DRG neurons	[65, 66]
	Hippocampal Mossy Fibre Boutons	[67]
T-type Ca^2+^ Channel	Chick, Thoracic-Lumbar DRG neurons	[60]
	Straital Medium Spiny neurons	[29]
***Other Channels/Mechanisms***		
Hyperpolarization-Activated Cyclic Nucleotide-Gated Channel	Rat, Bladder small DRG neurons	[8, 68]
	Rat, Small DRG neurons	[69]
Store-Operated Ca^2+^ Channel (SOCC)	Rat, DRG neurons	[70]
	Jurkat T cells	[71]
Ca^2+^-activated Cl^−^ Channel (CaCC)	Rat, Small DRG Neurons	[72, 73]
	Transfected HEK-293 cells	[74]
Transient Receptor Potential Cation Channel Subfamily M Member 8	Rat, Bladder small DRG neurons	[3, 75]
	DRG neurons	[76]
Na^+^/K^+^-ATPase Pump or Na^+^/K^+^ Pump	Rat, L4-L6 DRG Neurons	[23, 77]
Na^+^/Ca^2+^ Exchanger (NCX)	Rat, Cutaneous Small DRG neurons	[78]
	Cardiac Atrial cells	[79]
Plasma Membrane Ca^2+^-ATPase (PMCA) Pump	-	[48]

General modelling formalisms used for the mechanisms are explained in S1 Text. Validation of individual model mechanisms are explained ahead and in S2 Text. The ionic concentrations, RMP, membrane capacitance and maximum density (or conductance or permeability) used for validating individual mechanisms were fixed as per the experimental protocol reported for that mechanism and may be different from parameters used for the complete model (Table 1). These and other experimental parameters used for validating individual mechanisms are provided along with their respective descriptions in the text and figures. The maximum densities were tuned for the complete soma model to give experimentally reported action potential (AP) properties such AP amplitude, AP overshoot and AP duration, and Ca^2+^ transients (see Results). Tuning was done in accordance with the densities reported in experiments, for instance, conductance of Na_v_1.8 channels was higher than Na_v_1.9 and TTX-sensitive Na^+^ channels as reported in [7] and [51]. S1 Table has the membrane mechanism densities, maximum conductances and maximum permeabilities used for the complete model.

Standard error of the regression (S) also called the root mean squared error (RMSE) was used to test the goodness-of-fit for channel parameters, channel currents, action potentials and intracellular and mitochondrial Ca^2+^ concentrations as R^2^ is not good estimate of the goodness of fits for such nonlinear models [80]. S gives the standard deviation of residuals between the model fit curve and the experimental points. The lower the value of S, the better is the fit [81]. The value of threshold below which the fit is good was chosen as 5% of the difference between the maximum and minimum values taken by that parameter e.g. for the steady state activation/inactivation parameters (*m*_∞_ and *h*_∞_), the value is 0.05 (5% of 1). The threshold values for parameters of membrane mechanisms, APs and Ca^2+^ concentrations are given along with their respective figures in captions. S value is given by:

$$S=\sqrt{\frac{\Sigma {\left(Y_{expt}-Y_{fit}\right)}^{2}}{v}}$$ (3)

where *Y*_*expt*_ is the experimental value, *Y*_*fit*_ is the corresponding value from the curve fit, *v* is the residual degrees of freedom which is the difference in number of data points, *n* and the number of parameters used to fit the curve. For instance, *m*_∞_ in Boltzmann equation has 2 fit parameters: half-activation potential and slope factor and hence, *v* = *n* − 2. For AP and Ca^2+^ concentrations goodness-of-fits, *v* was taken as *n* − 1. S value was not calculated for parameters with *v* < 4.

The remaining part of the section describes the modelling and validation of individual mechanisms and Ca^2+^ dynamics of the bladder small DRG neuron model.

#### Na^+^ channels

Bladder small DRG neurons Na^+^ channels have been classified on the basis of tetrodotoxin (TTX) sensitivity into 2 categories: Tetrodotoxin-Sensitive (TTX-S) Na^+^ channels and Tetrodotoxin-Resistant (TTX-R) Na^+^ channels. Both types are expressed in bladder small- and medium-diameter DRG neurons but the contribution of TTX-R currents to total Na^+^ currents is higher in small neurons (> 85%) while in the medium-diameter neurons, 60-100% of total Na^+^ currents can be carried by TTX-S channels [3, 7]. The diversity in the Na^+^ channels arise from their structure comprising of a pore forming *α* subunit which has the sites of inactivation and drug binding, and *β* subunits which determine kinetic and voltage dependent properties of the channels [82]. Na^+^ current is the main depolarizing current in the bladder afferent neurons [7], and thus, Na^+^ channels form the target for various bladder therapeutic strategies. Na^+^ channels found in bladder DRG neurons have been described below.

**Fig 1:**
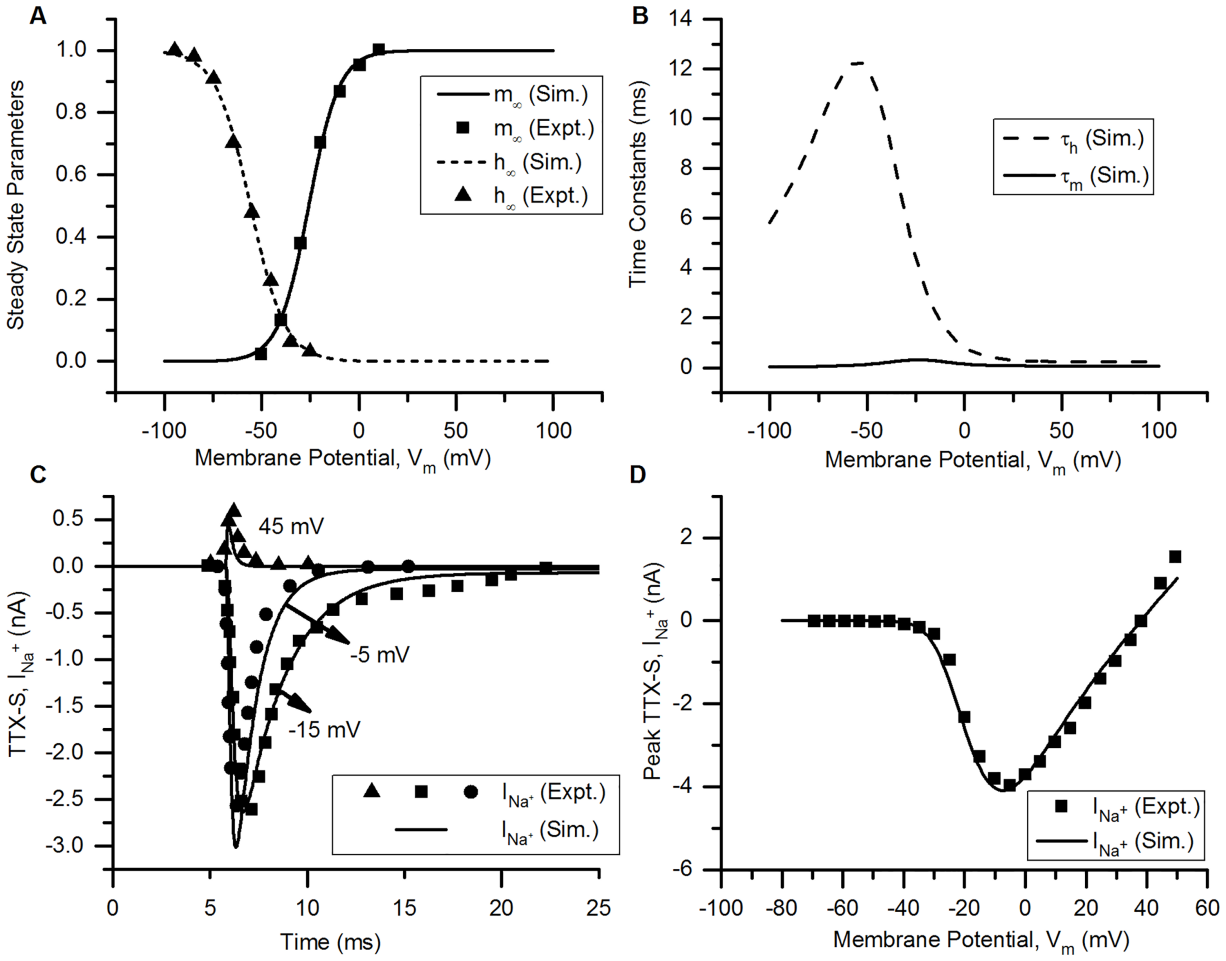
TTX-S Na^+^ channel. (A) Voltage dependence of steady state activation (*m*_∞_) and inactivation (*h*_∞_) of the channel. Squares (=*m*_∞_) and triangles (=*h*_∞_) represent the experimental data (Expt.) for bladder medium-diameter DRG neuron [7]. Solid (*m*_∞_) and dashed (*h*_∞_) lines represents simulation results (Sim.). (B) The voltage dependence of time constants of activation (*τ*_*m*_, solid line) and inactivation (*τ*_*h*_, dashed line). (C) Rectangular voltage clamp currents, TTX-S I_Na_ for different clamp levels from experiments [7] (symbols, squares: −15 mV, circles: −5 mV and triangles: 45 mV) and simulation (corresponding solid lines). The holding potential was kept at −70 mV and the test potentials of 25 ms were applied to −15, −5 and 45 mV. (D) Peak current-voltage (I-V) relationship obtained using experiments (squares, [7]) and simulations (solid line). Protocol: Rectangular voltage clamp from −80 to 40 mV in steps of 5 mV from a holding potential of −60 mV. The peak currents at each test potentials are plotted. Other parameters: $\bar{g}=0.0076\;\text{S}/\text{cm}{}^{2}$, E_Na_ = 38 mV, RMP = −56.5 mV, soma diameter = 32.4 *μ*m [7] and total capacitance = 31.7 pF [8]. The S values for model fits and their 5% threshold values (given in brackets) are: *m*_∞_ = 0.017 (0.05), *h*_∞_ = 0.021 (0.05), for voltage clamp currents: at −45 mV = 0.183 nA (0.033 nA), at −15 mV = 0.189 nA (0.137 nA) & at −5 mV = 0.334 nA (0.129 nA), and I-V curve = 0.246 nA (0.276 nA).

**Fig 2:**
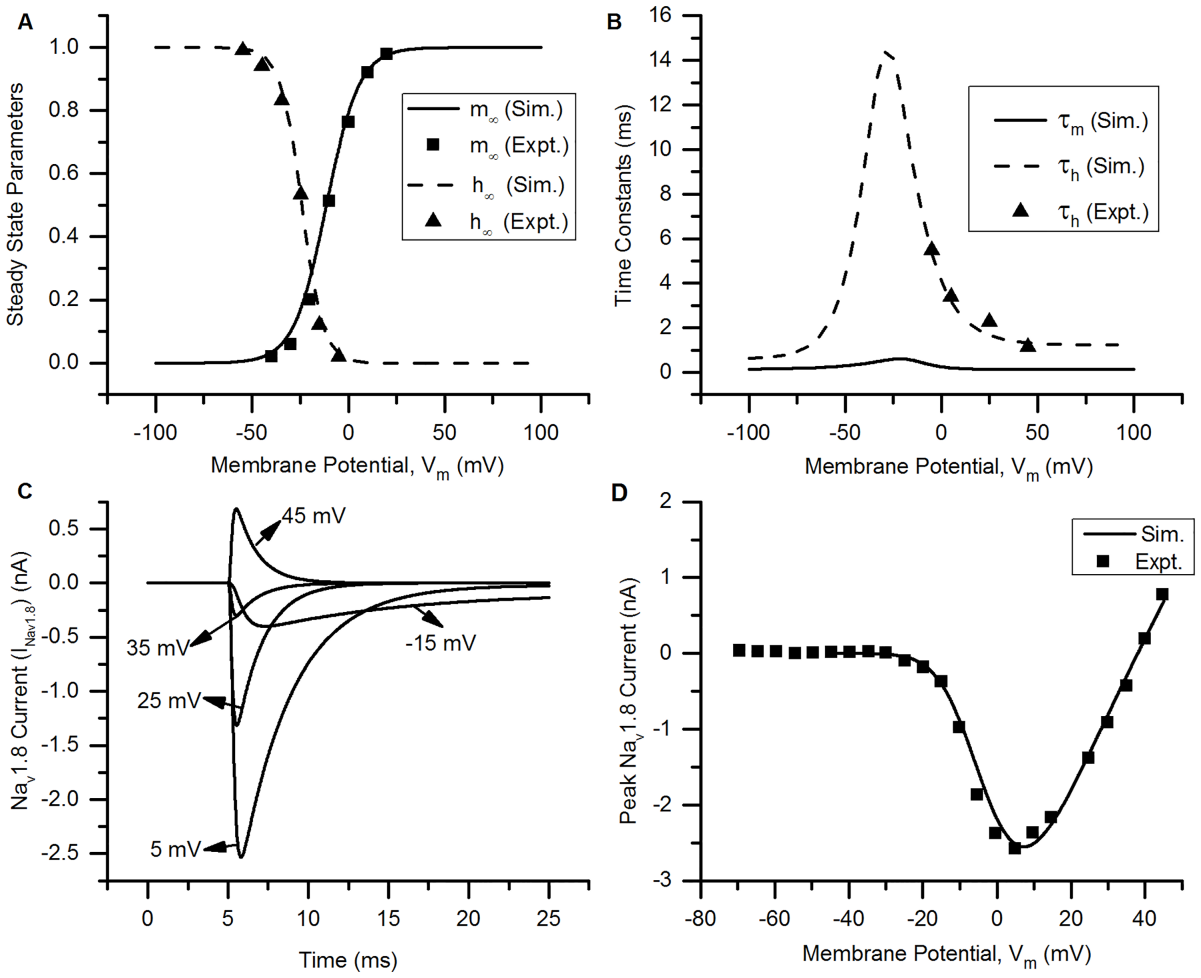
Na_v_1.8 channel. (A) Voltage dependence of steady state activation (*m*_∞_) and inactivation (*h*_∞_) of the channel. Squares (=*m*_∞_) and triangles (=*h*_∞_) are the experimental data (Expt.) for bladder small DRG neuron [7]. Solid (*m*_∞_) and dashed (*h*_∞_) lines represents simulation results (Sim.). (B) The voltage dependence of time constants of activation (*τ*_*m*_, solid line) and inactivation (*τ*_*h*_, dashed line). Triangles (=*τ*_*h*_) are the experimental data (Expt.). (C) *Na*_*v*_1.8 currents (I_*Na*_*v*_1.8_) from simulation (solid lines) using rectangular voltage clamp protocol. The holding potential was kept at −70 mV for 5 ms and the test potentials of 50 ms were applied at −15, 5, 25, 35 and 45 mV. (D) Peak I-V relationships obtained from experiments (squares, [7]) and simulations (solid line). Protocol: Rectangular voltage clamp from −80 to 45 mV in steps of 10 mV from a holding potential of −60 mV. The peak currents is plotted for each test potential. Other parameters: $\bar{g}=0.0086\;\text{S}/\text{cm}{}^{2}$, E_Na_ = 38 mV, RMP = −53.5 mV, total capacitance = 28 pF and soma diameter = 24 *μ*m [7]. The S values for model fits and their 5% threshold values (given in brackets) are: *m*_∞_ = 0.042 (0.05), *h*_∞_ = 0.033 (0.05), and I-V curve = 0.115 nA (0.203 nA).

*TTX-S Na^+^ channels*. TTX-S Na^+^ channel expression is higher in medium-diameter as compared to small DRG neurons of bladder, and form the major inward currents during an action potentials (APs). Low micromolar concentrations of TTX (≤ 1 *μ*M) can prevent the generation of APs in medium-diameter bladder DRG neurons but not in small DRG neurons [7]. Smaller depolarizations from RMP are sufficient to activate TTX-S channels as compared to voltage-gated TTX-R Na_v_1.8 channels. This can be seen by comparing the steady state activation (m_∞_) curves for these channels (Figs 1A and 2A).

The data for modelling the TTX-S channels of bladder small DRG neurons were obtained by fitting the data from [7, 20, 21]. The equations used for modelling are:

$$m_{\infty }=\frac{1}{1+\text{exp}\left(\frac{-25.8-V_{m}}{7.8}\right)}\;h_{\infty }=\frac{1}{1+\text{exp}\left(\frac{V_{m}+55.8}{8.9}\right)}$$ (4)

$$\alpha_{m}=\frac{15.5}{1+\text{exp}(\frac{V_{m}-5}{-12.08})}\;\beta_{m}=\frac{35.2}{1+\text{exp}(\frac{V_{m}+72.7}{16.7})}$$ (5)

$$\alpha_{h}=0.24\;\text{exp}(\frac{-(V_{m}+115)}{46.33})\;\beta_{h}=4.32\;\text{exp}(\frac{\left(V_{m}-11.8\right)}{-12})$$ (6)

$$\tau_{m}=\frac{1}{\alpha_{m}+\beta_{m}}\;\tau_{h}=\frac{1}{\alpha_{h}+\beta_{h}}\;\frac{dm}{dt}=\frac{m_{\infty }-m}{\tau_{m}}\;\frac{dh}{dt}=\frac{h_{\infty }-h}{\tau_{h}}$$ (7)

$$I_{Na_{TTX-S}}=\bar{g}m^{3}h\left(V_{m}-E_{Na}\right)\;\bar{g}=0.0076\;\text{S/cm}{}^{2}\;E_{Na}=38\;\text{mV}$$ (8)

*TTX-R channels*. Two types of voltage-gated (Na_v_) TTX-R channels have been reported in bladder small DRG neurons: Na_v_1.8 which produces slowly-inactivating and Na_v_1.9 channels generating persistent currents [51]. The expression of Na_v_1.8 is substantially higher than that of Na_v_1.9 channels [51]. Both the channels exhibit different activation potentials [20, 51] and play an important role in bladder nociception [3, 9, 49].

*Na_v_1.8 − slowly-inactivating TTX-R Na^+^ channels*. Na_v_1.8 channels play an important role in bladder hyperactivity and chemical irritation. Blocking the expression of these channels reduces the total Na^+^ current and inhibits the increased contractility of the bladder hyperreflexia [3]. They form the largest component of the inward current (> 80%) generated during an action potential in bladder small DRG neurons [7] and contribute to excitability in normal and pathophysiological conditions [7, 9, 51].

The data for modelling the channel was derived from [7, 9, 49, 50] for bladder small DRG neurons. Fig 2 shows the validation of the modelled channel. The equations used for modelling are:

$$m_{\infty }=\frac{1}{1+\text{exp}(\frac{-11.4-V_{m}}{8.5})}\;h_{\infty }=\frac{1}{1+\text{exp}(\frac{V_{m}+24.2}{5.6})}$$ (9)

$$\alpha_{m}=7.21-\frac{7.21}{1+\text{exp}(\frac{V_{m}-0.063}{7.86})}\;\beta_{m}=\frac{7.4}{1+\text{exp}(\frac{V_{m}+53.06}{19.34})}$$ (10)

$$\alpha_{h}=0.003+\frac{1.63}{1+\text{exp}(\frac{V_{m}+68.5}{10.01})}\;\beta_{h}=0.81-\frac{0.81}{1+\text{exp}(\frac{V_{m}-11.44}{13.12})}$$ (11)

$$\tau_{m}=\frac{1}{\alpha_{m}+\beta_{m}}\;\tau_{h}=\frac{1}{\alpha_{h}+\beta_{h}}\;\frac{dm}{dt}=\frac{m_{\infty }-m}{\tau_{m}}\;\frac{dh}{dt}=\frac{h_{\infty }-h}{\tau_{h}}$$ (12)

$$I_{Na_{v}1.8}=\bar{g}m^{3}h\left(V_{m}-E_{Na}\right)\;\bar{g}=0.0086\;\text{S/cm}{}^{2}\;E_{Na}=38mV$$ (13)

Na_v_1.9 channel was adapted from [20] and is described in S2 Text.

#### K^+^ currents

In bladder small afferents neurons, among the many types K^+^ channels expressed, transient A-type (K_A_) channel (slowly-inactivating), delayed-rectifier (KDR) and KCNQ/M channels are the predominant ones [7, 10, 33, 42]. Presence of Ca^2+^-activated K^+^ (K_Ca_) channels: large-conductance K_Ca_ (BK_Ca_) and small-conductance K_Ca_ (SK_Ca_) in bladder small DRG neurons have suggested by some studies [30, 52]. Na^+^-activated K^+^ (K_Na_) channels form the leakage currents contributing to RMP in small DRG neurons [59].

*A-type K^+^ (K_A_) channels*.

K_A_ channels have faster activation time constants than KDR channels, and can alter the depolarising phase of an AP. The K_A_ channels expressed in bladder DRG neurons are of 2 types: the fast-inactivating K_A_ (fast K_A_) which are present in medium-diameter neurons and a slow-inactivating K_A_ (slow K_A_) found in small DRG neurons [3, 7, 33–35]. Both these currents have a transient rising phase and a fast or slow inactivating phase when recorded under step voltage clamps. The slow K_A_ is different from the fast K_A_ in terms of inactivation time constants and steady state parameters (*m*_∞_ and *h*_∞_). *h*_∞_ of fast K_A_ is shifted 20 mV towards hyperpolarizing voltages when compared to slow K_A_ currents [3]. Because of the availability of slow K_A_ currents near the RMP in bladder small DRG neurons, the threshold for evoking an action potential is higher as compared to medium-diameter neurons in which the fast K_A_ currents are completely inactivated at RMP [3]. The slow K_A_ channels start activating around −120 mV. The underlying molecular components of slow K_A_ currents in bladder small DRG neurons are not completely known. Some studies have suggested that K_v_1.1, K_v_1.2 and K_v_1.4 could contribute to these currents [34–36].

**Fig 3:**
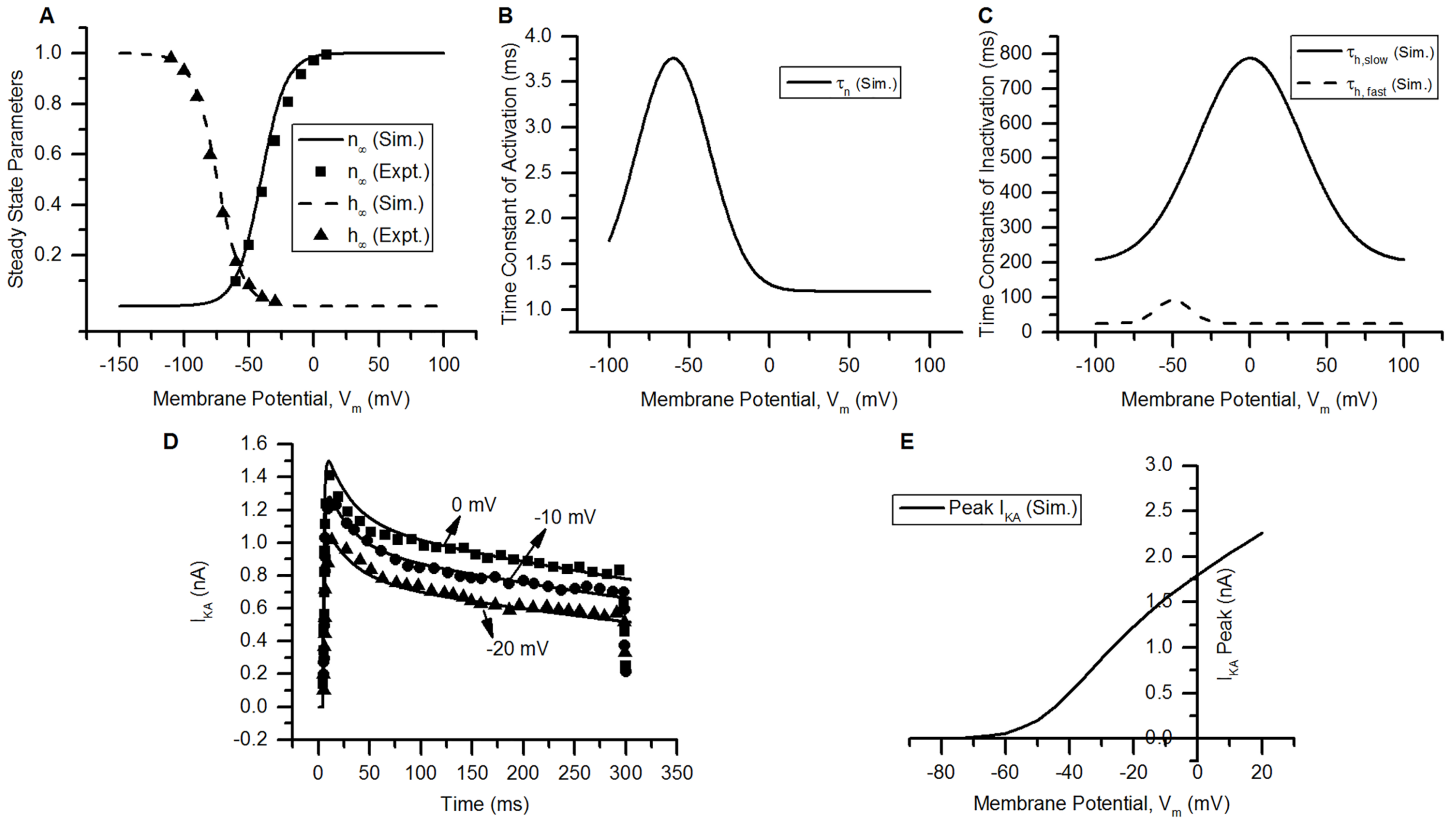
K_A_ channel. (A) Voltage dependence of steady state activation (*n*_∞_, solid line) and inactivation (*h*_∞_, dashed line) of the modelled channel. Squares (*n*_∞_) and triangles (*h*_∞_) represent the experimental data for bladder small DRG neurons from [7]. (B) The activation time constant(*τ*_*n*_) of the modelled channel. (C) The inactivation time constants of the channel: *τ*_*h*,*slow*_ (= slow time constant, solid line) and *τ*_*h*,*fast*_ (= fast time constant, dashed line). (D) The currents (*I*_*KA*_) generated by the model (solid lines) and the currents reported in experiments (symbols, [33]) using rectangular voltage clamps. The holding potential was kept at −120 mV for 5 ms and test potentials of −20, −10 and 0 mV were applied for 300 ms. Experimental data for currents recorded is shown by triangles (= −20 mV), circles (= −10 mV) and squares (= 0 mV) along corresponding simulated currents (solid lines). (E) Peak I-V relationship from the model generated by recording the peak current at each test potential. Protocol: Rectangular voltage clamp steps were applied from −80 to 20 mV for 300 ms from a holding potential of −80 mV. Other model parameters: $\bar{g}=0.00108\;\text{S}/\text{cm}{}^{2}$, E_K_ = −84.7 mV, RMP = −53.5 mV, membrane capacitance = 28 pF, soma diameter = 24 *μ*m. The S values for model fits and their 5% threshold values (given in brackets) are: *n*_∞_ = 0.008 (0.05), *h*_∞_ = 0.003 (0.05), for voltage clamp currents at: 0 mV = 0.157 nA (0.067 nA), −10 mV = 0.161 nA (0.059 nA) and −20 mV = 0.081 nA (0.045 nA).

On close inspection of slow K_A_ currents found in bladder small DRG neurons reported in [33, 34], it was found that they inactivate in 2 phases, a fast and a slow phase. This was also supported by fitting biexponential decay equations to falling phase of the slow K_A_ currents. Hence, slow K_A_ currents were modelled as having three parameters—an activation parameter (*n*), a fast inactivation parameter (*h*_*fast*_) and a slow inactivation parameter (*h*_*slow*_). The inactivation parameters were assumed to have a same steady state of inactivation (*h*_∞_) but different time constants. This method has been reported in [83]. The *h*_*fast*_ parameter whose time constant are between 25 and 100 ms, give rise to the initial fast decay of the currents seen in the voltage clamps, and the *h*_*slow*_ parameter with time constants between 200-800 ms results in the slow decaying phase of K_A_ currents (See Fig 3C). The contribution of the 2 inactivation parameters was scaled (0.3 for *h*_*fast*_ and 0.7 for *h*_*slow*_) such that their maximum sum is unity. The slow K_A_ current equation is given by:

$$n_{\infty }=\frac{1}{1+\text{exp}(\frac{-40.8-V_{m}}{9.5})}\;h_{\infty }=\frac{1}{1+\text{exp}(\frac{V_{m}+74.2}{9.6})}$$ (14)

$$\tau_{n}=1.2+2.56\text{exp}(-2(\frac{\left(V_{m}+60\right)}{45.76}{)}^{2})$$ (15)

$$\tau_{h,fast}=25.46+67.41\text{exp}(-2(\frac{V_{m}+50}{21.95}{)}^{2})\;\tau_{h,slow}=200+587.4\text{exp}(-(\frac{V_{m}}{47.77}{)}^{2})$$ (16)

$$\frac{dn}{dt}=\frac{n_{\infty }-n}{\tau_{n}}\;\frac{dh_{fast}}{dt}=\frac{h_{\infty }-h_{fast}}{\tau_{h,fast}}\;\frac{dh_{slow}}{dt}=\frac{h_{\infty }-h_{slow}}{\tau_{h,slow}}\;\bar{g}=0.00108\;\text{S}/\text{cm}{}^{2}$$ (17)

$$I_{KA}=\bar{g}n\left(0.3h_{fast}+0.7h_{slow}\right)\left(V_{m}-E_{K}\right)\;E_{K}=-84.7\;\text{mV}$$ (18)

The two inactivation profiles could arise as a result of contribution of 2 K_v_ subunits probably K_v_1.4 and K_v_1.2 or as results of interaction of 2 inactivation states exhibited by K_v_1.4 subunit (see Discussion).

**Fig 4:**
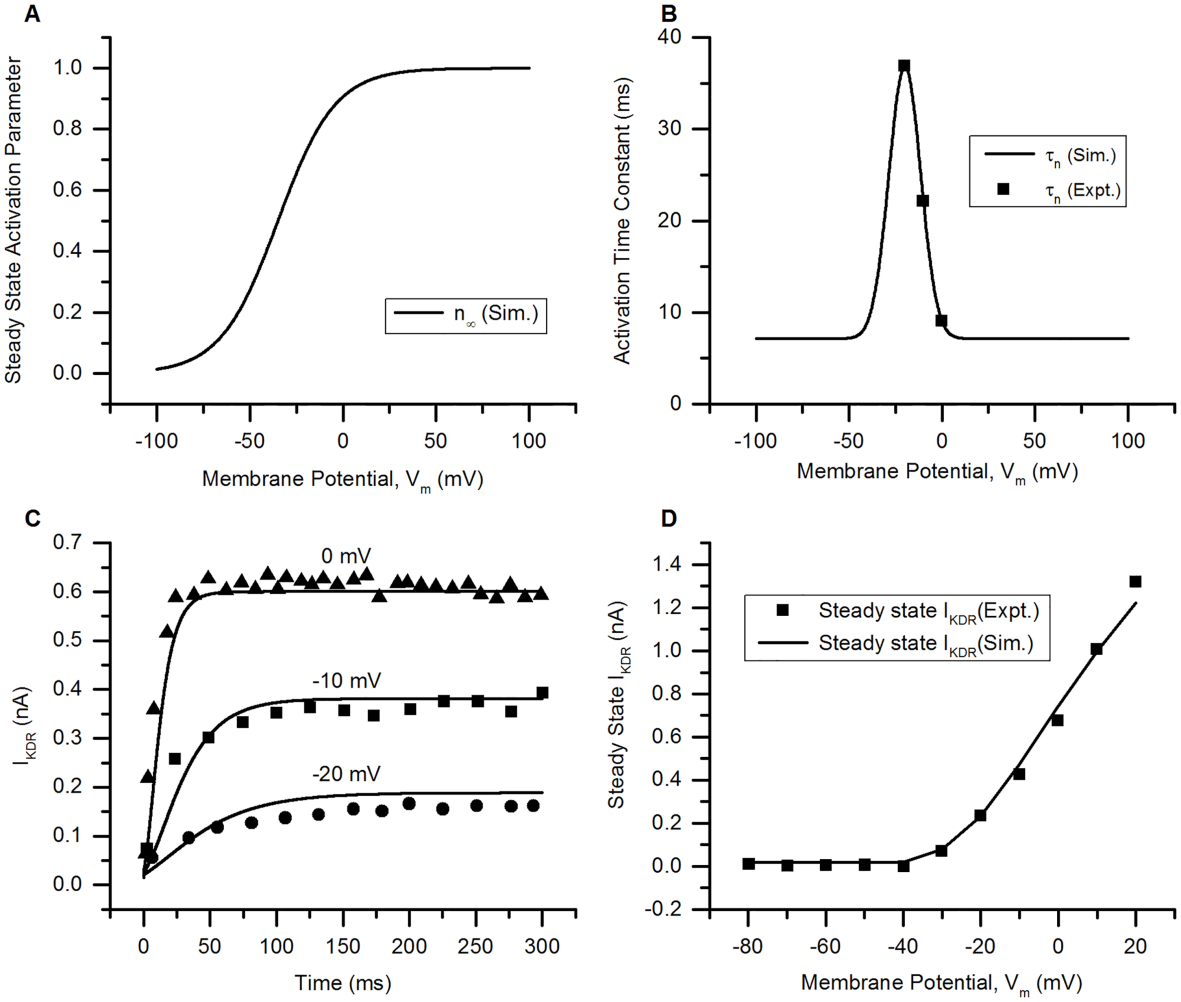
KDR channel. (A) Voltage dependence of steady state activation (*n*_∞_) of the modelled channel [21]. (B) The activation time constant (*τ*_*n*_) of the channel: solid line = simulation (Sim.) and squares = experimental data for bladder small DRG neurons (Expt.) from [33]. (C) The currents (*I*_*KDR*_) generated by the model (solid lines) and the corresponding currents reported in experiments (symbols, [7]) for bladder small DRG neurons using rectangular voltage clamp protocol. The holding potential was kept at −40 mV and test potentials of −20, −10 and 0 mV were applied for 300 ms. Experimental clamp currents: triangles (0 mV), squares = −10 mV and circles = −20 mV. (D) Steady state I-V relationship generated from model (solid line) and recorded from experiments (squares) for bladder small DRG neurons [33] by plotting the steady state current at end of each test potential. Protocol: Rectangular voltage clamp steps were applied from −80 to 10 mV in steps of 10 mV. The holding potential was −40 mV. Other model parameters: $\bar{g}=0.00072\;\text{S}/\text{cm}{}^{2}$, *E*_*K*_ = −84.7 mV, RMP = −53.5 mV, soma capacitance = 28 pF, soma diameter = 24 *μ*m. The S values for model fits and their 5% threshold values (given in brackets) are: for voltage clamp currents at: 0 mV = 0.045 nA (0.029 nA), −10 mV = 0.029 nA (0.016 nA) & −20 mV = 0.031 nA (0.006 nA), and I-V curve = 0.043 nA (0.066 nA).

*Delayed-rectifier K^+^ (KDR) Channels*. KDR channels are slowly-activating K^+^ channels compared to the transient K_A_ channels. KDR channels mostly contribute to the repolarizing phase of the membrane potentials during action potentials. The model was constructed by using the data given in [33] and [21]. Fig 4 shows the channel characteristics, step voltage clamp currents and I-V relationship for bladder small DRG neuron KDR channel model. The equations used for modelling this channel are:

$$n_{\infty }=\frac{1}{1+\text{exp}(\frac{-35-V_{m}}{15.4})}\;\tau_{n}=7.14+29.74\;\text{exp}(-2(\frac{V_{m}+20}{17.08}{)}^{2})$$ (19)

$$\frac{dn}{dt}=\frac{n_{\infty }-n}{\tau_{n}}\;I_{KDR}=\bar{g}n^{4}\left(V_{m}-E_{K}\right)\;\bar{g}=0.00072\;\text{S/cm}{}^{2},\;E_{K}=-84.7mV$$ (20)

*Ca^2+^-activated K^+^ (K_Ca_) channels*. K_Ca_ channels are activated by intracellular Ca^2+^ concentration ([Ca]_i_) and/or membrane potential (V_*m*_), and are an important regulator of excitability in some neurons and muscle cells. The Ca^2+^ influx from voltage-gated ion channels, activation of ligand-gated receptors and intracellular Ca^2+^ release mechanisms can modulate the activity of K_Ca_ channels. There are 3 types of K_Ca_ channels: the large-conductance (BK_Ca_) channels, the intermediate-conductance (IK_Ca_) channels and small-conductance (SK_Ca_) channels. These are classified on the basis of their single channel conductances: For SK_Ca_ conductances range between 5-20 pS, IK_Ca_ have a value of 10-60 pS whereas BK_Ca_ single channel conductance >100 pS [32]. BK_Ca_ channels are activated by membrane voltage and [Ca]_i_ while SK_Ca_ and IK_Ca_ are activated by [Ca]_i_ only [84]. However, recent studies have shown that SK_Ca_ channel exhibit inward rectification with respect to V_m_ [24, 26, 28]. SK_Ca_ channels have been shown to modulate the amplitude and duration of afterhyperpolarization (AHP) in some primary afferent neurons [31]. BK_Ca_ channel was included in the bladder small DRG neuron model along with SK_Ca_ channel. There was insufficient data for modelling IK_Ca_ channels for bladder DRG neurons and were not included in the model.

**Fig 5:**
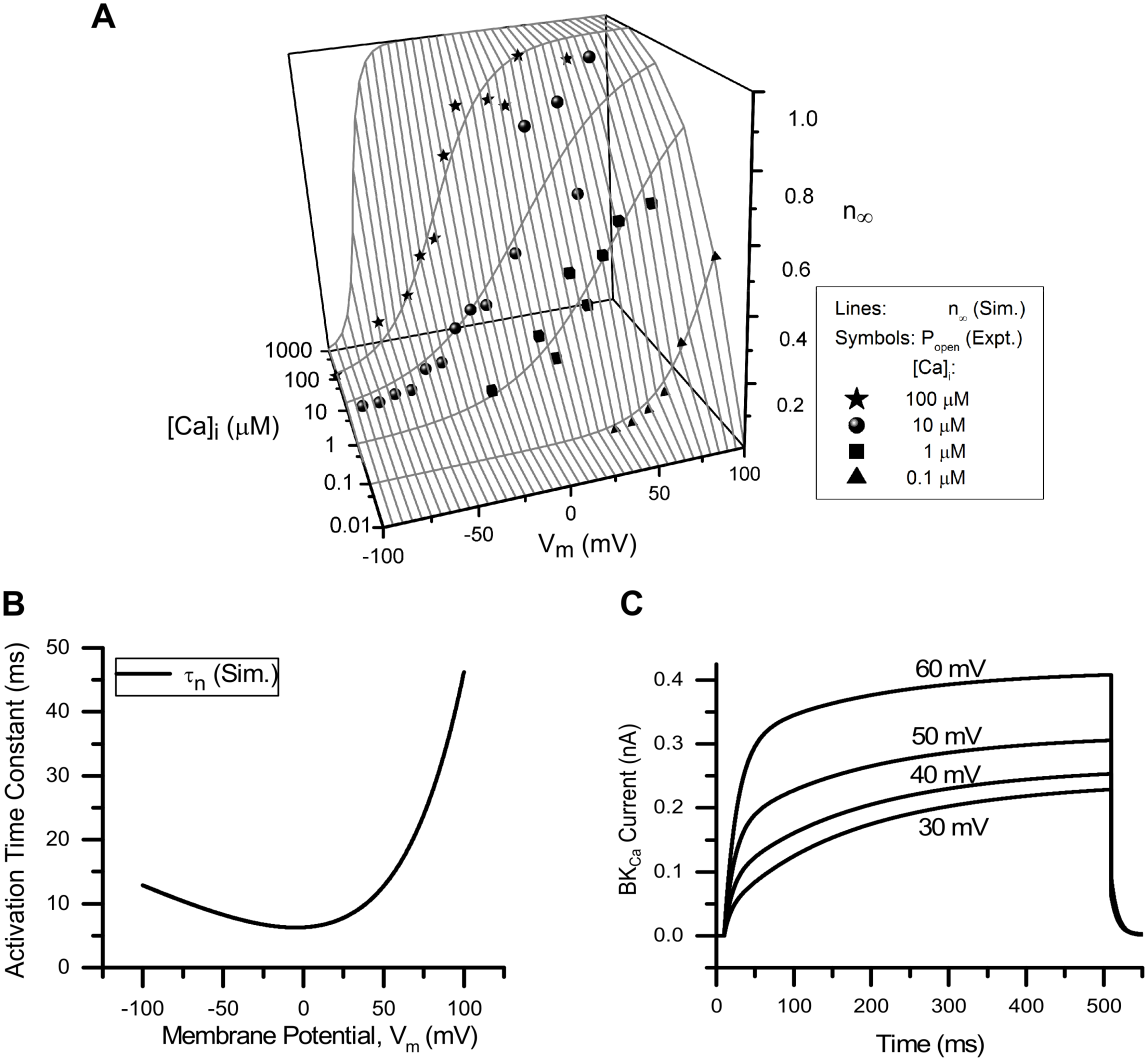
BK_Ca_ channel. (A) Voltage (V_m_)- and [Ca]_i_-dependence of steady state activation (*n*_∞_, solid lines) of the modelled channel (Sim. = Simulation). The symbols represent experimental data (Expt.) for open probability of the channel (P_open_) for different [Ca]_i_ [53]: triangles ([Ca]_i_ = 0.1 *μ*M), squares ([Ca]_i_ = 1 *μ*M), circles ([Ca]_i_ = 10 *μ*M) and stars ([Ca]_i_ = 100 *μ*M). Intracellular Ca^2+^ concentration is represented in units of *μ*M. (B) Time constants of activation (*τ*_*n*_) from the model (solid line). (C) Rectangular voltage clamp currents obtained from the model. Protocol: The holding potential was −53.5 mV. The test potentials from 30 to 60 mV, each of 500 ms duration were used in steps of 10 mV. Number above the current curves represent corresponding test potential. Other model parameters: $\bar{g}=0.00416\;\text{S}/\text{cm}{}^{2}$, initial [Ca]_i_ = 1.36*10^−4^ mM, E_Ca_ ∼ 122 mV, E_K_ = −84.7 mV, RMP = −53.5 mV, total capacitance = 28 pF and soma diameter = 24 *μ*m. The S values for *n*_∞_ for different [Ca]_i_ and their 5% threshold values (given in brackets) are: for 100 *μ*M = 0.070 (0.05), for 10 *μ*M = 0.121 (0.05), for 1 *μ*M = 0.079 (0.05) and for 0.1 *μ*M = 0.025 (0.05).

*Large-conductance Ca^2+^-activated K^+^ (BK_Ca_) channels*. Shieh et al. [52] showed that a BK_Ca_ channel blocker, A-272651 increased the AP duration and repetitive firing in L6-S1 spinal level capsaicin-sensitive small DRG neurons. Some of rat L6-S1 small DRG neurons supply the bladder [3, 85, 86]. Hence, we concluded that BK_Ca_ could also be present in bladder small DRG neurons and added BK_Ca_ channel to our model. The steady state activation (*n*_∞_) parameter was modelled as a function of [Ca]_i_ and V_m_. It was modelled using a Boltzmann equation in which the half activation value and slope factor are made a function of [Ca]_i_. The open channel probability data reported in [53] for small DRG neurons was used to model *n*_∞_. The open channel probability (P_open_) versus V_m_ measured for different [Ca]_i_ curves was taken as equivalent to the *n*_∞_ versus V_m_ (Fig 5A).

The time constants of activation (*τ*_*n*_) were calculated from the BK_Ca_ current curves for cutaneous DRG neurons [54] and were assumed [Ca]_i_ independent (Fig 5B). The equations used in the model are given below:

$$V_{1/2}=-43.4pCa-203\;sf=33.88\text{exp}(-(\frac{\left(pCa+5.42\right)}{1.85}{)}^{2})$$ (21)

$$pCa={\text{log}}_{10}\left({\left[Ca\right]}_{i}\right){\;(\left[Ca\right]}_{i}\;\text{in}\;\text{units}\;\text{of}\;\text{Molar)}\;\frac{dn}{dt}=\frac{n_{\infty }-n}{\tau_{n}}$$ (22)

$$n_{\infty }\left(V_{m},{\left[Ca\right]}_{i}\right)=\frac{1}{1+\text{exp}(\frac{V_{1/2}-V_{m}}{sf})}\;\tau_{n}=5.55\text{exp}(\frac{V_{m}}{42.91})+0.75-0.12V_{m}$$ (23)

$$I_{BKCa}=\bar{g}n\left(V_{m}-E_{K}\right)\;\bar{g}=0.00416\;\text{S}/\text{cm}{}^{2},\;E_{K}=-84.7\;\text{mV}$$ (24)

*Small-conductance Ca^2+^-activated K^+^ (SK_Ca_) channel*. The presence of SK_Ca_ channels in the bladder small DRG neurons was suggested by a study using SK_Ca_ channel positive modulator, NS4591 which reduced neuron’s spiking activity [30]. We modelled the SK3 subtype of SK_Ca_ based on the evidence by Bahia et al. [87] who found a higher expression of SK3 in small and medium-diameter DRG neurons.

SK_Ca_ channels are considered to be activated by [Ca]_i_ [84]. Soh and Park [24], however reported voltage-dependent inward rectification of rSK2 (rat SK2) currents with intracellular divalent cations. They found that divalent ions such as Ca^2+^, Mg^2+^ block SK_Ca_ channel pores by binding to its Ser-359 amino acid residue in voltage-dependent manner which results in this rectification [25]. Recently, Li and Aldrich [26] reported that rectification is inbuilt property of SK_Ca_ channels and is the result of electrostatic mechanisms with 3 charged residues in the S6 transmembrane domain of SK_Ca_ channel. I-V relationship of hSK3 (human SK3) channels in studies by Strøbæk et al. [55] and Hougaard et al. [88] also showed inward rectification in symmetrical K^+^ solutions ([K]_i_ = [K]_o_) as well as physiological K^+^ concentrations ([K]_i_ = 144 mM, [K]_o_ = 4 mM) [27].

**Fig 6:**
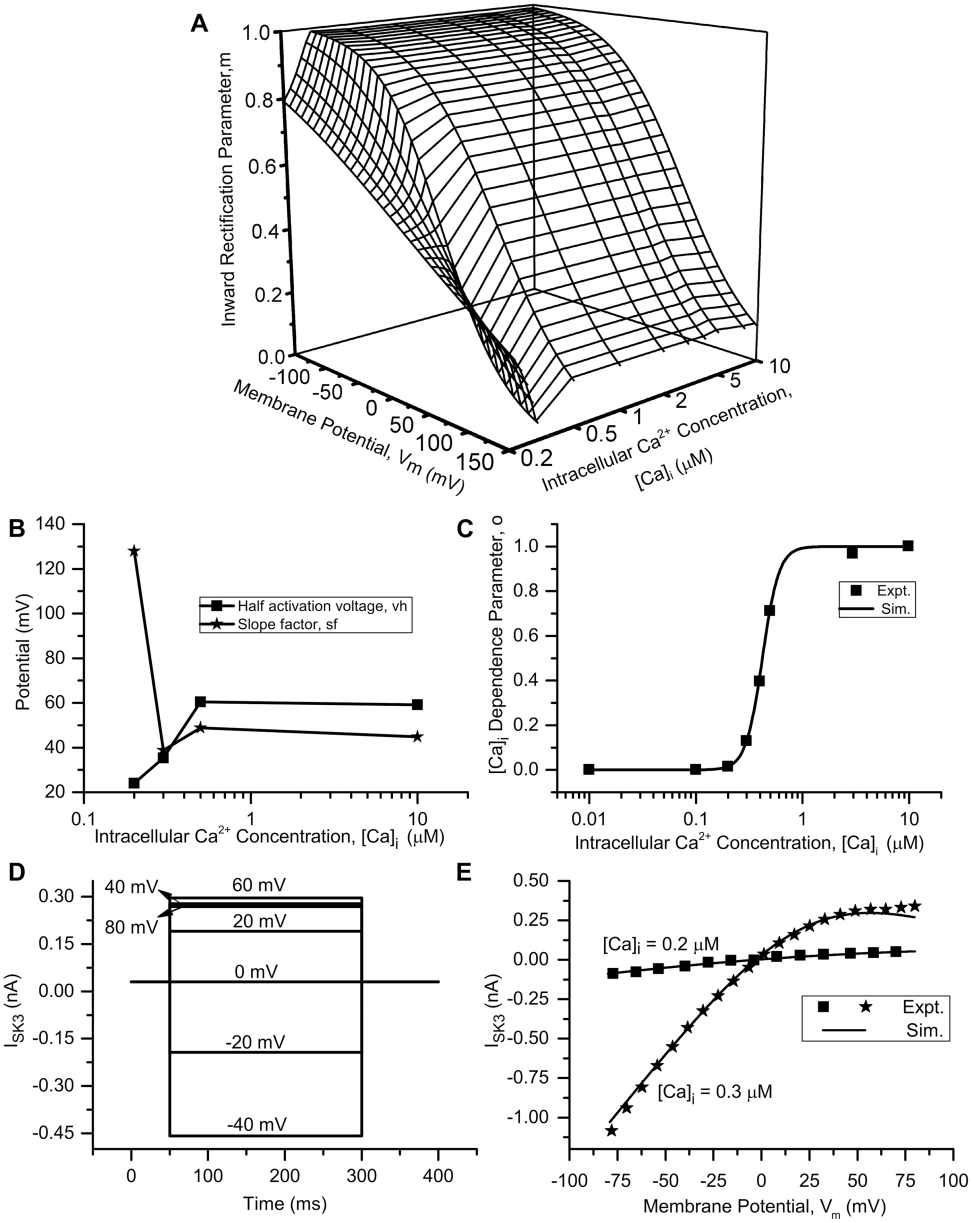
SK_Ca_ channel (SK3). (A) The voltage dependent inward rectification (IR) parameter, *m* as a function of [Ca]_i_ and membrane potential V_m_. (B) The [Ca]_i_-dependence of half activation and slope factor, *sf* of the parameter, *m*. (C) The relative current curve of hSK3 channels showing dependence of channel current on intracellular Ca^2+^ concentration ([Ca]_i_). This curve refers to Ca^2+^-dependent activation parameter, *o*. The squares represent the experimental data [55] and the Hill equation fit (used in model) is shown by the solid line. (D) The currents generated in rectangular voltage clamp protocol (numbers along currents represent corresponding voltage clamp test potentials). The holding potential was kept at 0 mV for 50 ms and the test potentials were applied from −40 to 80 mV in steps of 20 mV of 250 ms duration. [Ca]_i_ = 0.003 mM for rectangular voltage clamp. (E) Current-voltage (I-V) relationship generated by using ramp voltage clamp protocol. The current was recorded by applying a 200 ms voltage ramp starting at −80 to 80 mV from a holding potential of 0 mV. The solid lines represents the I-V relationship from simulation, and squares (for [Ca]_i_ = 0.2 *μ*M) and stars (for [Ca]_i_ = 0.3 *μ*M) represent the experimental data reported by [30, 55]. Other simulation parameters: $\bar{g}=0.0051\;\text{S}/\text{cm}{}^{2}$, [Ca]_o_ = 2 mM and E_*K*_ = −3 mV. The S values for model fits and their 5% threshold values (given in brackets) are: *o* = 0.019 (0.05), I-V curve for [Ca]_i_ of 0.2 *μ*M = 0.005 (0.007) and for [Ca]_i_ of 0.3 *μ*M = 0.023 (0.071).

The SK_Ca_ channel is modelled with two parameters: ‘*o*’ which defines the Ca^2+^-dependent activation, and ‘*m*’ which codes for voltage-dependent inward rectification. ‘*m*’ was modelled using a Boltzmann equation. Its half activation, *V*_1/2_ and slope factor, *sf* parameters were also found to vary with [Ca]_i_. Hence, ‘*m*’ was modelled as a function of both [Ca]_i_ and V_m_ (Fig 6A). An additional factor *E*_*K*_ (Nernst potential of K^+^) was added to the equation for ‘*m*’ (see below) to account for parameter shifts due to changing K^+^ concentrations. Data for modelling was obtained from [30] and [55].

$$o=\frac{{\left[Ca\right]}_{i}^{5.6}}{{\left[Ca\right]}_{i}^{5.6}+EC_{50}^{5.6}}\;m=\frac{1}{1+\text{exp}(\frac{V_{m}-\left(E_{K}+V_{1/2}\right)}{sf})}\;EC_{50}=0.42*10^{-3}\;\text{mM}$$ (25)

$$I_{SK3}=\bar{g}*o*m*\left(V_{m}-E_{K}\right)\;\bar{g}=0.0051\;\text{S}/\text{cm}{}^{2}\;E_{K}=-3\;\text{mV}$$ (26)

Data points for *V*_1/2_ and *sf* found from the experimental I-V curves in [30, 55] could not be fit to a curve. Hence, these parameters were modelled using a FUNCTION_TABLE feature of NEURON simulator, which calculates the values of variables from a table by linear interpolation of experimental values (see Fig 6B) [48].

Other K^+^ channels added to the model, KCNQ/M channel, Na^+^-activated K^+^ (K_Na_) channel, are described in S2 Text.

#### Ca^2+^ channels

Voltage-Gated Ca^2+^ (Ca_v_) channels are classified into two groups: low voltage-activated (LVA) and high voltage-activated (HVA), based on the activation thresholds. The T-type LVA channels start activating at V_m_ more depolarized than −70 mV hence contribute to resting state of the cell [60]. The HVA Ca^2+^ channels: L-type, N-type and P/Q-type have a higher threshold of activation. N-type channels start activating at −20 mV whereas L-type currents starts appearing for potentials > −10 mV in chick sensory neurons [60]. Contribution of L-type and N-type Ca^2+^ channels have been studied in small and medium-diameter bladder dorsal root ganglion neurons [89]. The T-type LVA channels expression is negligible as compared to HVA currents in both types of bladder afferent neurons [89, 90] and hence, their contributions to the model were kept minimal. N-type and L-type Ca^2+^ channels form the major components of the HVA currents in bladder small afferents and contribute to ∼40% and ∼35% of the total HVA current recorded at 0 mV rectangular voltage clamp [89] whereas the composition of the remaining Ca^2+^ current is unknown. This could be due to the presence of other Ca^2+^-conducting channels such as P/Q-type, R-type, T-type, TRPM8 and store-operated Ca^2+^ channels (SOCCs) whose presence has been shown by studies in bladder small DRG neurons and some other small DRG neurons [60, 64, 65, 70, 75, 76]. We added these channels to the soma model to account for the unknown Ca^2+^ current. Below are the descriptions L-type and N-type Ca^2+^ channels.

**Fig 7:**
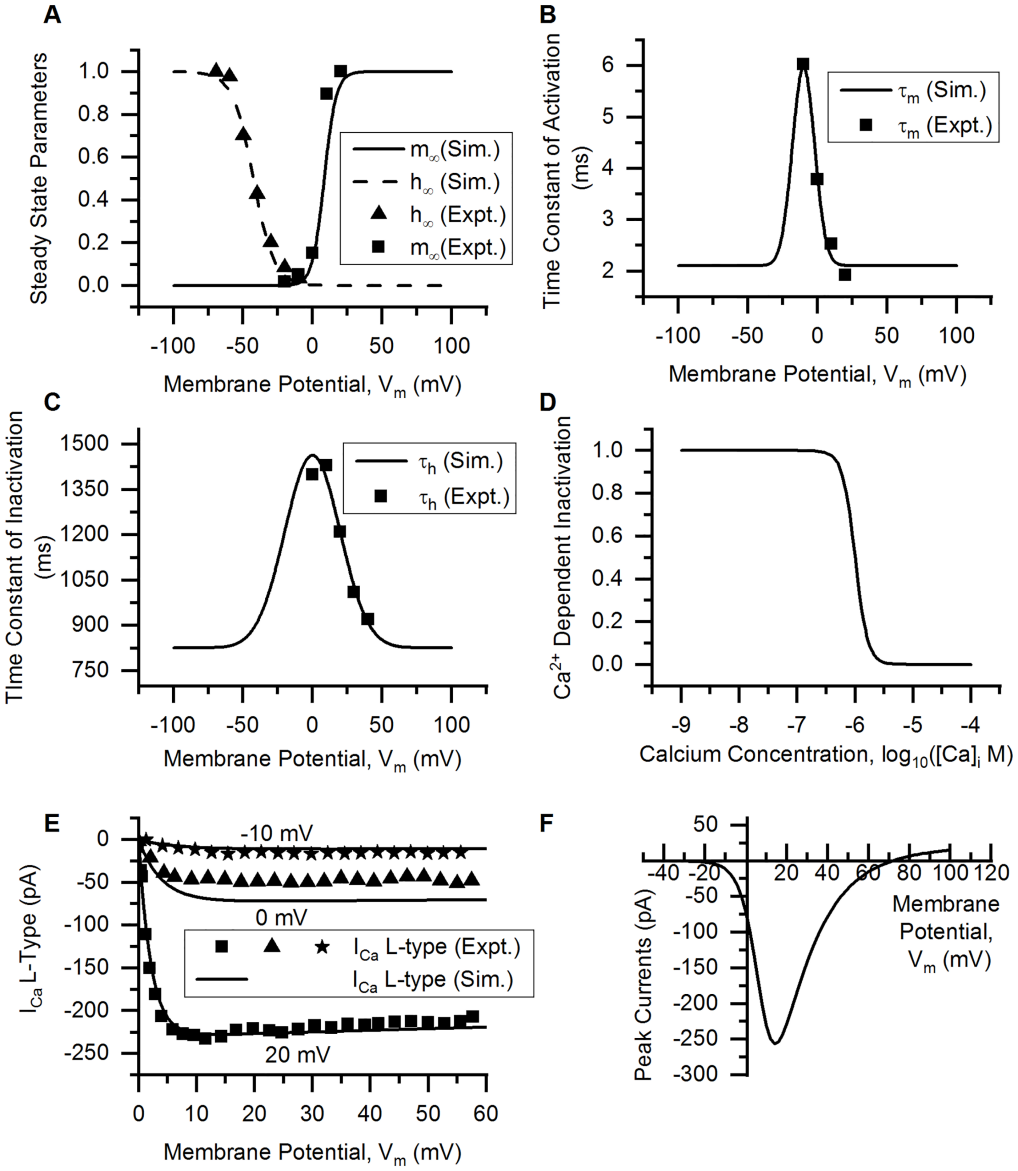
L-type Ca^2+^ channel. (A) Voltage dependence of steady state activation (*m*_∞_, solid line) and inactivation parameter (*h*_∞_, dashed line) of the modelled channel (Sim. = Simulation). Squares (*m*_∞_) and triangles (*h*_∞_) represent the experimental data (Expt. = Experimental data) from [60]. (B) Time constants of activation (*τ*_*m*_) from the model (solid line) and experiments (squares, [60]). (C) Time constants of inactivation (*τ*_*h*_) from the model (solid line) and experiments (squares, [60]). (D) Ca^2+^-dependent inactivation, *h*_*ca*_ is plotted against log_10_([Ca]_i_(in M)). (E) Rectangular voltage clamp currents obtained from the model (solid lines) and experiments (symbols, from [60]). Squares represent current at the test potential (t.p.) of 20 mV, triangles at t.p. = 0 mV, and stars at t.p. = −10 mV. The solid lines are the corresponding simulation results. Protocol: Holding potential (h.p.) was −40 mV. The t.p.’s were maintained for 70 ms. (F) Peak I-V relationship obtained from the model by rectangular voltage clamp protocol. The h.p. was kept at −40 mV and the test potentials from −80 to 100 mV, each of 780 ms duration were used. The peak inward current was recorded at each test potential. Other model parameters: *p*_*max*_ = 0.0113 cm/s, initial [Ca]_i_ = 136 nM, initial [Ca]_o_ = 0.037 mM, E_Ca_ ∼ 71 mV, RMP = −53.5 mV, soma capacitance = 28 pF, soma diameter = 24 *μ*m. The S values for model fits and their 5% threshold values (given in brackets) are: *h*_∞_ = 0.045 (0.048), for voltage clamp currents at: 20 mV = 4.423 pA (11.491 pA), −10 mV = 4.227 pA (0.848 pA) and 0 mV = 13.927 pA (2.51 pA).

*L-type Ca^2+^ (Ca_v_ 1) channels*. L-type (long-lasting) Ca^2+^ HVA currents activate around −10 mV [60] and inactive very slowly (inactivation time constant >700 ms) (Fig 7C). These channels are blocked dihydropyridine compounds like nimodipine, nisoldipine. The channel exhibits voltage-dependent and Ca^2+^-dependent inactivation (CDI) [60, 91]. The latter is encoded into the model as a sigmoid using a Hill equation (*h*_*ca*_). The data for modelling was obtained from [60] for chick small DRG neurons. CDI was adapted from [61] for Ca^2+^ N-type channel in uterine muscle cells. The equations used in the model are given below:

$$m_{\infty }=\frac{1}{1+\text{exp}(\frac{8.46-V_{m}}{4.26})}\;h_{\infty }=\frac{1}{1+\text{exp}(\frac{V_{m}+42.52}{7.48})}\;h_{ca}=\frac{1}{1+(\frac{{\left[Ca\right]}_{i}}{0.001}{)}^{4}}$$ (27)

$$\tau_{m}=2.11+3.86\text{exp}(-2(\frac{\left(V_{m}+10\right)}{16.02}{)}^{2})\;\tau_{h}=825.80+637.91\text{exp}(-2(\frac{V_{m}}{39.75}{)}^{2})$$ (28)

$$\frac{dm}{dt}=\frac{m_{\infty }-m}{\tau_{m}}\;\frac{dh}{dt}=\frac{h_{\infty }-h}{\tau_{h}}\;p_{max}=0.0113\;\text{cm/s}$$ (29)

$$I_{CaL-type}=p_{max}mhh_{ca}\frac{z^{2}F^{2}V_{m}}{RT}\frac{{\left[Ca\right]}_{i}-{\left[Ca\right]}_{o}\text{exp}(\frac{-zFV_{m}}{RT})}{1-\text{exp}(\frac{-zFV_{m}}{RT})}$$ (30)

Fig 7 shows the parameters, currents and I-V curve of the model.

*N-type Ca^2+^ (Ca_v_) 2.2 channels*. N-type Ca^2+^ channels activated close to −20 mV and inactivate with time constants between 50-100 ms [60]. Like, L-type Ca^2+^ channels, they can affect the duration of AP by changing its repolarizing phase. *ω*-conotoxin GVIA is a specific N-type Ca^2+^ channel blocker used to pharmacologically study these currents. The data for modelling was taken from studies on chick small DRG neurons [60, 62, 63]. CDI was modelled for this channel similar to L-type Ca^2+^ channel. These currents show incomplete inactivation in step voltage clamp experiments and appear to attain a steady state. Thus, a factor, ‘a’ (where 0 < a < 1) was introduced into the model to account for this partial inactivation of channels. The parameter ‘a’ limits the inactivation of currents to a certain value and after which the current stays at that as long as the stimulus is present. The equations used in the model are described below:

$$m_{\infty }=\frac{1}{1+\text{exp}(\frac{-6.5-V_{m}}{6.5})}\;h_{\infty }=\frac{1}{1+\text{exp}(\frac{V_{m}+70}{12.5})}\;h_{ca}=\frac{1}{1+{\left({\left[Ca\right]}_{i}/0.001\right)}^{4}}$$ (31)

$$\tau_{m}=0.8+5.38\text{exp}(-2(\frac{\left(V_{m}+20\right)}{15}{)}^{2})\;\frac{dm}{dt}=\frac{m_{\infty }-m}{\tau_{m}}\;\frac{dh}{dt}=\frac{h_{\infty }-h}{\tau_{h}}$$ (32)

$$I_{CaN}=p_{max}m\left(a*h+\left(1-a\right)\right)h_{ca}\frac{z^{2}F^{2}V_{m}}{RT}\frac{{\left[Ca\right]}_{i}-{\left[Ca\right]}_{o}\text{exp}(\frac{-zFV_{m}}{RT})}{1-\text{exp}(\frac{-zFV_{m}}{RT})}$$ (33)

$$p_{max}=0.0113\;\text{cm}/\text{s}\;a=0.7326$$ (34)

**Fig 8:**
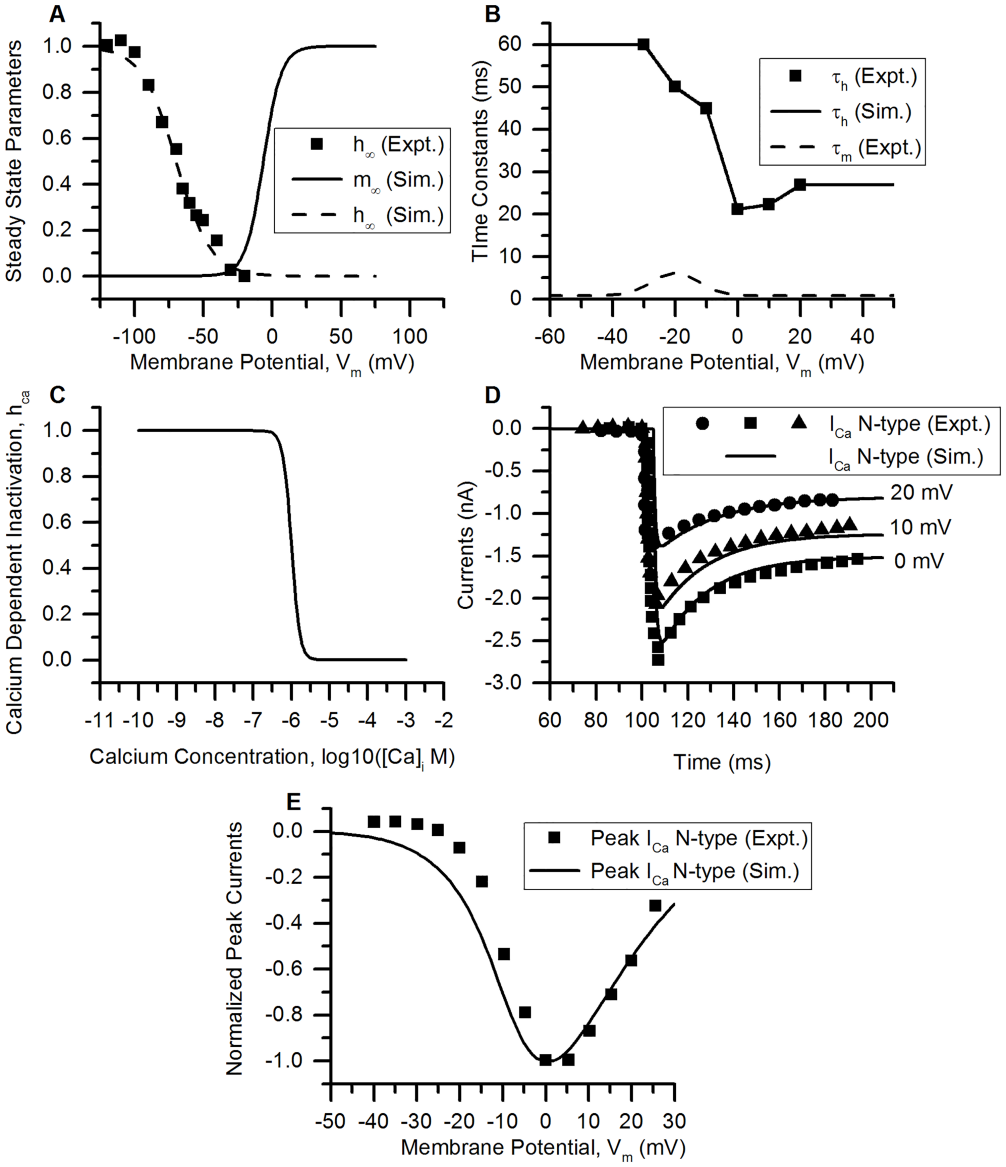
N-type Ca^2+^ channel. (A) Voltage dependence of steady state activation (*m*_∞_, solid line) and inactivation parameter (*h*_∞_, dashed line) of the modelled channel (Sim. = Simulation). Squares represent the experimental data for *h*_∞_ (Expt. = Experimental data) from [60]. (B) Time constants of activation (*τ*_*m*_, (dashed line) and inactivation (*τ*_*h*_, solid line) from the model. The squares represent experimental *τ*_*h*_ from [63]. (C) Ca^2+^-dependent inactivation parameter, *h*_*ca*_ is plotted against log_10_([Ca]_i_(in M)). (D) Rectangular voltage clamp currents obtained from the model (solid lines) and experiments (symbols, [63]). The squares represent current at test potential (t.p.) of 0 mV, the triangles and circles represent those from 10 mV and 20 mV, respectively. The solid lines are the corresponding simulation results. Protocol: The holding potential (h.p.) was −60 mV kept for 105 ms. The t.p.’s, were maintained for 100 ms. (E) The normalized peak I-V relationship obtained from the model (solid line) and experiments (squares, [63]) by rectangular voltage clamp protocol. The h.p. was kept at −60 mV for 100 ms and the test potentials from −50 to 30 mV, each of 100 ms duration were used. The curve was normalized by using the magnitude of peak current recorded at 0 mV. Other model parameters: *p*_*max*_ = 0.0113 cm/s, initial [Ca]_i_ = 136 nM, initial [Ca]_o_ = 0.017 mM, E_Ca_ ∼ 60 mV, RMP = −53.5 mV, soma capacitance = 28 pF and soma diameter = 24 *μ*m. The S values for model fits and their 5% threshold values (given in brackets) are: *h*_∞_ = 0.047 (0.05), I-V curve = 0.127 (0.052), for voltage clamp currents at: 0 mV = 1.636 nA (0.137 nA), 10 mV = 0.578 nA (0.1048 nA) and 20 mV = 0.446 nA (0.067 nA).

The time constant of inactivation (*τ*_*h*_) calculated from experimental data (Fig 8B, squares) could not be fit to a curve and the ‘FUNCTION_TABLE’ feature of the NEURON was used. The steady state parameters, time constants, step voltage clamp currents and normalized I-V relationship for N-type Ca^2+^ channels are shown in Fig 8.

P/Q-type Ca^2+^ channels, R-type Ca^2+^ channels, T-type Ca^2+^ channels, store-operated Ca^2+^ channels (SOCCs) and transient receptor potential cation channel subfamily M member 8 (TRPM8) are other Ca^2+^-permeable channels added to the model which are described in S2 Text. Descriptions for Na_v_1.9, KCNQ/M channels, K_Na_ channels, hyperpolarization-activated cyclic nucleotide-gated (HCN) channels, Ca^2+^-activated Cl^−^ channels (CaCCs), Na^+^/K^+^-ATPase pump and Na^+^/Ca^2+^ Exchanger (NCX) are also described in S2 Text.

### Ca^2+^ dynamics

The soma was divided into 12 concentric shells for encoding Ca^2+^ diffusion, tuning [Ca]_i_ and in creating separate Ca^2+^ concentration pools in the neuron [48]. Each shell has a separate intracellular Ca^2+^ concentration ([Ca]_i_), endoplasmic reticulum (ER) Ca^2+^ concentration ([Ca]_ER_), and mitochondrial Ca^2+^ concentration ([Ca]_MT_) representing a separate pool for each component in each shell (see S1 and S2 Figs). Mitochondrial volume in rat glabrous skin small-diameter DRG neurons has been estimated close to 7% (∼ 6.96%) of total cytoplasmic volume by [92]. ER can occupy >10% of the total cytosolic volume in eukaryotic cells [93]. Accordingly, we made mitochondria volume 7%, ER volume 12% and cytoplasmic volume to 81% of the total volume in each shell (S2 Fig). The number of shells were tuned to get a proper [Ca]_i_ transient in the outermost (towards plasma membrane) shell.

The cytoplasmic, mitochondrial and ER Ca^2+^ concentrations in the outermost shell were considered [Ca]_i_, [Ca]_MT_ and [Ca]_ER_, respectively of the bladder small DRG neuron soma model as the outermost shell receives Ca^2+^ from plasma membrane mechanisms and will have the maximum change in the concentrations. The outermost [Ca]_i_ concentration defines the electrochemical gradient for movement of Ca^2+^ ions across the cell membrane via the Ca^2+^ permeable channels and also activates the membrane Ca^2+^-activated K^+^ channels (BK_Ca_ and SK_Ca_), plasma membrane NCX and plasma membrane Ca^2+^-ATPase (PMCA) pump. Ca^2+^-activated Cl^-^ channels(CaCC) are activated by V_m_ and by local Ca^2+^ concentration release from inositol triphosphate receptors (IP3Rs) present on ER [72] in the outermost shell.

The rate of change of [Ca]_i_ in the outermost shell was given by the algebraic sum of Ca^2+^ flux from soma membrane Ca^2+^ permeable channels and exchanger (J_Membrane_), plasma membrane Ca^2+^ ATPase (J_PMCA_) pump, endoplasmic reticulum (ER) (J_ER_), mitochondria (J_MT_) and intracellular Ca^2+^ diffusion (J_Diffusion_):

$$\frac{\partial {\left[Ca\right]}_{i}}{\partial t}=\frac{1}{1+\beta }\left(J_{Membrane}-J_{PMCA}+J_{ER}+J_{MT}\right)+J_{Diffusion}$$ (35)

*β* is the buffer binding ratio of for the cell (discussed ahead) and codes for buffering of [Ca]_i_. For shells below the outermost shell, J_Membrane_ and J_PMCA_ are absent.

The changes in [Ca]_ER_ was modelled as:

$$\frac{d{\left[Ca\right]}_{ER}}{dt}=\frac{1}{1+\beta_{ER}}\left(J_{SERCA}-J_{IP3R}-J_{CICR}-J_{Leak}\right)$$ (36)

where *β*_*ER*_ is the buffering component of ER, J_SERCA_ is the flux through sarco-endoplasmic reticulum Ca^2+^ ATPase pump, J_IP3R_ is the flux due to IP3 receptor, J_CICR_ is the Ca^2+^ induced Ca^2+^ release (CICR) flux via the ryanodine receptors and J_Leak_ is the flux through leak channels on the ER. A negative flux sign denotes the decrease in [Ca]_ER_ with time as result of release of Ca^2+^ ions into cytoplasm and vice versa. Individual components of ER are discussed ahead.

The changes in the [*Ca*]_*MT*_ were modelled as:

$$\frac{d{\left[Ca\right]}_{MT}}{dt}=\frac{1}{1+\beta_{MT}}\left(J_{MCU}-J_{MNCX}\right)$$ (37)

where J_MCU_ is the mitochondria uniporter flux and J_MNCX_ is the flux due to mitochondrial Na^+^/Ca^2+^ exchanger(MNCX) and *β*_MT_ is the Ca^2+^ buffering component of mitochondria and was added to prevent the depletion of mitochondrial Ca^2+^ during high neuron firing. MNCX being a mitochondrial release mechanism, J_MNCX_ is negative and signifies a decrease in mitochondrial Ca^2+^.

#### Ca^2+^ buffering

Endogenous Ca^2+^ buffering in the cytoplasm, ER and mitochondria was modelled using Ca^2+^ binding ratio, *β* assuming rapid buffer approximation (RBA). *β* is the ratio of change in bound Ca^2+^ to change in free Ca^2+^:

$$\beta =\frac{d[Ca_{bound}]}{d[Ca]}=\frac{K_{B}{\left[B\right]}_{tot}}{{\left(K_{B}+\left[Ca\right]\right)}^{2}}$$ (38)

Here, [*Ca*] is either [*Ca*]_*i*_, [*Ca*]_*ER*_ or [*Ca*]_*MT*_, *K*_*B*_ is the dissociation constant of the buffer and [*B*]_*tot*_ is the total buffer concentration [94]. It gives how the free incoming Ca^2+^ via membrane mechanisms, diffusion for [*Ca*]_*i*_ or the Ca^2+^ influx via SERCA and MCU for ER and mitochondria, respectively decreases due to buffer action.

The parameters used for [Ca]_i_, [Ca]_ER_ and [Ca]_MT_ are given in Tables 3, 4 and 5. We used a value of *β* = 370 reported for rat small DRG neurons [95] for [*Ca*]_*i*_ buffering whereas *β*_*ER*_ and *β*_*MT*_ were calculated for [*Ca*]_*ER*_ and [*Ca*]_*MT*_ buffering using Eq 38.

**Table 3:** Ca^2+^ dynamics parameters.

Parameter	Symbol	Value	Reference
Diffusion Coefficient of Ca^2+^	*D*_*Ca*_	0.6 *μm*^2^/ms	[97]
Resting Intracellular Ca^2+^ Concentration	[Ca]_i_	1.36*10^−4^ mM	[44]
Resting ER Ca^2+^ Concentration	[Ca]_ER_	0.4 mM	[71, 98]
Resting Mitochondrial Ca^2+^ Concentration	[Ca]_MT_	2*10^−4^ mM	[99, 100]
Intracellular Ca^2+^ Buffer Binding Ratio	*β*	370	[95]
Resting Intracellular IP3 concentration	[IP3]_0_	1.60*10^−4^ mM	[98]
**PMCA Parameters**			
Pump Rate	*K*_1_	3.74*10^7^ /mM-s	Tuned
Pump Rate	*K*_2_	2.5*10^5^ /s	Tuned
Pump Rate	*K*_3_	500 /s	Tuned
Pump Rate	*K*_4_	5 /mM-s	Tuned
Initial Free Pump density	*pump*0	4.232*10^−13^ mol/cm^2^	Tuned

**Table 4:** ER Ca^2+^ dynamics parameters.

Parameters	Symbol	Value	Reference
**SERCA Pump Parameters**			
Maximal Pump Rate	*V*_*SERCA*_	3.75*10^−6^ mM/ms	Tuned
Dissociation Constant	*K*_*psr*_	0.00027 mM	[98]
**IP3 Parameters**			
Degradation Rate of IP3	*k*_*degrip*3_	0.00014 /ms	[98]
Diffusion Constant of IP3	*D*_*IP*3_	0.283 *μm*^2^/ms	[98]
**IP3 Receptor (IP3R) Parameters**			
Dissociation constant for IP3 binding to IP3R	*K*_*IP*3_	0.0008 mM	[98]
Dissociation constant for Ca^2+^ binding to IP3R activation site	*K*_*actip*3_	0.0003 mM	[98]
Rate of Ca^2+^ binding to IP3R inhibiting site	*k*_*onip*3_	2.7 /mM-ms	[98]
Rate of Ca^2+^ dissociation to the inhibiting site	*k*_*inhip*3_	0.0002 mM	[98]
Maximum Ca^2+^ flux from IP3R	$\bar{J_{IP3R}}$	3.5*10^−6^ mM/ms	[98]
**Ca^2+^-Induced Ca^2+^ Release (CICR)Parameters**			
Maximal release rate via RYR	*V*_*CICR*_	5*10^−7^ ms	Tuned
Dissociation constant for RYR	*K*_*CICR*_	0.00198 mM	[103]
Ca^2+^ Threshold for RYR activation	*K*_*TCICR*_	0.0006 mM	Tuned
**ER Buffer Parameters**			
Dissociation Constant of Buffer	*K*_*B*, *ER*_	0.5 mM	Tuned
Total Concentration of Buffer	[*B*]_*tot*, *ER*_	10 mM	Tuned

**Table 5:** Mitochondrial Ca^2+^ dynamics parameters.

Parameter	Symbol	Value	Reference
**Mitochondrial Uniporter (MCU) Parameters**			
Maximal MCU Uptake Rate	*V*_*MCU*_	1.4468*10^−6^ mM/ms	Tuned
Dissociation Constant of MCU	*K*_*MCU*_	6.06*10^−4^ mM	[106]
**Mitochondrial Na^+^/Ca^2+^ Exchanger (MNCX) Parameters**			
Maximum Release rate via MNCX	*V*_*MNCX*_	6*10^−5^ mM/ms	Tuned
Activation Constant for Na^+^	*K*_*Na*_	8 mM	[108]
Activation Constant for Ca^2+^	*K*_*Ca*_	0.035 mM	[108], Tuned
**Mitochondrial Buffer Parameters**			
Dissociation Constant of Buffer	*K*_*B*, *MT*_	1*10^−5^ mM	[99]
Total Concentration of Buffer	[*B*]_*tot*, *MT*_	0.065 mM	Tuned

#### Soma membrane flux

The change in [Ca]_i_ resulting from the soma membrane Ca^2+^ currents was taken into account by the following equation:

$$J_{Membrane}=\frac{I_{Ca}}{zFv}$$ (39)

where *I*_*Ca*_ is the Ca^2+^ current due to membrane mechanisms, *z* = 2 is the valence of Ca^2+^, *F* is the Faraday’s constant and *v* is the volume of the outermost shell [96].

#### Ca^2+^ diffusion

Radial 1-dimensional diffusion of Ca^2+^ across shells was modelled using Fick’s first law of diffusion. The flux from one shell to the adjacent is given by:

$$J_{Diffusion}=\frac{D_{Ca}}{1+\beta }\frac{A\Delta {\left[Ca\right]}_{i}}{\Delta r}$$ (40)

where *D*_*Ca*_ is the diffusion coefficient of Ca^2+^ in the cytoplasm, *β* is the cytoplasmic endogenous buffer binding ratio [94], A is the area of the partition between shells, Δ[*Ca*]_*i*_ is the concentration difference between shells and Δ*r* is the distance between shell centres [48].

#### Plasma membrane Ca^2+^-ATPase (PMCA) pump

The PMCA pump was modelled using the scheme:

$${\left[Ca\right]}_{i}+pump\overset{K_{1}}{\underset{K_{2}}{⇌}}pump{\left[Ca\right]}_{i}$$ (41)

$$pump{\left[Ca\right]}_{i}\overset{K_{3}}{\underset{K_{4}}{⇌}}pump+{\left[Ca\right]}_{o}$$ (42)

where *pump* is density of the unbound pump on the membrane (initial value = *pump*0, see Table 3), *pump*[*Ca*]_*i*_ is the bound density (initial value = [*Ca*]_*i*_ * *pump* * *K*_1_/*K*_2_), [*Ca*]_*o*_ is the extracellular Ca^2+^ concentration, *K*_1_, *K*_2_, *K*_3_ and *K*_4_ are reaction rate constants. *J*_*PMCA*_ is given as the difference of forward and backward flux of the second reaction [48].

#### ER mechanisms

The ER Ca^2+^ release and uptake mechanisms have been studied in small DRG neurons [101–103]. The mechanisms added to the model include SERCA pump, IP3 receptor, ryanodine receptor and ER leak channels.

#### SERCA pump

Like PMCA, these are low capacity, high-affinity Ca^2+^ pumps [96]. It helps in replenishing the Ca^2+^ store of the ER caused by the release of Ca^2+^ via IP3R and RYR. SERCA is an important regulator of cytoplasmic Ca^2+^ transients in small DRG neurons [101, 102]. It is modelled with a simple Hill equation [96, 98]:

$$J_{SERCA}=V_{SERCA}\frac{{\left[Ca\right]}^{n}}{{\left[Ca\right]}^{n}+{K_{psr}}^{n}}$$ (43)

where *V*_*SERCA*_ is the maximum pumping rate, *K*_*psr*_ is the dissociation constant of the pump, [Ca] is the Ca^2+^ concentration in the shell and n = 2 is the Hill constant.

#### Inositol 1,4,5-trisphosphate receptor (IP3R)

These receptors are present on the outer surface of the ER. These are activated by cytoplasmic inositol 1, 4, 5-trisphosphate (IP3) molecule and [Ca]_i_. The production of IP3 occurs via the activation of G-protein coupled receptors on the plasma membrane of the soma such as purinergic (P2Y) receptors and bradykinin receptors. The Ca^2+^ release from IP3Rs was based on a simplified model for IP3R [104] used by [98]. It was modelled as a function of the IP3 concentration ([IP3]) and [Ca]_i_ using the equation:

$$J_{IP3R}=\bar{J_{IP3R}}((\frac{\left[IP3\right]}{\left[IP3\right]+K_{IP3}}){\left(\frac{\left[Ca\right]}{\left[Ca\right]+K_{actip3}})h\right)}^{3}(1-\frac{\left[Ca\right]}{{\left[Ca\right]}_{ER}})$$ (44)

where $\bar{J_{IP3R}}$ is the maximum rate of release of Ca^2+^ from IP3 receptors, [IP3] and [Ca] is the concentration of IP3 and Ca^2+^ in the shell, *K*_*IP*3_ is the dissociation constant for IP3 binding to the IP3R, *K*_*actip*3_ is the dissociation constant for Ca^2+^ binding to activation site on the receptor, [Ca]_ER_ is the ER Ca^2+^ concentration in the shell and h is the probability of the inhibition site on IP3R being unoccupied and is given by the equation:

$$\frac{dh}{dt}=k_{onip3}\left(k_{inhip3}-\left(\left[Ca\right]+k_{inhip3}\right)h\right)$$ (45)

where *k*_*onip*3_ and *k*_*inhip*3_ are the rate of Ca^2+^ binding and dissociation to the inhibition site. The initial value of *h* is given by *k*_*inhip*3_/([*Ca*]+ *k*_*inhip*3_). The IP3 molecules undergo diffusion between the shells, and degradation to the resting concentration [*IP*3]_0_ at rate of *k*_*degrip*3_. The change in IP3 concentration in shells is given by the equation:

$$\frac{\partial [IP3]}{\partial t}=\frac{D_{IP3}A\Delta \left[IP3\right]}{\Delta r}-k_{degrip3}\left(\left[IP3\right]-{\left[IP3\right]}_{0}\right)$$ (46)

where *D*_*IP*3_ is the diffusion coefficient of IP3 in the cytoplasm, A is the area of the partition between shells, Δ[*IP*3] is the concentration difference between shells and Δ*r* is the distance between shell centres [98].

#### Ryanodine receptor (RYR)

RYRs are present on the surface of ER and are involved in Ca^2+^-induced Ca^2+^ release (CICR) in small DRG neurons [101, 103]. RYRs are activated by Ca^2+^ released on the nearby ER membrane via IP3Rs and other RYRs and also from the Ca^2+^ coming from the membrane mechanisms (Ca^2+^ channels). The RYR model presented here was adapted from CICR model of Purkinje cell [105]. The CICR flux (*J*_*CICR*_) is defined by the equation:

$$J_{CICR}=\{\begin{array}{ll} V_{CICR}\frac{\left[Ca\right]}{\left[Ca\right]+K_{CICR}}\left({\left[Ca\right]}_{ER}-\left[Ca\right]\right) & \text{if}\left[Ca\right]>K_{TCICR}\\ 0 & \text{if}\left[Ca\right]\leq K_{TCICR} \end{array}$$ (47)

where [*Ca*] is the Ca^2+^ concentration in the shell and *K*_*CICR*_ is the dissociation constant and *K*_*TCICR*_ is the activation threshold for RYR. The value of *K*_*CICR*_ was found by fitting the data on open probability of rat DRG neuron RyRs versus [Ca]_i_ given in [103].

#### ER leak channels

An ER leak channels were added to the model such that there is no net flux from the ER at rest [98]:

$$J_{ER,Leak}=L_{ER}(1-\frac{\left[Ca\right]}{{\left[Ca\right]}_{ER}})$$ (48)

where [*Ca*] is the Ca^2+^ concentration in the shell, L_ER_ leakage factor which is the sum of J_SERCA_, J_IP3R_ and J_CICR_ at resting state.

Table 4 gives the parameters for ER mechanisms.

#### Mitochondrial mechanisms

Mitochondria play an important role in regulating physiological [Ca]_i_ and Ca^2+^ transients in small DRG neurons [101, 106, 107]. Two mechanisms were modelled, viz. mitochondrial uniporter (MCU) for Ca^2+^ uptake and mitochondrial Na^+^/Ca^2+^ exchanger (MNCX) for release of [Ca]_MT_ to cytoplasm. The MCU and MNCX fluxes cancel each other at steady state [108]. The resting [Ca]_MT_ was considered to be 200 nM which is close to the intracellular Ca^2+^ concentration, [Ca]_i_ [100, 108].

#### Mitochondrial uniporter (MCU)

MCU helps to replenish [Ca]_MT_ lost via release through MNCX. It was modelled using data from [106]. Flux through MCU was modelled using a Hill equation dependence on [Ca]_i_:

$$J_{MCU}=V_{MCU}\frac{{\left[Ca\right]}^{n}}{{\left[Ca\right]}^{n}+{K_{MCU}}^{n}}$$ (49)

where *V*_*MCU*_ is the maximum pumping rate, *K*_*MCU*_ is the dissociation constant of the uniporter, [Ca] is the intracellular Ca^2+^ concentration in the shell and *n* is the Hill constant, which is 2.3 for MCU. The parameter values were obtained from [106] for small DRG neurons.

#### Mitochondrial Na^+^/Ca^2+^ exchanger

MNCX was modelled using a modified equation for plasma membrane Na^+^/Ca^2+^ exchanger (see S2 Text, Na^+^/Ca^2+^ Exchanger). The parameter values were adapted from [108]. The MNCX flux is described:

$$J_{MNCX}=V_{MNCX}\frac{{\left[Na\right]}_{i}^{3}}{{\left[Na\right]}_{i}^{3}+K_{Na}^{3}}\frac{{\left[Ca\right]}_{MT,s}}{{\left[Ca\right]}_{MT,s}+K_{Ca}}$$ (50)

where [*Ca*]_*MT*,*s*_ represents mitochondrial Ca^2+^ concentration in shell and [Na]_i_ is Na^+^ concentration in the cytoplasm, *V*_*MNCX*_ is maximal NCX activity, *K*_*Na*_ and *K*_*Ca*_ are constants for Ca^2+^ and Na^+^, respectively.

Table 5 has the values of parameters for mitochondrial mechanisms.

#### Effects of calcium indicator dyes

Calcium indicator dyes used for imaging intracellular/ER/mitochondrial calcium concentrations can act as calcium buffers themselves and alter the amplitude and decay phase of the calcium transient [94, 109, 110]. These effects were taken into account while comparing the simulation and experimental calcium imaging data for cytoplasmic and mitochondrial calcium.

The calcium imaging dye and [*Ca*]_*i*_ are assumed to be always at equilibrium and follow the rapid buffer approximation. Hence, Eq 35 for rate of change of [*Ca*]_*i*_ was modified for an imaging dye with buffer biding ratio, *β*_*dye*_ as:

$$\frac{\partial {\left[Ca\right]}_{i}}{\partial t}=\frac{1}{1+\beta +\beta_{dye}}\left(J_{Membrane}-J_{PMCA}+J_{ER}+J_{MT}\right)+J_{Diffusion}$$ (51)

The presence of an calcium imaging dye in the cytoplasm also affects the intracellular diffusion of calcium [94, 109]. The diffusion coefficient of free calcium in the cytoplasm, *D*_*Ca*_ was replaced by the effective diffusion coefficient, *D*_*eff*_ in the diffusion flux Eq 40:

$$J_{Diffusion}=D_{eff}\frac{A\Delta {\left[Ca\right]}_{i}}{\Delta r}$$ (52)

where *D*_*eff*_ is given by the expression:

$$D_{eff}=\frac{D_{Ca}+\beta_{dye}D_{dye}}{1+\beta +\beta_{dye}}$$ (53)

*D*_*dye*_ is the diffusion coefficient of the dye and *β* is the buffer binding ratio of the endogenous buffer.

For [*Ca*]_*i*_ validation where normalized simulated [*Ca*]_*i*_ was compared with that from imaging data [111] using 30 mM [K]_o_, Fura-2 was used as an imaging dye with parameters [*B*]_*tot*_ = 5 *μ*M [111], *K*_*B*_ = 224 nM [112] and *D*_*dye*_ = 0.1 *μ*m^2^/s (for free dye diffusion) [113]. *β*_*dye*_ was calculated by Eq 38. Additional effect of dye buffering lead to change in resting [*Ca*]_*i*_ and was stabilized by tuning the initial PMCA pump density (*pump*0) 4.08*10^−13^ mol/cm^2^.

For [*Ca*]_*i*_ transient elicited by 1 AP, Indo-1 calcium indicator dye was used by [44] for the imaging experiments. The buffering parameters used in the model for Indo-1 are [*B*]_*tot*_ = 100 *μ*M [44], *K*_*B*_ = 250 nM [112] and *D*_*dye*_ = 0.1 *μ*m^2^/s (for free dye diffusion) [113]. *pump*0 was tuned to 4.0815*10^−13^ mol/cm^2^ for stabilizing the initial [*Ca*]_*i*_.

For mitochondrial calcium dynamics validation, the dye used for imaging [*Ca*]_*i*_ in experiments [106] was Fura-FF with [*B*]_*tot*_ = 200 *μ*M [106], *K*_*B*_ = 5500 nM [106] and *D*_*dye*_ = 0.075 *μ*m^2^/s (assumed). The changes in [*Ca*]_*MT*_ of the small DRG neurons presynaptic terminals were monitored by using mtPericam overexpressed in the mitochondria [106]. The Eq 37 for rate of change of [*Ca*]_*MT*_ was modified as:

$$\frac{d{\left[Ca\right]}_{MT}}{dt}=\frac{1}{1+\beta_{MT}+\beta_{dye}}\left(J_{MCU}-J_{MNCX}\right)$$ (54)

The parameters used for calculating *β*_*dye*_ for mtPericam in simulations are: [*B*]_*tot*_ = 15 *μ*M (tuned) and *K*_*B*_ = 1700 nm [114].

## Results

### Validation of the model

#### Action potentials

**Fig 9:**
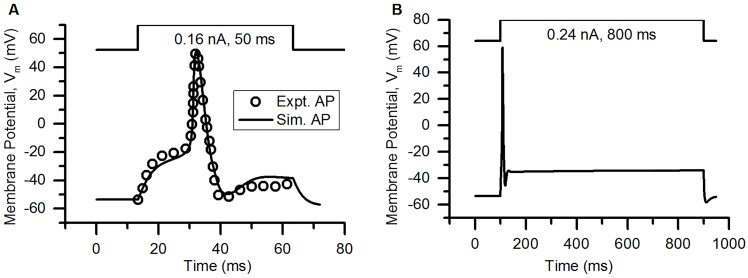
Action potentials in the bladder small DRG neuron soma model for different stimuli. (A) AP generated by a current clamp of 0.16 nA and 50 ms duration for experiments by Hayashi et al. [36] (circles, Expt. AP) and simulated AP (solid line, Sim. AP). The standard error in regression, S for the fit and 5% threshold values (in brackets) are 4.43 mV (5.4 mV) indicating a good fit. (B) Response to long duration (800 ms) current clamp of amplitude 0.24 nA.

The action potential of the bladder small DRG neuron model elicited by a rectangular current pulse (0.16 nA, 50 ms) is shown in the Fig 9A (Sim. AP) and is compared with an experimental AP (Expt. AP) [36] for the same stimulus. Fig 9B shows the model’s response for a long duration (800 ms) rectangular current clamp of 0.24 nA. A single action potential was observed in model to such stimulus which is in agreement with similar studies in experiments [3, 10, 33, 49]. Action potential properties for simulated and experimental APs are compared in the Table 6.

**Table 6:** Comparison of simulated and experimental APs in Fig 9.

Properties	Simulation	Experiment
AP Overshoot	50.91 mV	53.3 mV
Resting Membrane Potential (RMP)	−53.56 mV	−53.5 mV
AP Amplitude (from RMP)	104.47 mV	106.8 mV
AHP Peak	−49.83 mV	−54.7 mV
AP Duration	4.52 ms	4.56 ms
Number of Spikes for 800 ms depolarization	1	1

AP Overshoot is maximum potential above 0 mV during an AP. Resting Membrane Potential is the V_m_ just before the start of the stimulus. AP Amplitude is the difference in V_m_ between RMP and AP Peak. AHP peak is minimum potential reached during an AHP. AP Duration is the half width of AP and is the duration between 2 points on an AP when V_m_ reaches half the AP Amplitude.

As evident from Table 6, the experimental and simulated AP exhibit a close similarity. The goodness-of-fit of the experimental and simulated APs (Fig 9A, S value = 4.43 mV) shows that the model closely captures the action potential properties of the in-vitro preparation.

#### Subthreshold potentials

**Fig 10:**
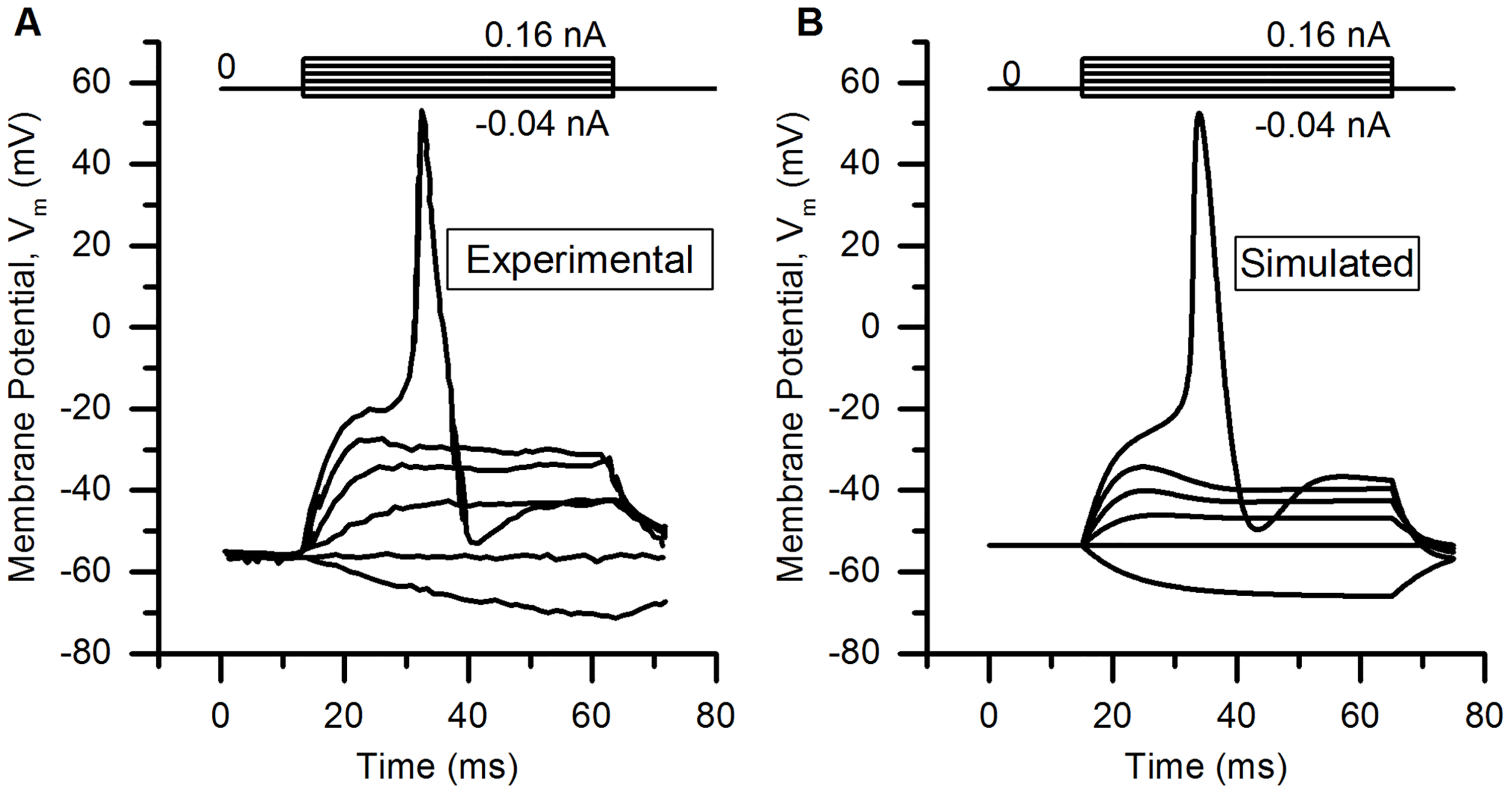
Comparison of experimental and simulated subthreshold potentials in bladder small DRG neuron soma model. (A) Experimental changes in V_m_ generated by applying 50 ms rectangular current clamps of amplitudes ranging between −0.04 nA to 0.16 nA in steps of 0.04 nA as shown in (data from [36]). (B) Response of the model for similar stimuli. Note: one AP is shown in each panel above the subthreshold responses for the sake of comparison.

In order to see if the subthreshold responses of the model are similar to those observed experimentally, we compared the responses of the model on applying current clamps with amplitudes insufficient to evoke an AP. As shown in Fig 10, the subthreshold responses obtained in our model were comparable to those seen experimentally.

#### Cytoplasmic Ca^2+^ dynamics

**Fig 11:**
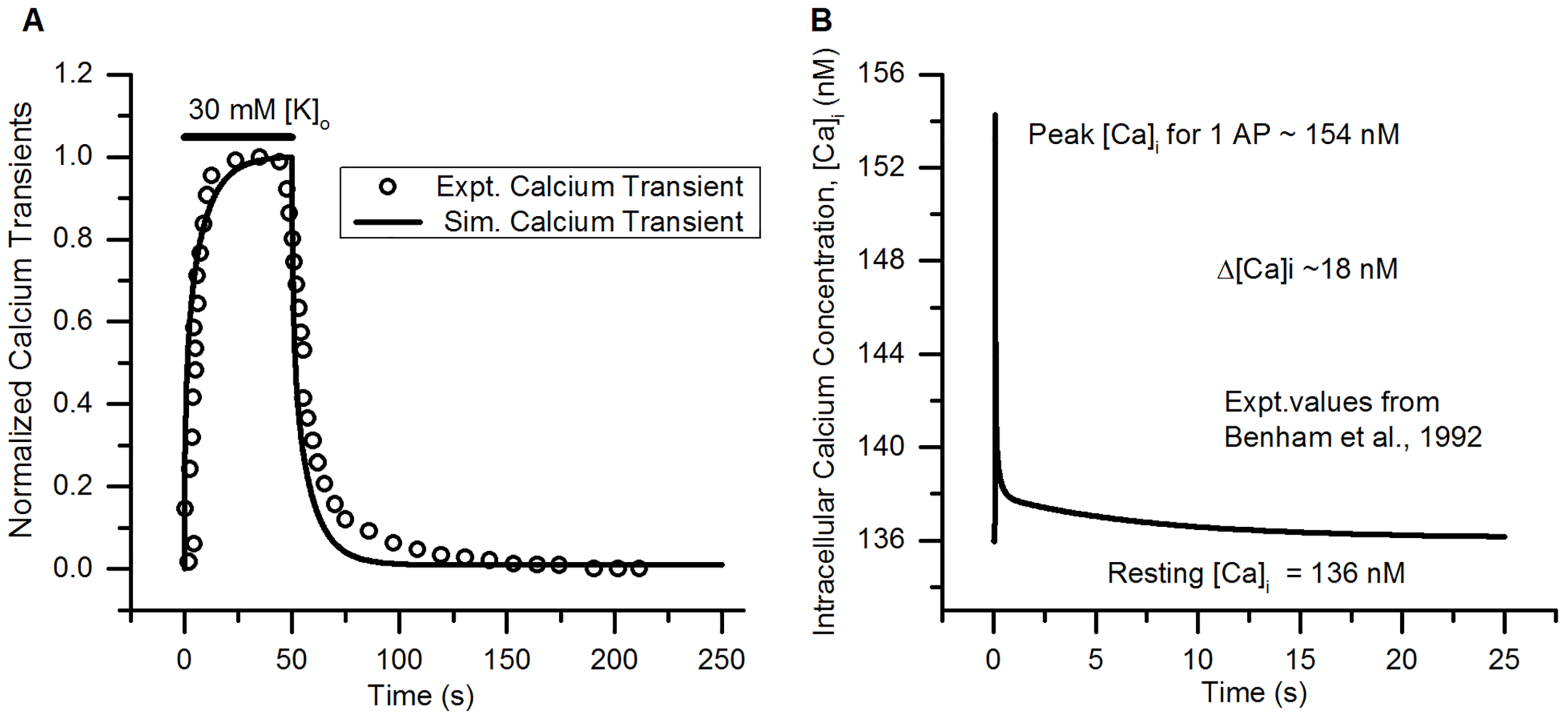
Ca^2+^ transients in bladder small DRG neuron soma model. (A) Comparison of normalized Ca^2+^ transient obtained from bladder small DRG neurons experimentally (circles, Expt. Calcium Transient, [111] obtained from fluorescence studies) and our model (Solid line, Sim. Calcium Transient) by application of high [K]_o_ (30 mM) for 50 seconds (indicated by bar). The S value for the fit and the 5% threshold (in brackets) is 0.2 (0.05). (B) Ca^2+^ transient for a single AP in the model. AP was generated by 2 nA, 1 ms rectangular current clamp pulse. The model was held at −80 mV for 50 ms before generating the AP. The data for resting [Ca]_i_ and change in [Ca]_i_ (Δ[Ca]_i_) was obtained from [44] for small DRG neurons. Effects of calcium dye buffering were included in the simulations.

Fig 11 compares the normalized Ca^2+^ transient evoked by application of 30 mM [K]_o_ in bladder small DRG neurons generated in experiments [111] and by our model. Benham et. al. [44] reported values for resting [Ca]_i_ and change in [Ca]_i_ for AP firing in small DRG neurons. Change in [Ca]_i_ of ∼20 ± 3 nM was reported for a single AP. Model was tuned to generate an increase (∼18 nM) during an AP as low-voltage activated T-type calcium channel have only a small contribution to calcium current in bladder small DRG neurons [90]. Therefore, the [Ca]_i_ rises from its base value of 136 nM [44] to 154 nM during an AP (Fig 11B). Effects of calcium dye buffering have been incorporated into the model to compare simulation results with experimental data. See “Effects of calcium indicator dyes” in Methods.

#### Mitochondrial Ca^2+^ dynamics

**Fig 12:**
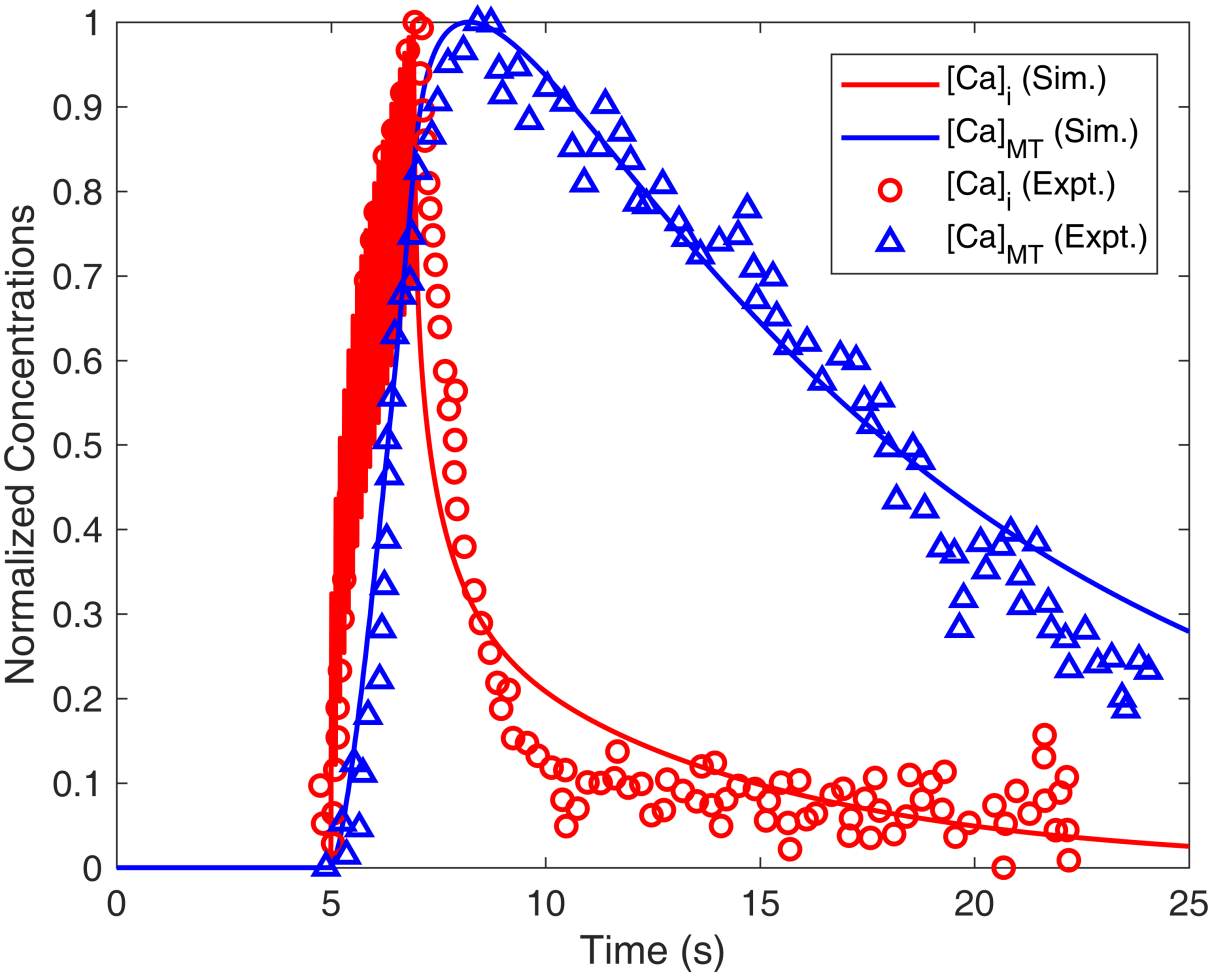
Comparison of normalized experimental and simulated [Ca]_i_ and [Ca]_MT_. The solid red line and solid blue line represent the simulated [Ca]_i_ and [Ca]_MT_, respectively from the model (Sim.), obtained by evoking 20 APs at 10 Hz. APs were evoked by using rectangular current clamps of 1.25 nA and 1 ms. The red circles and blue triangles are experimental traces digitized (Expt.) [Ca]_i_ and [Ca]_MT_ for similar stimuli in small DRG neuron terminals obtained from [106]. The rising and falling dynamics of our bladder small DRG soma model output closely compares with those recorded experimentally. The S value for [Ca]_i_ and [Ca]_MT_ fits and their 5% thresholds (in brackets) are 0.12 (0.05) and 0.068 (0.05), respectively. Effects of calcium dye buffering were included in the simulations.

Due to absence of data on mitochondrial Ca^2+^ concentrations ([Ca]_MT_) for bladder small DRG neurons, we used the data for small DRG neuron presynaptic terminals reported by [106]. Fig 12 shows the comparison of normalized [Ca]_i_ and [Ca]_MT_ from experiments [106] and corresponding normalized concentrations obtained from our model. The changes in [Ca]_i_ and [Ca]_MT_ were evoked by 20 action potentials fired at 10 Hz identical to the experimental stimulus. The comparison shows that the model is able to capture the rising and falling phase of the [Ca]_i_ and [Ca]_MT_ and their time courses closely. Effects of calcium dye buffering have been incorporated into the model to compare simulation results with experimental data. See “Effects of calcium indicator dyes” in Methods.

The match between experimentally observed cytoplasmic and mitochondrial Ca^2+^ dynamics on the one hand and corresponding model outputs on the other help repose confidence in the model, including the interaction of electrical and Ca^2+^ dynamics components.

#### Role of PMCA, SERCA and mitochondria on Ca^2+^ dynamics

**Fig 13:**
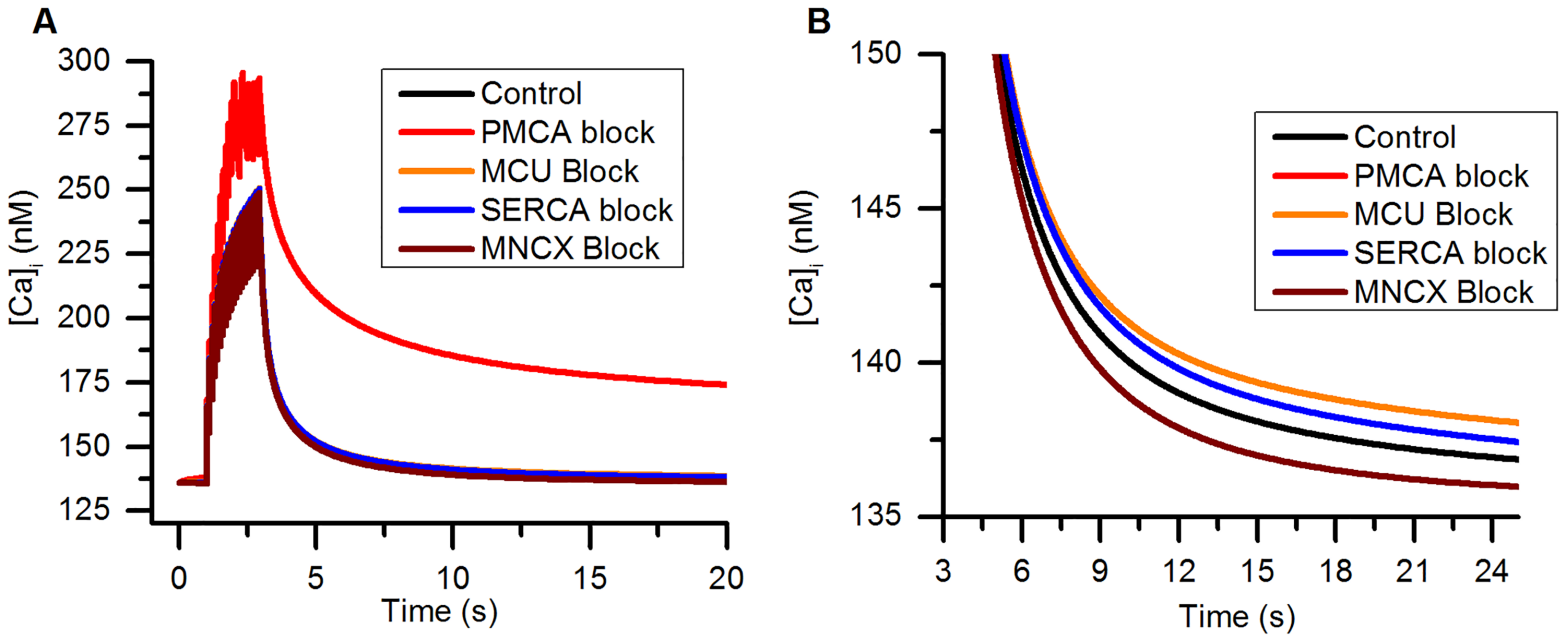
Role of PMCA, MCU, SERCA and MNCX in shaping the Ca^2+^ transient. (A) [Ca]_i_ levels for the response of the model to 20 APs generated by 1 ms, 1.25 nA rectangular current clamps given at 10 Hz. PMCA (red), MCU (orange), SERCA (blue) and MNCX (maroon) are blocked individually. Black curve is the control [Ca]_i_ (B) Magnified scale to show the effects of these components on the decay of Ca^2+^ transients.

PMCA pump, MCU and SERCA pump are the major Ca^2+^ removal mechanisms in small DRG neurons [101, 106, 107, 115]. Fig 13 shows the Ca^2+^ transient obtained in response to 20 rectangular current clamps of 1 ms, 1.25 nA applied at 10 Hz in control conditions (black), with PMCA block (red), MCU block (orange), SERCA block (blue) and MNCX block (maroon). Blocking PMCA pump, MCU and SERCA increased the decay time of transients (Fig 13A and 13B). A marked effect was seen when PMCA was blocked, which enhanced both the amplitude of [Ca]_i_ and its decay time similar to observations of [115] for small DRG neurons. Mitochondrial Ca^2+^ uptake is an important Ca^2+^ removal mechanism in small DRG neurons [106, 116] which is seen in our model by blocking MCU. Block of the mitochondrial release mechanism, MNCX, elicits a faster decay of Ca^2+^ transient to resting [Ca]_i_. Block of SERCA also increases the duration of the Ca^2+^ transients [101, 102].

The contribution of ER release mechanisms (IP3R and RYR) in DRG neurons are negligible at the resting potential and for small rises in the [Ca]_i_ and smaller depolarizations [101, 117], due to insufficient [Ca]_i_ and IP3 molecules for the activation of RYR and IP3R. Blocking these components in the model did not lead to any change in the rise and decay phase of the [Ca]_i_. The similarities of the modelled Ca^2+^ dynamics (which includes Ca^2+^ diffusion, buffering, ER mechanisms, mitochondrial mechanisms, PMCA, NCX and the Ca^2+^ channels) to the corresponding experimental waveforms suggest that our model is capable of mimicking to a good degree the Ca^2+^ dynamics of the biological small DRG neuron.

### Effect of SK_Ca_ inward rectification

#### Single APs: Decrease in AHP duration

To the best of our knowledge, computational models till date model SK_Ca_ with [Ca]_i_-dependent activation only and no inward rectification. Such an approach will clearly result in inaccurate model outputs, both for the SK_Ca_ conductance itself and for other model parameters that are influenced by it. We therefore, set out to model the voltage-dependent inward rectification along with [Ca]_i_-dependence and also validated our model against experimental data (see Fig 6).

The effects of SK_Ca_ were tested by comparing a bladder small DRG neuron soma model containing an SK_Ca_ conductance possessing inward rectification with a soma model in which SK_Ca_ inward rectification was absent. The effect of SK_Ca_ inward rectification on a single AP (at baseline SK_Ca_ conductance) was minimal. Comparison of APs from the two models did not show much difference, probably because of the almost linear I-V relationship for small increases in [Ca]_i_ (20-30 nM for 1 AP) of the models. Hence, we tested the effect of an elevated value of SK_Ca_ conductance. A higher than normal SK_Ca_ conductance (or current) in DRG neurons could represent conditions during application of SK_Ca_ channel openers and positive modulators such as 1-EBIO and NS4591 [30]. Abnormal conditions such as inflammation can lead to increased basal [Ca]_i_, higher amplitude and slower decay of Ca^2+^ transients [118], which can also enhance SK_Ca_ conductance.

**Fig 14:**
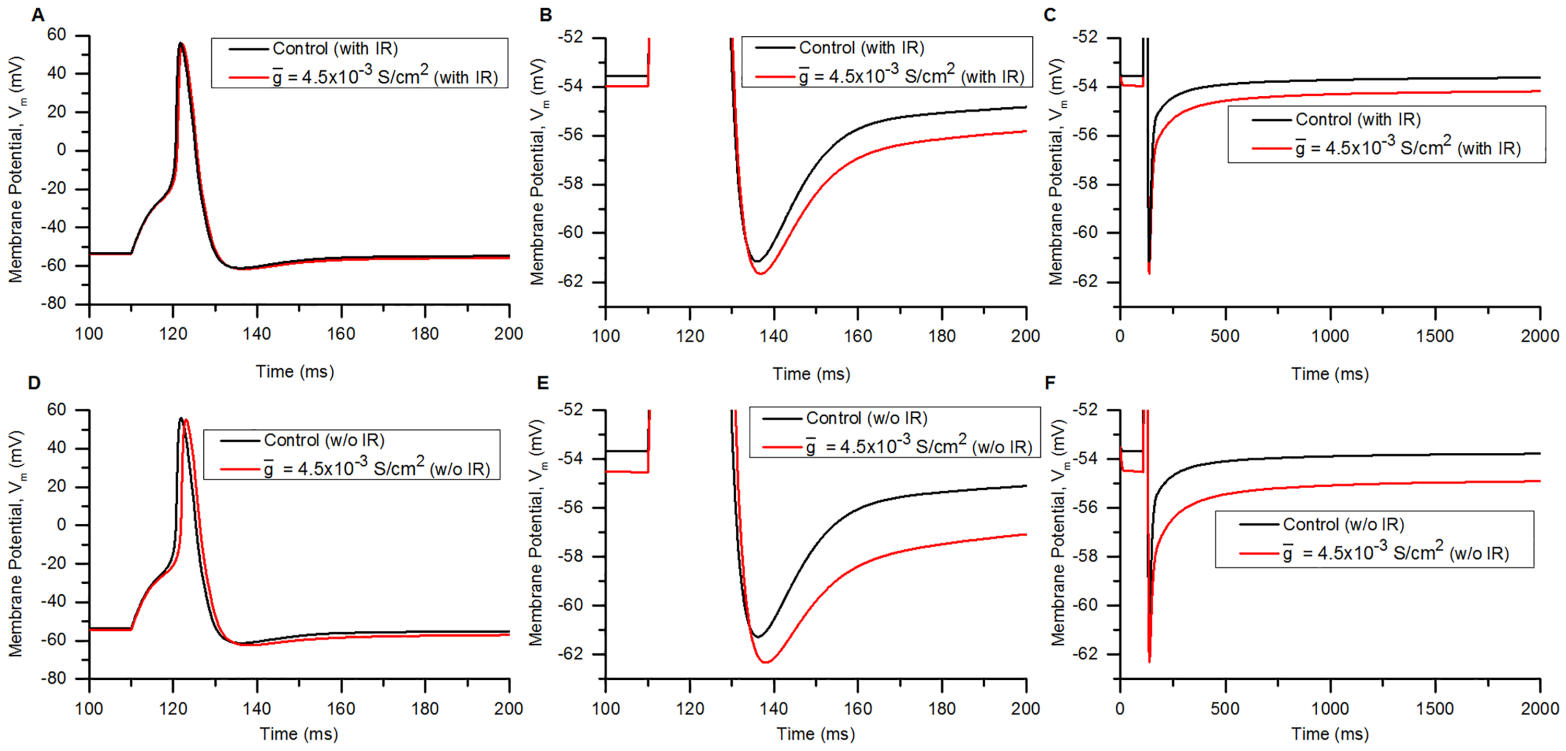
Effect of SK_Ca_ inward rectification on AP. (Upper panel, A-C) AP generated in bladder small DRG neuron soma model with SK_Ca_ possessing inward rectification (with IR). (Lower panel, D-F) AP simulated in a soma model in which SK_Ca_ channel is without inward rectification (w/o IR). (A and D) Effect of increasing the maximum conductance of SK_Ca_ ($\bar{g}$) from the baseline value 9*10^−4^ S/cm^2^ (Control, black) to 4.5*10^−3^ S/cm^2^ (red). (B and E) RMP and AHP Amplitude of (A) and (D) figures are shown on an expanded time and voltage scale. (C and F) The recovery phase of the AHP. Corresponding top and bottom comparisons show that SK_Ca_ inward rectification has an important role in changing RMP, AP amplitude and AHP. Stimulus: rectangular current clamp of 0.18 nA for 15 ms given at 110 ms.

Fig 14 compares AP properties in the neuron model that has an SK_Ca_ channel endowed with inward rectification (Fig 14A–14C) with those in the model without inward rectification (Fig 14D–14F). APs were generated by using a rectangular current clamp of 0.18 nA, 15 ms [49]. The effect was compared by increasing the (maximum conductance) of SK_Ca_ ($\bar{g}$) from its control value of 9*10^−4^ S/cm^2^ (Fig 14, black) to 4.5*10^−3^ S/cm^2^ (Fig 14, red) for both the models. Differences can be seen in the RMP, AP overshoot and AHP duration for the two models.

**Fig 15:**
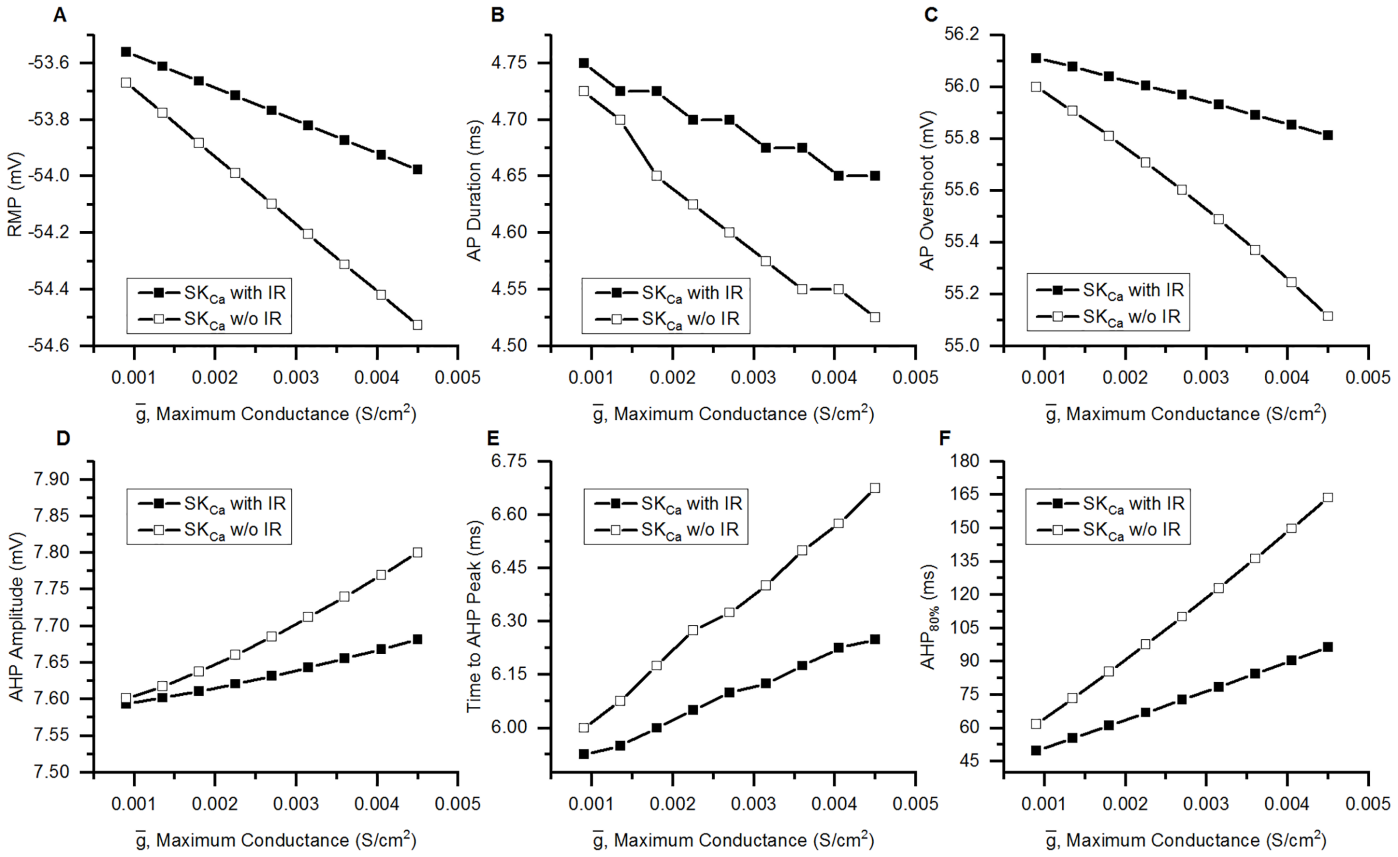
Effects of SK_Ca_ channel inward rectification on AP and AHP properties. Comparison of RMP (A), AP Duration (B), AP overshoot (C), AHP Amplitude (D), Time to AHP Peak (E) and AHP_80%_ (F) in bladder small DRG neuron soma models having SK_Ca_ conductance endowed with inward rectification (SK_Ca_ with IR, filled squares) and without inward rectification (SK_Ca_ w/o IR, hollow squares). AP was generated by a current clamp of amplitude 0.18 nA and duration 15 ms. The $\bar{g}$ was increased from 9*10^−4^ S/cm^2^ to 4.5*10^−3^ S/cm^2^ in steps of 4.5*10^−4^ S/cm^2^. Parameters plotted are explained in the text. Incorporation of inward rectification diminished the effect of SK_Ca_ channel on the AP and AHP parameters.

In order to quantify the effect of SK_Ca_ inward rectification, we compared the 2 soma models: one with inward-rectifying SK_Ca_ (with IR) and other with non-rectifying SK_Ca_ (w/o IR), with reference to the AP and AHP properties viz. (i) RMP: V_m_ just before the start of stimulus, (ii) AP Duration: the spike width at half AP amplitude (measured from RMP) and is the duration between 2 points on an AP when V_m_ reaches half the AP Amplitude (AP Amplitude is the difference in V_m_ between RMP and AP Peak), (iii) AP Overshoot: maximum positive potential above 0 mV during an AP, (iv) AHP Amplitude: the difference between the RMP and minimum potential (AHP peak) reached during an AHP, (v) Time to AHP Peak: time duration between start of AHP and time when V_m_ reaches its minimum value during an AP, (vi) AHP_80%_ is the time duration between start of AHP and time when AHP decays to 80% of AHP Amplitude after AHP Peak. $\bar{g}$ of the SK_Ca_ was raised from the control value of 9*10^−4^ S/cm^2^ to 4.5*10^−3^ S/cm^2^ in steps of 4.5*10^−4^ S/cm^2^ (Fig 15).

The inward rectification reduces the effect of SK_Ca_ channel on AP and AHP parameters. SK_Ca_ inward rectification results in a shorter AP duration and decreased AP overshoot (Fig 15B and 15C). Reduced AHP Amplitude and a shorter time to AHP peak were also observed in model having SK_Ca_ inward rectification (Fig 15D and 15E). A prominent decrease in AHP duration due to inward rectification is evident from AHP_80%_ values (Fig 15F). Hence, the changes in the parameters analysed above due to SK_Ca_ inward rectification were small except for AHP_80%_.

To understand the temporal role of inward rectification (IR) of SK_Ca_ channel on the APs, we analysed the calcium-dependent activation (*o*), the IR parameter (*m*) and the instantaneous conductance (*g*) of the SK_Ca_ channels. *g* is given by the expression:

$$g=\{\begin{array}{ll} \bar{g}*o*m & \text{For}\;{\text{SK}}_{\text{Ca}}\;\text{with}\;\text{IR}\\ \bar{g}*o & \text{For}\;{\text{SK}}_{\text{Ca}}\;\text{w}/\text{o}\;\text{with}\;\text{IR} \end{array}$$ (55)

where $\bar{g}$ is the maximum conductance of the SK_Ca_ channels.

**Fig 16:**
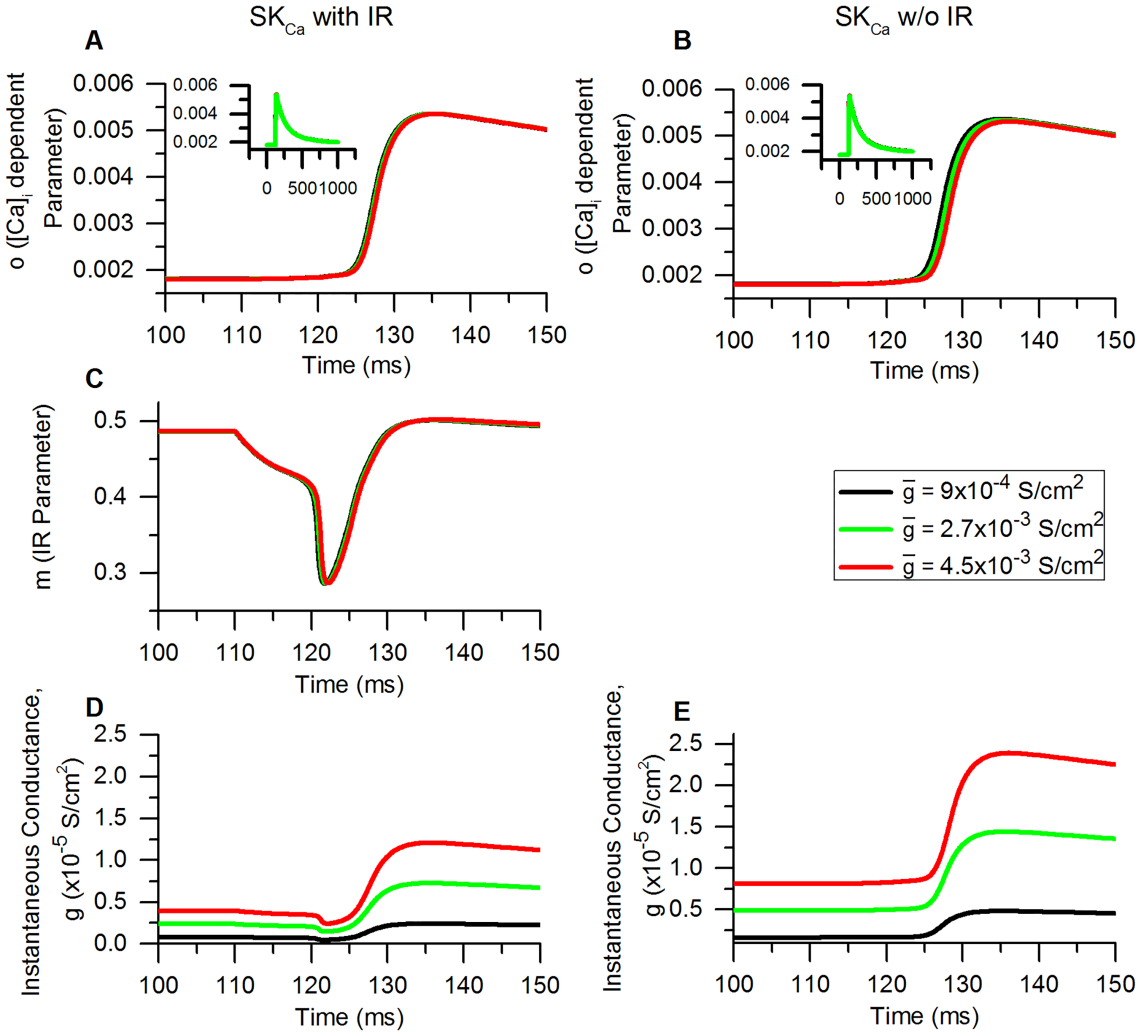
SK_Ca_ conductance changes during an AP in model of SK_Ca_ with IR and SK_Ca_ without IR. The calcium-dependent parameter *o* (A and B), inward rectification (IR) parameter, *m* (C) and the instantaneous conductance, *g* (D and E) of SK_Ca_ with IR (A, C and D) and SK_Ca_ w/o IR (B and E) during an action potential in bladder small DRG neuron model. The 3 colours represent simulations for 3 different maximum conductance ($\bar{g}$) of SK_Ca_ channels: black = 9*10^−4^ S/cm^2^, green = 2.7*10^−3^ S/cm^2^ and red = 4.5*10^−3^ S/cm^2^. Inset in A and B show the variation of *o* for 1000 ms. [Ca]_i_ also follows a similar trend as *o* for both the models. Stimulus: Rectangular current clamp with amplitude of 0.18 nA, duration = 15 ms and delay = 110 ms (Same as in Fig 15). Notice the trough in the *g* for SK_Ca_ with IR (D) which is the result of decrease in *m* during the depolarizing phase of the AP.

Fig 16 shows the results from 2 small DRG neuron models: one having an SK_Ca_ channels with IR (SK_Ca_ with IR) (Fig 16A, 16C and 16D) and the other having non-IR SK_Ca_ channels (SK_Ca_ w/o IR) (Fig 16B and 16E).

The presence of the IR parameter, *m*, reduces the instantaneous conductance, *g* of SK_Ca_ channel during the stimulation duration. *m* which is a function of V_m_ and [Ca]_i_ behaves similarly as the voltage-dependent inactivation parameter (h) of the Hodgkin-Huxley sodium conductance. During the depolarizing phase of the AP, there is a decrease in the value of IR parameter, *m* (Fig 16C) till the peak of the AP (which corresponds to the lowest point in *g* curve) and thus, results in a decrease in the conductance of the channels (Fig 16D). In contrast for DRG neuron model having a non-inward rectifying SK_Ca_ channel (SK_Ca_ w/o IR), *g* follows the same trend as the calcium-dependent activation parameter, *o* (Fig 16B and 16E). We also checked how the variation in the maximum conductance ($\bar{g}$) affects the *g* dynamics of the channel. The 3 colours in Fig 16 represent simulations for 3 different maximum conductances ($\bar{g}$) of SK_Ca_ channels: black = 9*10^−4^ S/cm^2^ (control), green = 2.7*10^−3^ S/cm^2^ and red = 4.5*10^−3^ S/cm^2^. Increasing $\bar{g}$ leads to changes in *o*, *m* and *g* which were amplified version of the trend observed for the respective parameters in control conditions (9*10^−4^ S/cm^2^, black curves) for both the models (SK_Ca_ with IR and SK_Ca_ w/o IR).

We next checked how these changes due to IR translate into changes in repetitive firing in our model.

#### Train of APs: Decrease in rate of failure

**Fig 17:**
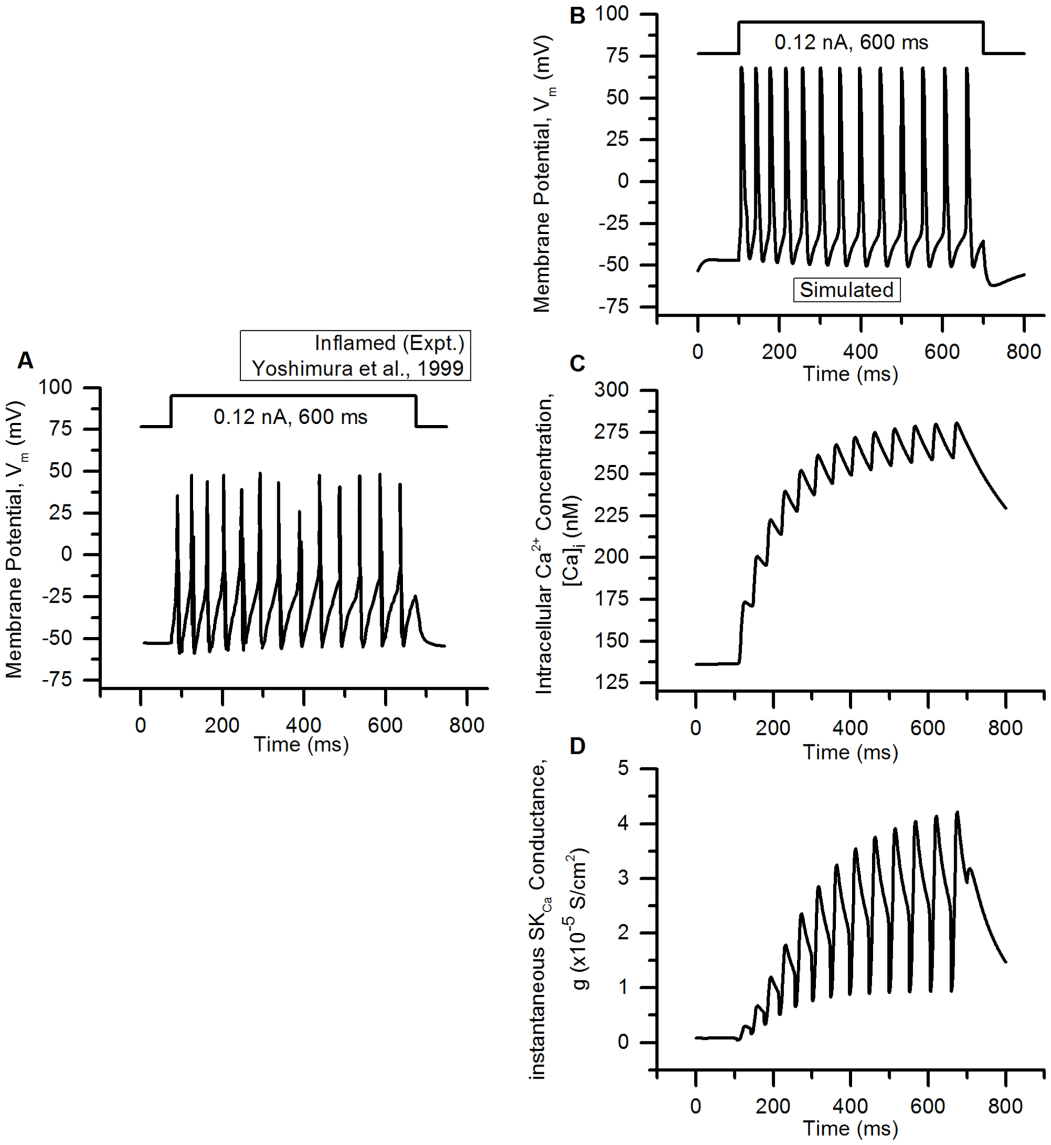
Simulating repetitive firing as seen in bladder small DRG neuron soma during bladder inflammation. (A) The AP firing in the bladder capsaicin-sensitive (or small-diameter) DRG neuron soma in bladder inflammation evoked by application of a long duration current clamp stimulus (0.12 nA, 600 ms) [10]. Stimulus resulted in generation of 12-13 APs in 600 ms (∼ 20 Hz). Compare with Fig 9B in which a long duration stimulus gave only one spike. (B) Simulating the inflammatory firing frequency in model neuron by altering the Na_v_ 1.8, K_A_ and KDR channel maximum conductances. (C) Temporal changes in intracellular calcium concentration,[Ca]_i_ and (D) instantaneous SK_Ca_ conductance, *g* for the firing shown in (B).

In conditions like spinal cord injury and inflammation, bladder small DRG neurons start firing APs in a tonic manner in response to a long duration injected current as opposed to phasic behaviour with a single AP as seen in control neurons [10, 49]. Bladder small DRG neurons of rats treated with cyclophosphamide (an inflammatory agent) fired in a tonic manner (∼12-13 APs for 600 ms stimulus) for long rectangular current clamp stimulus (Fig 17A) whereas the control neurons were phasic in nature and fired just 1 AP for the same stimulus (Fig 9B) [10]. The tonic firing occurs mostly because of changes in the ion channel densities of TTX-S, TTX-R Na^+^, K_A_ and KDR channels [5, 9, 10, 34, 49].

We wished to study the sensitivity of the model spike firing to the inward rectification of SK_Ca_ channel in controlling the firing of bladder DRG neurons in above-mentioned conditions. We implemented the abnormal firing found in bladder small DRG neurons obtained from inflamed bladder by changing the maximum conductances, $\bar{g}$ (which represents ion channel densities) of Na_v_1.8, K_A_ and KDR channels [10, 33, 119]. The modified $\bar{g}$ for these channels were: Na_v_1.8 = 0.06 S/cm^2^, K_A_ = 0.0001 S/cm^2^ and KDR = 0.001 S/cm^2^. A long duration rectangular current clamp of amplitude 0.12 nA for 600 ms as shown in Fig 17B resulted in the generation of tonic firing in the model. As reported in [10], this stimulus was also found to generate tonic firing in bladder small DRG neuron from an inflamed bladder (Fig 17A, 12-13 APs in 600 ms ∼ 20 Hz). Fig 17C and 17D show the temporal changes in [Ca]_i_ concentration and instantaneous SK_Ca_ conductance, *g* respectively. The SK_Ca_ conductance was observed to increase with increasing [Ca]_i_.

**Fig 18:**
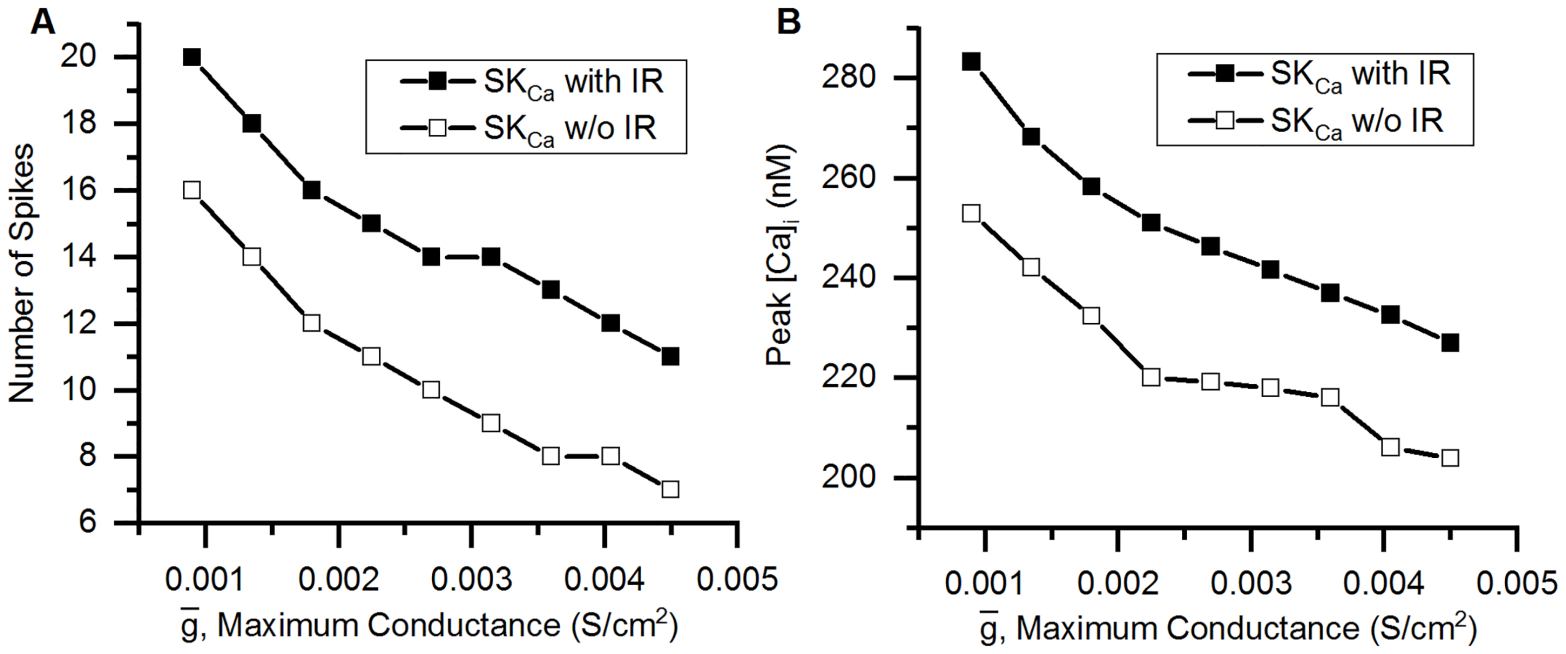
Effect of SK_Ca_ inward rectification on inflammatory firing in modelled neuron. (A) Effect of change in $\bar{g}$ (maximum conductance) of SK_Ca_ on the firing rate of bladder small DRG neuron soma model that has inward rectifying SK_Ca_ channels (SK_Ca_ with IR, filled squares) and in a model having non-inward rectifying SK_Ca_ (SK_Ca_ w/o IR, hollow squares). The $\bar{g}$ was changed between 9*10^−4^ S/cm^2^ and 4.5*10^−3^ S/cm^2^ and a rectangular current clamp of 0.12 nA, 1000 ms was applied for each $\bar{g}$. (B) Corresponding peak [Ca]_i_ attained in the 2 models. The peak [Ca]_i_ decreases with decreasing spiking rate. Inward rectification of SK_Ca_ reduces the failure of spikes.

In order to analyse the effects of SK_Ca_ IR on firing frequency, we used the above described tonic firing model with a slightly longer duration (1000 ms) rectangular current clamp of amplitude 0.12 nA which generated 20 APs (∼ 20 Hz). The $\bar{g}$ value of SK_Ca_ was altered between 9*10^−4^ S/cm^2^ and 4.5*10^−3^ S/cm^2^ in steps of 4.5*10^−4^ S/cm^2^. We measured the change in spike count and the peak [Ca]_i_ reached during the protocol. A spike was counted whenever V_m_ crossed 0 mV from a negative to a positive V_m_. A decrease in number of spikes with increasing conductance was observed for both the models (Fig 18A, SK_Ca_ with IR and SK_Ca_ w/o IR) as the outward current though SK_Ca_ increases. A similar downward trend is seen in the peak [Ca]_i_ attained for both the models with increasing conductance. The outward current via inward rectifying SK_Ca_ is much less compared to SK_Ca_ lacking inward rectification. Thus, the probability of failure of APs is comparatively smaller for the SK_Ca_ with IR model which is also evident from our results (Fig 18A, filled squares).

**Fig 19:**
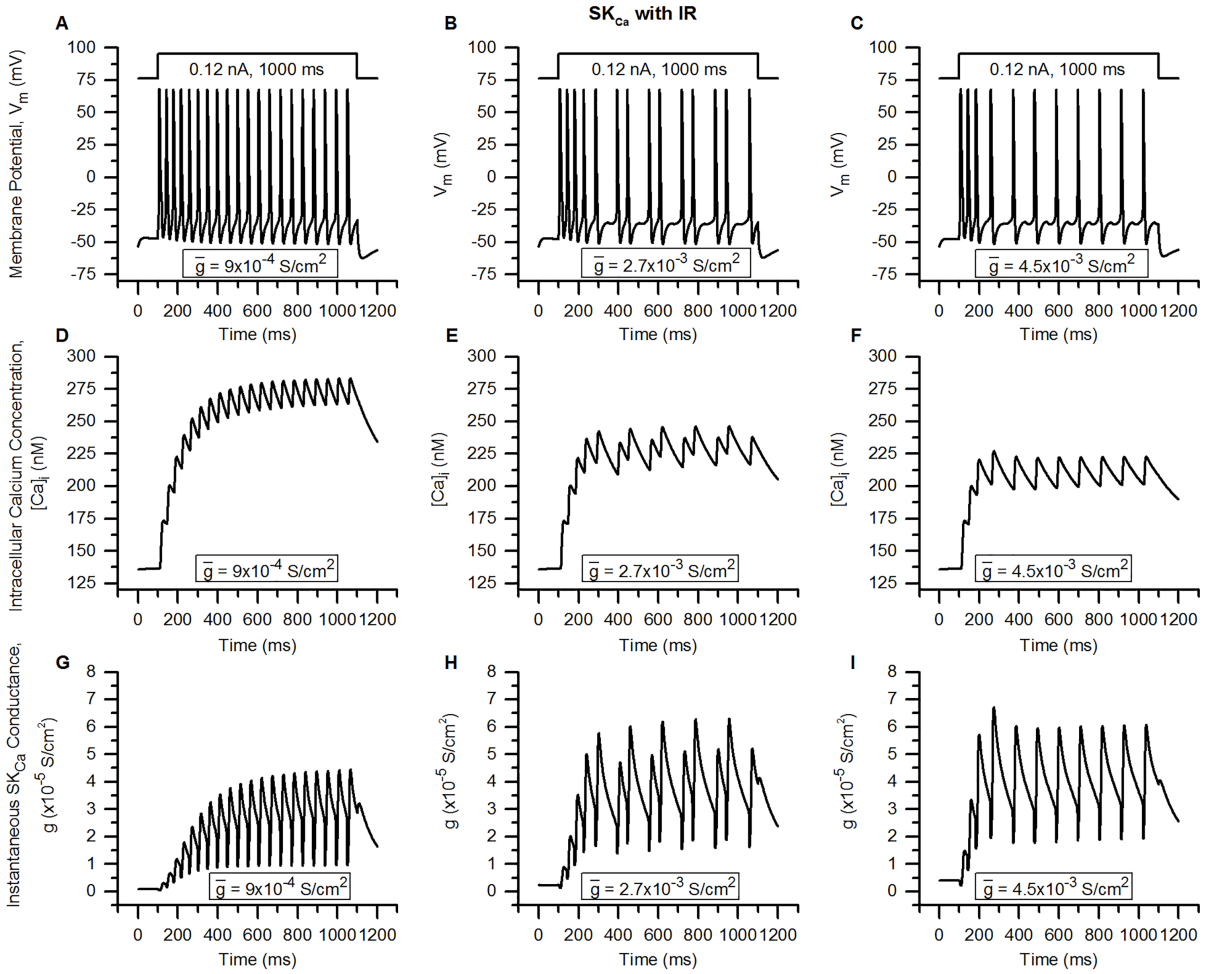
SK_Ca_ with IR: Role of SK_Ca_ inward rectification and SK_Ca_ and maximum conductance on inflammatory repetitive firing. Membrane potential, V_m_ (upper panel), intracellular calcium concentration, [Ca]_i_ (middle panel) and instantaneous SK_Ca_ conductance, *g* (lower panel) recorded against time for three maximum conductances ($\bar{g}$) of the SK_Ca_ channel: 9*10^−4^ S/cm^2^ (control) (A, D, G), 2.7*10^−3^ S/cm^2^ (B, E, H) and 4.5*10^−3^ S/cm^2^ (C, F, I). The neuron model used had SK_Ca_ channels endowed with inward rectification (SK_Ca_ with IR). Stimulus: 1000 ms, 0.12 nA rectangular current clamp.

**Fig 20:**
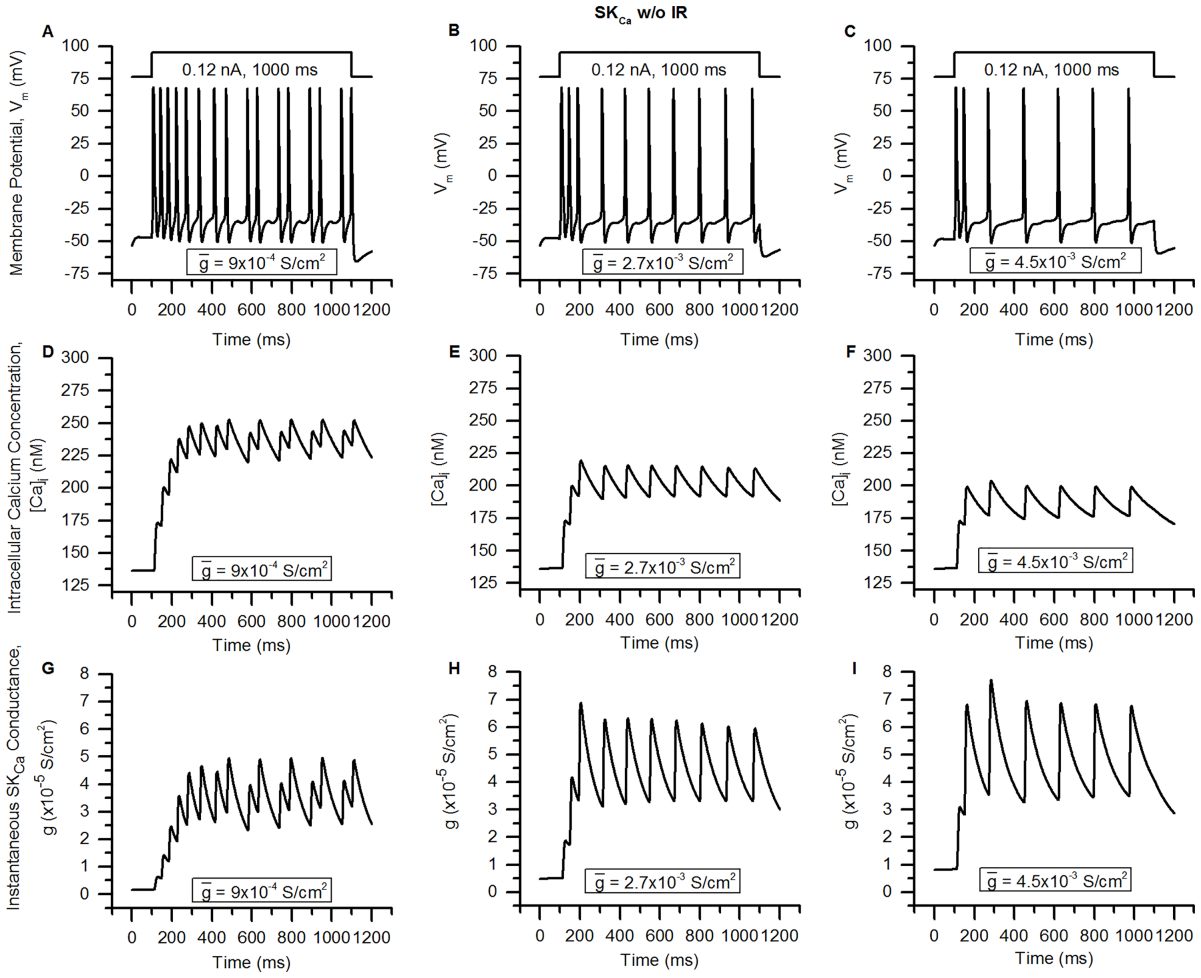
SK_Ca_ w/o IR: Role of SK_Ca_ inward rectification and SK_Ca_ and maximum conductance on inflammatory repetitive firing. Membrane potential, V_m_ (upper panel), intracellular calcium concentration, [Ca]_i_ (middle panel) and instantaneous SK_Ca_ conductance, *g* (lower panel) was recorded against time for three maximum conductances of the SK_Ca_ channel: 9*10^−4^ S/cm^2^ (control) (A, D, G), 2.7*10^−3^ S/cm^2^ (B, E, H) and 4.5*10^−3^ S/cm^2^ (C, F, I). The neuron model used had non-inward rectifying SK_Ca_ channels (SK_Ca_ w/o IR). Stimulus: 1000 ms, 0.12 nA rectangular current clamp.

To understand how IR leads to a decrease in firing, we analysed temporal [Ca]_i_ and instantaneous conductance, *g* changes at three $\bar{g}$ values shown in Fig 18: 9*10^−4^ S/cm^2^ (control), 2.7*10^−3^ S/cm^2^ and 4.5*10^−3^ S/cm^2^ for both the models: SK_Ca_ with IR (Fig 19) and SK_Ca_ w/o IR (Fig 20).

A reduction in number of spikes with increasing $\bar{g}$ was observed for both the models. Elevation in [Ca]_i_ is proportional to the firing rate of the neuron. This increase is also reflected in the instantaneous conductance (*g*) of the SK_Ca_ w/o IR (Fig 20, lower panel), whereas *g* of the SK_Ca_ with IR (Fig 19, lower panel) shows an initial decrease in the instantaneous conductance at the beginning of each spike in the model. This initial reduction in *g* results from the inward rectification parameter in SK_Ca_ model as discussed for Fig 16D.

The simulations described above suggest the importance of inward rectification in SK_Ca_ channels in controlling AP properties and in turn the firing rate of the small DRG neurons. The models lacking SK_Ca_ with IR will overestimate the firing rate of the neurons and thus, inward rectification in SK_Ca_ channels should be incorporated not only in DRG neuron models but also in other excitable/non-excitable cells models.

### Contribution of BK_Ca_ and SK_Ca_ to AHPs

K_Ca_ channels have been shown to affect the AHP phase in sensory neurons [31, 120] and hence, neurons excitability. The AHPs have 3 phases: the fast AHPs (fAHP) which have a fast rise and last for about a few 10’s of milliseconds, medium-duration AHPs (mAHP) which start within a few milliseconds of the AP and lasts for some 100’s of ms and the slow AHPs (sAHP) that can sustain upto 10’s of seconds [32]. The channels underlying different AHPs may be different for central nervous system (CNS) neurons and peripheral sensory neurons. mAHP is modulated by SK_Ca_ channels in CNS neurons [121] while in vagal primary sensory neurons, BK_Ca_ channels are thought to underlie mAHP [120].

**Fig 21:**
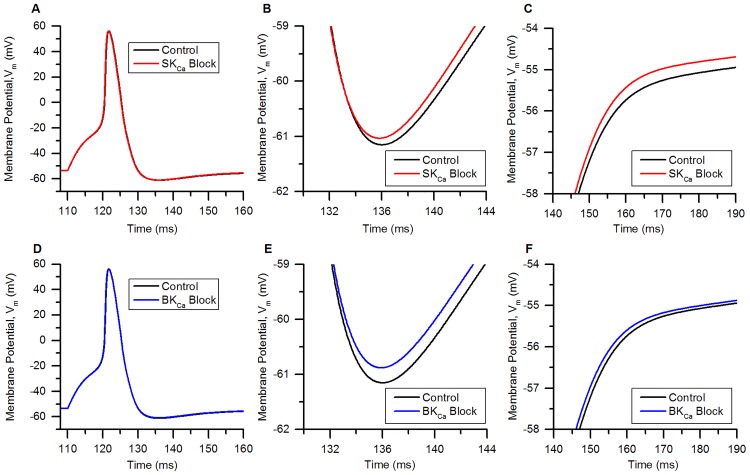
Effect of SK_Ca_ and BK_Ca_ channels on the AHP. (A and D) AP in control conditions (black) and with blocks of SK_Ca_ (red, SK_Ca_ block) and BK_Ca_ (blue, BK_Ca_ block) channels. (B and E) show on an expanded time and voltage scale the effect on the AHP peak. (C and F) also shows on a larger time scale the effect on the AHP phase after the AHP peak during an AP. AP was generated by a current clamp of an amplitude 0.18 nA and 15 ms duration [10].

Here, we conducted a systematic study on the role of BK_Ca_ and SK_Ca_ channels on the AP and AHP properties, and the excitability of bladder small DRG neurons. The conductance of K_Ca_ channels increases with increasing [Ca]_i_ and could play an important role in shaping the APs and the excitability of the neurons. The effect of blocking K_Ca_ channels was minimal on a single AP (generated by rectangular current clamp of 0.18 nA, 15 ms, [10]) due to their small current contributions (Fig 21). As can be seen in Fig 21, the block of BK_Ca_ decreased the AHP Amplitude while SK_Ca_ block resulted in decreasing the AHP duration.

**Fig 22:**
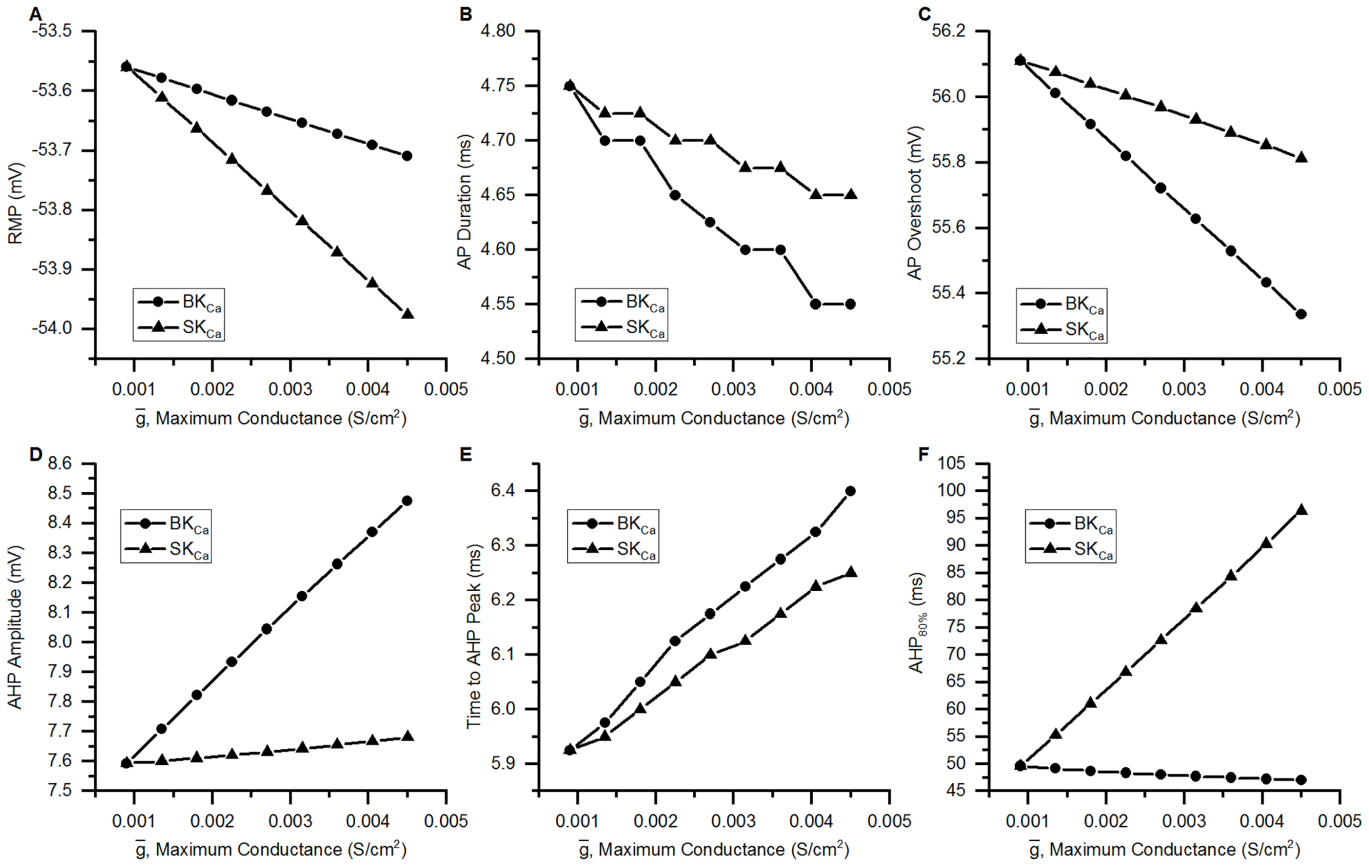
Effect of SK_Ca_ and BK_Ca_ channels on AP and AHP properties. Comparison of RMP (A), AP Duration (B), AP Overshoot (C), AHP Amplitude (D), Time to AHP peak (E) and AHP_80%_ (F) by increasing maximum conductance ($\bar{g}$) of BK_Ca_ (filled circles) and SK_Ca_ (filled triangles) channels in the bladder small DRG neuron soma model. Parameter descriptions are same as earlier in the text.

In order to quantify the changes in the AHP and AP properties, we stepped the $\bar{g}$ of the BK_Ca_ and SK_Ca_ channels from the control value of 9*10^−4^ (for both BK_Ca_ and SK_Ca_) to 4.5*10^−3^ S/cm^2^ in multiples of 4.5*10^−4^ S/cm^2^. As expected from the blocking studies, an elevated BK_Ca_ conductance resulted in increase in AHP Amplitude (Fig 22D) whereas elevated SK_Ca_ conductance led to a slower decay of the AHP (Fig 22F).

BK_Ca_ conductance increase resulted in larger AHP amplitude (Fig 22D) than SK_Ca_. Hence, BK_Ca_ may contribute to the fAHP. The change in AHP_80%_ is larger for a change in SK_Ca_ conductance than for the same change in BK_Ca_ conductance. The greater sensitivity to SK_Ca_ shows that it may regulate the later part of the AHP and may underlie medium-duration AHP (mAHP, which decay within few 100’s of ms) and the slow AHP (lasting a few seconds) in bladder DRG neurons.

### SK_Ca_ is more potent in reducing firing rate than BK_Ca_

In order to test the sensitivity of AP firing to the K_Ca_ channels, we altered the conductance of channels individually in the range of 9*10^−4^ (control) to 4.5*10^−3^ S/cm^2^ to a model that shows inflammatory repetitive firing for a long duration rectangular current clamp of amplitude 0.12 nA and 1000 ms duration, as used in Fig 18. We recorded the number of spikes generated by the model and peak [Ca]_i_. Spikes were counted whenever V_m_ crossed 0 mV from a negative to a positive V_m_.

**Fig 23:**
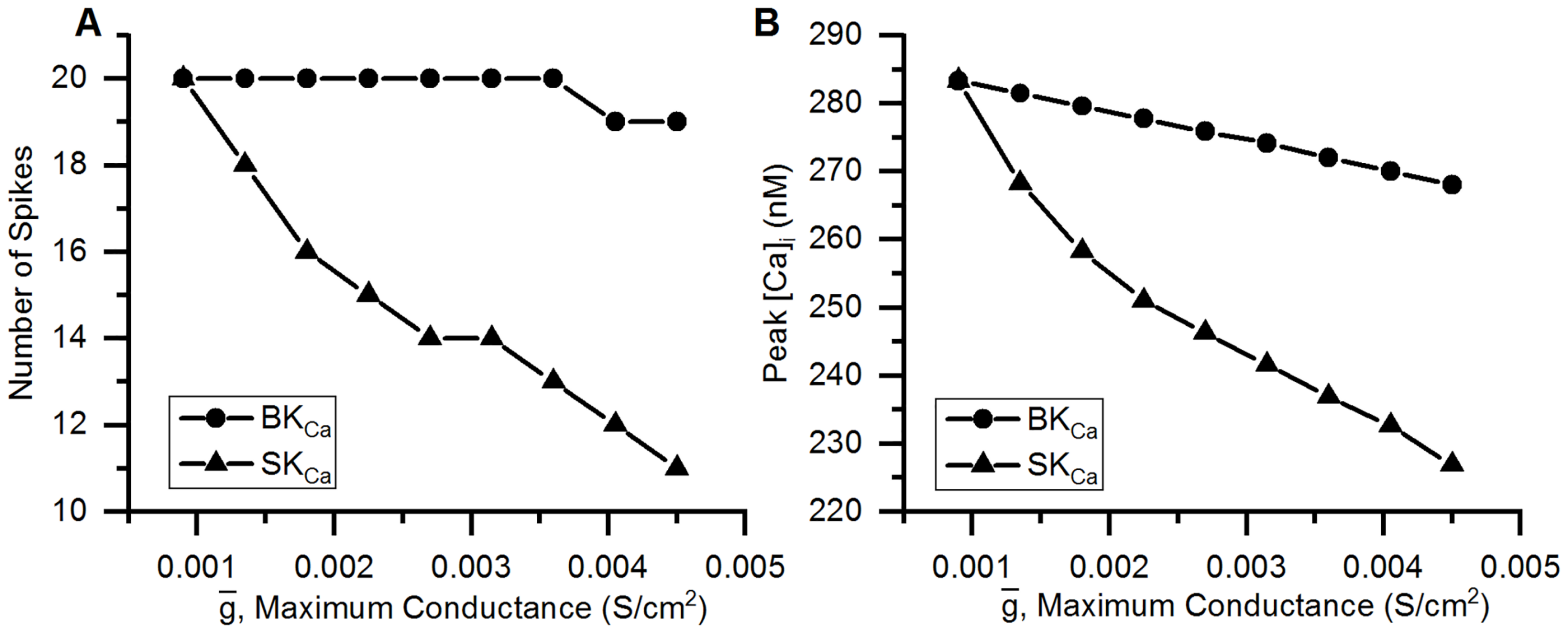
Effect of changes in BK_Ca_ and SK_Ca_ maximum conductance on the train of APs and [Ca]_i_. The maximum conductance $\bar{g}$ of BK_Ca_ and SK_Ca_ channels were changed between of 9*10^−4^ (control value) to 4.5*10^−3^ S/cm^2^ for both SK_Ca_ and BK_Ca_) individually and the model was stimulated with 1000 ms rectangular current clamp of 0.12 nA amplitude. The number of spikes (A) and peak intracellular Ca^2+^ concentration, [Ca]_i_ (B) were recorded at every increment of BK_Ca_ (filled circles) and SK_Ca_ (filled triangles) maximum conductance.

The graphs in Fig 23A show that the SK_Ca_ channel is more likely to change the firing rate than the BK_Ca_ channel when channel conductance is elevated. A decremental trend is seen in maximum [Ca]_i_ (Fig 23B). The decrement is greater for enhanced SK_Ca_ channel conductance compared to BK_Ca_ conductance. This parallels the reduction in the number of APs recorded. It can be inferred from Fig 23B that [Ca]_i_ is more sensitive to SK_Ca_ than to BK_Ca_ at raised conductance values. Thus, while both SK_Ca_ and BK_Ca_ channels affect the firing frequency of the bladder DRG neurons, SK_Ca_ is more effective than BK_Ca_ in this respect. A reason for this is the strong effect of SK_Ca_ on the slow AHP (See Fig 22F for AHP_80%_). AHP_80%_, which indicates the recovery time from AHP towards RMP, is relatively higher at larger conductances for SK_Ca_ channel than BK_Ca_ channels. Hence, a larger depolarization is required to bring V_*m*_ from slow AHP potential to the AP threshold. This will reduce the firing frequency of the neuron.

## Discussion

The computational model presented here describes a comprehensive electrophysiological model of the urinary bladder small DRG neurons validated against experimental data for these neurons. The model includes all the major known plasma membrane mechanisms viz. ion channels, Ca^2+^ pump, Na^+^/Ca^2+^ exchanger as well as all essential components of Ca^2+^ dynamics, namely cytoplasmic diffusion and buffering, endoplasmic reticulum (ER) mechanisms and mitochondrial mechanisms. Hence, our model represents a physiologically realistic model constrained by available biophysical data.

Using our elaborate, validated model of bladder small DRG neuron soma, we addressed certain outstanding questions regarding the factors that govern the functioning of these neurons and showed the following: (1) the inward rectifying property of SK_Ca_ channels increases the excitability of bladder small DRG neurons, (2) BK_Ca_ may contribute chiefly to the fAHP of the spike while SK_Ca_ may contribute to chiefly to the mAHP and sAHP, (3) SK_Ca_ channels are more potent in suppressing AP firing than BK_Ca_ channels, (4) the slow K_A_ currents are composed of 2 inactivation components: a fast component and a slower component which in turn could be the result of 2 different molecular constituents of the channel. We discuss below the implications of these findings for various facets of bladder small DRG neuron functioning and, on a broader level, for urinary bladder function.

### Inward rectification of SK_Ca_ channel

The model of SK_Ca_ channel presented here incorporates both inward rectification and [Ca]_i_-dependence. Our working hypothesis was that since rectification gives rise to smaller outward currents over the relevant range of membrane voltage [27, 28], incorporation of rectification should result in an increase of excitability of the bladder small DRG soma. Our simulations show that SK_Ca_ channels have marked effects on the electrical activity of the bladder small DRG neuron. We show that inward rectification of SK_Ca_ channels can regulate the afterhyperpolarization, RMP and AP properties of these neurons. The DRG neuron model endowed with SK_Ca_ inward rectification exhibited a higher firing rate compared to the model in which SK_Ca_ was endowed just with [Ca]_i_-dependence (Fig 18A). This is expected as the reduced outward current for potentials more depolarized than the Nernst potential of K^+^ prevents the generation of a larger AHP (Fig 14B and 14E) and leads to greater excitability. We propound therefore that, SK_Ca_ channel rectification may play a significant part in shaping and regulating the flow of the electrical sensory signals from the bladder, not only in the DRG soma but also at the DRG neuron terminals in the spinal cord where SK_Ca_ channels could be involved along with Ca^2+^ channels in regulating neurotransmitter release [87, 89]. Another site at which SK_Ca_ channels could play a role in regulating the release of neurotransmitter is the DRG neuron soma since these somata have also been shown to release such neurotransmitters as adenosine triphosphate (ATP) and glutamate which could be involved in intercellular communication between neighbouring somata via the satellite glial cells surrounding them [15].

The presence of rectification has been shown in recombinant cell lines expressing SK_Ca_ channels such as in HEK-293 [24, 25] and *Xenopus* oocytes [26, 28] but rectification in endogenous DRG neurons is not yet studied. Even though the intracellular milieu including the Ca^2+^ release and uptake mechanisms might be different in recombinant cells as compared to DRG neurons, the evidence that rectification is an intrinsic property of the SK_Ca_ channel [26] suggests that this feature might also be present in DRG neurons. Rectification is caused by the presence of charged amino acids in the transmembrane domain S6 of the SK_Ca_ channel [26]. Jenkins et al. [122] found that serine-507 and alanine-532 residues in the inner pore region when substituted by threonine and valine, respectively reduced the rectification in human K_Ca_ 2.3 (hSK3) channels. In addition, in view of the finding that SK_Ca_ inward rectification is an outcome of block by intracellular divalent cations like Ba^2+^ and Mg^2+^ [24, 25], it is highly likely that it is also found in the native DRG neuron including the bladder DRG neurons. The effect of this property on the electrical activity of neurons therefore merits considerable attention.

### Contribution of BK_Ca_ and SK_Ca_ to AHP

The role of different K_Ca_ (BK_Ca_, SK_Ca_ and IK_Ca_) channels in shaping the AP is not studied in detail in the bladder DRG neurons while there are numerous studies on other somatic and non-bladder DRG (sensory) neurons such as the cutaneous DRG neurons and vagal sensory neurons [54, 120, 123]. Pharmacological studies using blockers and openers of BK_Ca_, SK_Ca_ and IK_Ca_ channels in bladder small DRG neurons have been reported to change their electrical excitability [30, 52]. Hougaard et al. [30] tested the effects of SK_Ca_ blocker (apamin) and opener/positive modulator (1-EBIO and NS4591) on bladder small DRG neurons. The SK_Ca_ channel positive modulator NS4591 at concentration of 1 *μ*M hyperpolarized the RMP. In our simulation, application of channel opener was mimicked by augmenting the conductance of the SK_Ca_ channel which also resulted in hyperpolarized RMP (See Fig 22A). Apamin decreased the amplitude of the AHP recorded after one AP [30], an effect similar to that seen in simulations by removing SK_Ca_ channels from the model (Fig 21B), the change being very small (∼0.1 mV). Similarly, minimal increase (<0.1 mV) in AHP amplitude with increasing SK_Ca_ conductance in our simulation (Fig 22D) is in line with the experimental observation that AHP amplitude does not change on applying 1-EBIO and NS4591 [30]. The application of these drugs (NS4591 and 1-EBIO) reduced the number of APs recorded during a 500 ms long current clamp stimulus from 8 to 2 APs. This effect may be correlated with our studies on the effect of SK_Ca_ on a train of APs where an elevated SK_Ca_ conductance decreases firing rate in the neuron (See Fig 23A).

Unlike our observations of an enhanced AHP decay time (AHP_80%_) with raised SK_Ca_ conductance (Fig 22F, Filled triangles and Fig 15F, SK_Ca_ with IR), Hougaard et al. [30] did not observe significant change in AHP decay time on application of SK_Ca_ openers in bladder small DRG neurons. The difference in AHP decay time in experiments and our simulations of DRG model with SK_Ca_ endowed IR (Fig 22F, Filled triangles) can arise because of the hyperpolarized holding potentials (−70 mV, −62 mV) used in the experimental work before applying blockers and openers while the RMP is close to −50 mV. The RMP reported for L6-S1 spinal level rat bladder small DRG neuron is generally between −48 to −53.5 mV [5, 10, 34] whereas the hyperpolarized holding potential used by [30] could lead to activation of channels currents such as slow K_A_, Na_v_1.9 and HCN channels. These channels have activation parameters that operate at more hyperpolarized potentials compared to those of other channels (See Fig 3A for n_∞_ of slow K_A_ and Figs Aa and Ga in S2 Text for m_∞_ of Na_v_1.9 and HCN). These channels could affect the amplitude of the AHP as well as recovery from it to the RMP. Some neurons tested by Hougaard et al., [30] also fired tonically to a 500 ms suprathreshold current clamp. Bladder small DRG neurons usually fire just a single AP for such a stimulus (Fig 9B) while the medium-diameter bladder DRG neurons show tonic firing with many spikes [3, 34, 49]. A BK_Ca_ channel blocker, A-272651 increased the AP duration in L6-S1 spinal level capsaicin-sensitive small DRG neurons from rats [52], some of which supply the bladder [3, 85, 86]. The effect of BK_Ca_ channel block in our model did not result in an appreciable increase in AP duration (Fig 21D). A probable reason for this inconsistency could be that the neurons tested represent a different subpopulation of bladder small DRG neurons. Our model reproduced the behaviour obtained by BK_Ca_ block of Isolectin B4 (IB4)-negative cutaneous small DRG neurons (Fig 8B of [54]). Compared to IB4-positive neurons, IB4-negative neurons have fewer Na_v_1.9 and K_A_ channels and have a less negative RMP [35, 124, 125]. Bladder small DRG neurons, most of which are IB4-negative (∼75%, [3]) have a depolarized RMP (−53.5 mV, [10]) than the RMP of small DRG neurons studied in [52] (< −60 mV). The expression of Na_v_1.9 and K_A_ channels also is far less than that in other non-bladder DRG neurons [35, 51]. Moreover, bladder small DRG neurons generate a single AP for a long duration rectangular clamp similar to cutaneous IB4-negative small DRG neurons reported by [54] whereas neurons investigated by [52] gave rise to more than one AP for similar stimulus as also seen for IB4-positive cutaneous DRG neurons [54]. The firing frequency for IB4-positive cutaneous DRG neurons increased by using BK_Ca_ blocker while it did not for IB4-negative DRG neurons. In the light of these observations, it could be reasoned that neurons in Shieh et al. [52] are IB4-positive which probably have higher BK_Ca_ expression than IB4-negative bladder small DRG neurons. Taking this into consideration, our BK_Ca_ channel and bladder small DRG neuron models possibly represents model of IB4-negative bladder small DRG neurons. The two categories IB4-positive and IB4-negative neurons could be explored further through simulations by altering the conductances for Na_v_1.9, K_A_ and BK_Ca_ channels of our model as per the above observations and could also be explored experimentally by studying the ion channel expressions and electrophysiological properties of IB4-positive and IB4-negative bladder small DRG neurons. Although, IB4-positive bladder small DRG neurons represent smaller subpopulation (∼ 15-20%) [90] compared to IB4-negative, both seem to be significant in nociception [3, 126, 127]. Electrophysiological and ion channel expression differences in these two subpopulations of bladder small DRG neurons could be important regulators of excitability as shown in non-specific small DRG neurons [125, 128], and also merit further experimental exploration.

The precise genesis of the AHP in the bladder small DRG neuron and AHP effects on the excitability of bladder small DRG neurons [30] is not clear. In rabbit vagal sensory ganglion neurons, fAHPs generally last a few ms (∼ 30 ms) and were Ca^2+^-independent, mAHPs could last for 300 ms while sAHP rise in about 100 ms and can last or 2-15 s [120]. Both mAHPs and sAHPs were Ca^2+^-dependent. In enteric ganglia neurons, BK_Ca_ contributes to the fast AHP [17]. Some differences have been reported for the role of K_Ca_ channels in central neurons and peripheral sensory neurons. mAHPs were blocked by picomolar concentrations of apamin (SK_Ca_ blocker) in CNS neurons [32]. Apamin, even at millimolar levels did not block the mAHPs in vagal sensory neurons [31], in which and BK_Ca_ channels were predicted to underlie mAHP. SK_Ca_ channels, in contrast, were shown to contribute to sAHPs [31, 121].

Based on the above lines of evidence and to clarify the contributions of K_Ca_ channels to different AHPs, we hypothesized that the fAHPs receive contributions chiefly from the BK_Ca_ conductance while SK_Ca_ channels contribute to mAHPs and sAHPs. It was found that BK_Ca_ can regulate the fAHP as they contribute to the fast rise of AHP (< 3 ms) and the AHP amplitude is augmented on increasing the BK_Ca_ conductance. SK_Ca_ channels contribute more strongly to the AHP phase after the fAHP peak, and hence may underlie the mAHP and sAHP.

### Effect of BK_Ca_ and SK_Ca_ on train of APs

Bladder small DRG neurons obtained from rats with bladder inflammation and spinal cord injury were found to be more excitable than corresponding neurons from controls [1, 5, 9, 10, 35, 49]. DRG neuron somata in the former undergo hypertrophy [3] and exhibit plasticity in ion channel expression such as for TTX-S, Na_v_1.8, slow K_A_ and KDR channels [5, 9, 10, 34] leading to lower AP threshold and thus, greater excitability. Given these observations, we thought to test the effects of BK_Ca_ and SK_Ca_ channels on regulating the firing rate of the bladder small DRG neurons. It was found that increasing the conductance of BK_Ca_ and SK_Ca_ channels, which would mimic application of pharmacological channel openers to bladder small DRG neurons, reduced the spiking induced by changing channel densities of Na_v_1.8, slow K_A_ and KDR channels. We replicated the firing frequency (∼20 Hz) as seen in bladder small DRG neuron obtained from rats with inflammatory cystitis [10] (Fig 17A). Enhancement of SK_Ca_ conductance lead to a larger reduction in spiking than commensurate enhancement of BK_Ca_ conductance, suggesting that SK_Ca_ activators could be more effective in controlling hyperexcitability of bladder small DRG neurons than the BK_Ca_ activators.

### Slow K_A_ current has 2 components

Slow K_A_ currents are found in bladder small DRG neurons while the medium diameter bladder DRG neurons express fast K_A_ currents [7, 49]. Slow K_A_ currents appear to be important currents in bladder small DRG neurons, as a drop in their current density in spinal transected rats [34] as well as in rats with chronic bladder inflammation [10, 36] has been reported to augment the excitability of the neurons.

Though several studies have been carried out to investigate the molecular components of these slow K_A_ currents [34–36], the contribution of different K_v_ components to the slow K_A_ current is not clear. We showed by optimization of our slow K_A_ current model that the inactivating phase of experimental slow K_A_ currents under voltage clamp in bladder small DRG neurons were best fit by a biexponential decay, comprising of a fast-decaying exponential (*τ*_*h*,*fast*_) and a slow-decaying exponential (*τ*_*h*,*slow*_). This could indicate the existence of at least 2 current components which in turn could mean 2 separate molecular components.

The molecular identity of the slow K_A_ channels expressed in these neurons is still not clear. K_A_ channels in some non-bladder DRG neurons can form both homomers and heteromers [129]. In non-bladder DRG neurons, expression of K_v_1.1, 1.2, 1.4, 4.1 and 4.3 K_A_ channel forming subunits has been reported [35, 36, 129]. Yunoki et al. [35] showed that phrixotoxin 2, a K_v_4 channel blocker was able to block the K_A_ currents in IB4 (isolectin B4)-positive non-peptidergic somatic DRG neurons and not in the IB4-negative peptidergic bladder DRG neurons. From these data we conclude that K_v_4 subunits are highly unlikely to contribute to bladder slow K_A_ currents. Moreover, the steady state half-inactivation parameters of K_v_4.1, 4.2 and 4.3 subunits (−44, −44 and −32 mV, respectively) expressed in Chinese hamster ovary cells [35] are more depolarized than that of bladder slow K_A_ channel (−74.2 mV) [10]. Hayashi et al. [36] reported a preferential expression of K_v_1.4 *α* subunits in bladder small DRG neurons. Takahashi et al. [34] and Hayashi et al., [36] also suggested that K_v_1.1 or K_v_1.2 could form heteromers with K_v_1.4 to form slow K_A_ currents in bladder small DRG neurons.

K_v_1 subunits expressed in HEK-293 cell lines showed different current inactivation profiles [130]. The K_v_1.2 channels exhibited a slow inactivation with an inactivation time constant close to that of the slow component modelled in our study (200-800 ms, *τ*_*h*,*slow*_ Fig 3C) while the K_v_1.1 channel expressing cells do not seem to inactivate discernibly within 1000 ms. K_v_1.4 expressing HEK cells showed an inactivation which is much faster than K_v_1.2 and is close to the value for the fast component of the K_A_ channel model in our study (25-100 ms, *τ*_*h*,*fast*_, Fig 3C). The similarity of values of inactivation time constants of K_v_1.2 and K_v_1.4 to those of our model suggests that the subunits most likely to form the slow K_A_ channels found in the bladder small DRG neurons are heteromers of K_v_1.2 and K_v_1.4.

Another possible reason for two different phases in the inactivation of slow K_A_ currents of bladder small DRG neurons can be the existence of dual inactivation mechanism as mediated by the same molecular unit comprising the slow K_A_ current. K_v_1.4 subunits could show such phenomena [131], displaying two inactivation mechanisms: (i) N-type, a fast inactivation (50-350 ms) which is caused by binding and blocking of channel pore with N-terminal ball, and (ii) C-type, a slower inactivation (2-3 s) which is believed to be caused by intracellular and conformational changes of the channel [131]. Bett et al. [131] showed that the coupling between N-type and C-type inactivation in K_v_1.4 channels can explain experimentally recorded K_v_1.4 currents in inactivation time constants with intermediate values of fast and slow inactivation. A similar coupling could also explain the biexponential decay found in the slow K_A_ currents of the bladder DRG neuron. Considering all the arguments above, a more detailed molecular analysis of slow K_A_ channels of bladder neurons will shed light on the unresolved questions.

### Limitations and avenues for further exploration

Contributions HCN, KCNQ/M and slow K_A_ channels to different AHPs were not studied as we only focussed on the contribution of BK_Ca_ and SK_Ca_ channels. Moreover, the expression and the contribution of HCN and KCNQ/M channels in the bladder small DRG neurons comparatively smaller [8, 42] than other currents and may have minor effects on AHP.

The effect of neurotransmitters such as ATP and certain channels such as TRP (transient receptor potential) vanilloid 1 and ASIC (acid-sensing) channels were not added to the model. These mechanisms are activated by specific stimuli (such as capsaicin and abnormal pH) which assume greater importance in pathological conditions. Likewise, the effect of the satellite glial cells (SGCs) on the soma were omitted for similar reasons. On the morphological level, a complete model with axon and its sensory terminals in the bladder (including various receptors e.g. for noxious stimuli) can provide a finer-grained understanding of sensory information transmission from the bladder. Few studies have reported effects of the geometry of the T-junction of the DRG neuron and ion channel densities in stem axon and soma on the electrical activity [11–13]. Taking these considerations into account, conduction of sensory information can be understood in greater depth. We have commenced work on several of these fronts such as modelling the TRPV1 channels [39] and SGC interactions with the DRG neuron soma [38], and they will be taken up in future studies.

### Conclusions

Our detailed and biophysically constrained computational model of bladder small DRG neuron soma presented here was able to reproduce experimentally observed signals such as the action potential and cytoplasmic Ca^2+^ transients. By exercising our model in appropriate ways we were able to (i) corroborate our hypothesis concerning the effect of inwardly rectifying SK_Ca_ channels on neuronal excitability; (ii) gain several insights into the roles of BK_Ca_ and SK_Ca_ channels in bladder small DRG neuron such as the contributions of these channels to the genesis of different types of AHP and the relative efficacies of BK_Ca_ and SK_Ca_ channels in the regulation of repetitive firing in these neurons, thus casting light on hitherto unresolved biological questions. In addition, we suggest a difference in expression of BK_Ca_ in IB4-negative and IB4-positive populations of bladder small DRG neurons which could determine the repetitive firing similar to that seen in cutaneous small DRG neurons [54]. We also suggest that the slow K_A_ currents could be made up of 2 components; a fast inactivating and a slow inactivating current which could either be a result of heteromeric K_A_ channel formed by K_v_1.4 and K_v_1.2 subunits (both found in bladder small DRG neurons, [34, 36] or a result of interaction between 2 different inactivation states exhibited by K_v_1.4 subunits (C-type and N-type). These insights can be tested experimentally, for instance by the use of drugs to remove the inward rectification of the SK_Ca_ channel. The consequent reduction in spiking frequency may in some conditions hold promise as a potential intervention such as in mitigating hyperexcitability of bladder small DRG neurons, thus having implications for certain types of pathology. Our work thus provides heuristic predictions that can lead to a deeper understanding of bladder small DRG neuron function, its role in the regulation of bladder physiology and its possible involvement in pathophysiology.

## Supporting information

S1 Fig Schematic of the bladder small DRG neuron soma model. The model consists of 22 membrane mechanisms including Na^+^, K^+^, Ca^2+^, Cl^-^, and some non-specific ion channels such as TRPM8, HCN and passive channels as well as pumps such as Na^+^/K^+^-ATPase Pump, PMCA pump and Exchanger (NCX). The description of mechanisms is given in Methods and S2 Text. The soma is intracellularly divided into 12 concentric shells which facilitate diffusion of Ca^2+^ and IP3 (inositol 1,4,5 triphosphate) as well as in creating separate Ca^2+^ pools for different mechanisms.
(TIF)

S2 Fig Ca^2+^ dynamics in an intracellular shell in bladder small DRG neuron soma model. In each shell, 81% of the total volume is cytoplasm, 12% is occupied by endoplasmic reticulum (ER) and mitochondria make 7% of the total volume. Ca^2+^ and IP3 can diffuse from one shell to the another. Ca^2+^ coming in the cytoplasm to a shell via diffusion is buffered immediately. The ER has 4 mechanisms: SERCA is responsible for replenishing the ER Ca^2+^ concentration [Ca]_ER_ caused by release from ryanodine receptors (RYR) which are activated by increase in cytoplasmic Ca^2+^; IP3R (IP3 receptors) which open on activation by IP3 molecules and Ca^2+^ ions; and the ER leak channel helps to maintain a steady resting state [Ca]_ER_. Ca^2+^ ions entering the ER as well as mitochondria is also buffered. Mitochondrial calcium entry occurs via mitochondrial uniporter (MCU) and calcium is released by mitochondrial sodium-calcium exchanger (MNCX). See Ca^2+^ dynamics in Methods.
(TIF)

S3 Fig CaCC and SOCC coupling with endoplasmic reticulum in outermost shell in bladder small DRG neuron model. The Ca^2+^-activated Cl^-^s (CaCCs) are activated both by membrane potential and intracellular Ca^2+^. They are more potently gated by IP3R Ca^2+^ release in the outermost shell than by Ca^2+^ influx from voltage-gated Ca^2+^ channels on the membrane. The store-operated Ca^2+^ channels (SOCCs) are activated when there is a depletion of Ca^2+^ in the ER. The Orai1 and STIM1 proteins are responsible for store-operated Ca^2+^ entry in small DRG neurons. See S2 Text for more details.
(TIF)

S1 Text General description of the modelling methods. (PDF)

S2 Text Additional membrane mechanisms in the model. (PDF)

S1 Table Table for ionic mechanism parameters. (PDF)

## References

[1] Yoshimura N, de Groat WC. Neural control of the lower urinary tract. International journal of urology. 1997;4(2):111–125. 10.1111/j.1442-2042.1997.tb00156.x 9179682

[2] de Groat WC. A neurologic basis for the overactive bladder. Urology. 1997;50(6A Suppl):36–52; discussion 53–6. 10.1016/S0090-4295(97)00587-6 9426749

[3] de Groat WC, Yoshimura N. Afferent nerve regulation of bladder function in health and disease In: Sensory Nerves. Springer; 2009 p. 91–138. 10.1007/978-3-540-79090-7_4 PMC3383010 19655106

[4] de Groat WC, Yoshimura N. Plasticity in reflex pathways to the lower urinary tract following spinal cord injury. Experimental neurology. 2012;235(1):123–132. 10.1016/j.expneurol.2011.05.003 21596038 PMC3580860

[5] Kadekawa K, Majima T, Shimizu T, Wada N, de Groat WC, Kanai AJ, et al The role of capsaicin-sensitive C-fiber afferent pathways in the control of micturition in spinal-intact and spinal cord-injured mice. American Journal of Physiology-Renal Physiology. 2017;313(3):F796–F804. 10.1152/ajprenal.00097.2017 28637786 PMC5625111

[6] Palmer CJ, Choi JM. Pathophysiology of Overactive Bladder: Current Understanding. Current Bladder Dysfunction Reports. 2017;12(1):74–79. 10.1007/s11884-017-0402-y

[7] Yoshimura N, White G, Weight FF, de Groat WC. Different types of Na+ and A-type K+ currents in dorsal root ganglion neurones innervating the rat urinary bladder. The Journal of physiology. 1996;494(Pt 1):1–16. 10.1113/jphysiol.1996.sp021471 8814602 PMC1160610

[8] Masuda N, Hayashi Y, Matsuyoshi H, Chancellor MB, de Groat WC, Yoshimura N. Characterization of hyperpolarization-activated current (*I*_*h*_) in dorsal root ganglion neurons innervating rat urinary bladder. Brain research. 2006;1096(1):40–52. 10.1016/j.brainres.2006.04.085 16765328

[9] Yoshimura N, Groat WC. Plasticity of Na+ channels in afferent neurones innervating rat urinary bladder following spinal cord injury. The Journal of Physiology. 1997;503(2):269–276. 10.1111/j.1469-7793.1997.269bh.x 9306271 PMC1159861

[10] Yoshimura N, de Groat WC. Increased excitability of afferent neurons innervating rat urinary bladder after chronic bladder inflammation. The Journal of neuroscience. 1999;19(11):4644–4653. 10.1523/JNEUROSCI.19-11-04644.1999 10341262 PMC6782608

[11] Gemes G, Koopmeiners A, Rigaud M, Lirk P, Sapunar D, Bangaru ML, et al Failure of action potential propagation in sensory neurons: mechanisms and loss of afferent filtering in C-type units after painful nerve injury. The Journal of physiology. 2013;591(4):1111–1131. 10.1113/jphysiol.2012.242750 23148321 PMC3591718

[12] Du X, Hao H, Gigout S, Huang D, Yang Y, Li L, et al Control of somatic membrane potential in nociceptive neurons and its implications for peripheral nociceptive transmission. PAIN^®^. 2014;155(11):2306–2322. 25168672 10.1016/j.pain.2014.08.025 PMC4247381

[13] Sundt D, Gamper N, Jaffe DB. Spike propagation through the dorsal root ganglia in an unmyelinated sensory neuron: a modeling study. Journal of neurophysiology. 2015;114(6):3140–3153. 10.1152/jn.00226.2015 26334005 PMC4686302

[14] Wall P, Devor M. Sensory afferent impulses originate from dorsal root ganglia as well as from the periphery in normal and nerve injured rats. Pain. 1983;17(4):321–339. 10.1016/0304-3959(83)90164-1 6664680

[15] Hanani M. Role of satellite glial cells in gastrointestinal pain. Frontiers in cellular neuroscience. 2015;9 10.3389/fncel.2015.00412 26528140 PMC4602093

[16] Koopmeiners AS, Mueller S, Kramer J, Hogan QH. Effect of electrical field stimulation on dorsal root ganglion neuronal function. Neuromodulation: Technology at the Neural Interface. 2013;16(4):304–311. 10.1111/ner.12028 23421796

[17] Chambers JD, Bornstein JC, Gwynne RM, Koussoulas K, Thomas EA. A detailed, conductance-based computer model of intrinsic sensory neurons of the gastrointestinal tract. American Journal of Physiology-Gastrointestinal and Liver Physiology. 2014;307(5):G517–G532. 10.1152/ajpgi.00228.2013 25012843

[18] Luscher C, Streit J, Quadroni R, Luscher HR. Action potential propagation through embryonic dorsal root ganglion cells in culture. I. Influence of the cell morphology on propagation properties. Journal of neurophysiology. 1994;72(2):622–633. 10.1152/jn.1994.72.2.622 7983524

[19] Amir R, Devor M. Electrical excitability of the soma of sensory neurons is required for spike invasion of the soma, but not for through-conduction. Biophysical journal. 2003;84(4):2181–2191. 10.1016/S0006-3495(03)75024-3 12668427 PMC1302785

[20] Baker MD. Protein kinase C mediates up-regulation of tetrodotoxin-resistant, persistent Na^+^ current in rat and mouse sensory neurones. The Journal of physiology. 2005;567(3):851–867. 10.1113/jphysiol.2005.089771 16002450 PMC1474230

[21] Sheets PL, Jackson JO, Waxman SG, Dib-Hajj SD, Cummins TR. A Nav1. 7 channel mutation associated with hereditary erythromelalgia contributes to neuronal hyperexcitability and displays reduced lidocaine sensitivity. The Journal of physiology. 2007;581(3):1019–1031. 10.1113/jphysiol.2006.127027 17430993 PMC2170829

[22] Jeub M, Emrich M, Pradier B, Taha O, Gailus-Durner V, Fuchs H, et al The transcription factor Smad-interacting protein 1 controls pain sensitivity via modulation of DRG neuron excitability. PAIN^®^. 2011;152(10):2384–2398. 21862221 10.1016/j.pain.2011.07.006

[23] Tigerholm J, Petersson ME, Obreja O, Lampert A, Carr R, Schmelz M, et al Modeling activity-dependent changes of axonal spike conduction in primary afferent C-nociceptors. Journal of neurophysiology. 2014;111(9):1721–1735. 10.1152/jn.00777.2012 24371290 PMC4044369

[24] Soh H, Park CS. Inwardly rectifying current-voltage relationship of small-conductance Ca 2+-activated K+ channels rendered by intracellular divalent cation blockade. Biophysical Journal. 2001;80(5):2207–2215. 10.1016/S0006-3495(01)76193-0 11325723 PMC1301412

[25] Soh H, Park CS. Localization of divalent cation-binding site in the pore of a small conductance Ca 2+-activated K+ channel and its role in determining current-voltage relationship. Biophysical journal. 2002;83(5):2528–2538. 10.1016/S0006-3495(02)75264-8 12414687 PMC1302339

[26] Li W, Aldrich RW. Electrostatic influences of charged inner pore residues on the conductance and gating of small conductance Ca2+ activated K+ channels. Proceedings of the National Academy of Sciences. 2011;108(15):5946–5953. 10.1073/pnas.1103090108 PMC3076840 21422289

[27] Strøbæk D, Teuber L, Jørgensen TD, Ahring PK, Kjær K, Hansen RS, et al Activation of human IK and SK Ca 2+-activated K+ channels by NS309 (6, 7-dichloro-1H-indole-2, 3-dione 3-oxime). Biochimica et Biophysica Acta (BBA)-Biomembranes. 2004;1665(1):1–5. 15471565 10.1016/j.bbamem.2004.07.006

[28] Girault A, Haelters JP, Potier-Cartereau M, Chantôme A, Jaffrès PA, Bougnoux P, et al Targeting SKCa channels in cancer: potential new therapeutic approaches. Current medicinal chemistry. 2012;19(5):697–713. 10.2174/092986712798992039 22204342

[29] Steephen JE, Manchanda R. Differences in biophysical properties of nucleus accumbens medium spiny neurons emerging from inactivation of inward rectifying potassium currents. Journal of computational neuroscience. 2009;27(3):453 10.1007/s10827-009-0161-7 19488844

[30] Hougaard C, Fraser M, Chien C, Bookout A, Katofiasc M, Jensen B, et al A positive modulator of KCa2 and KCa3 channels, 4, 5-dichloro-1, 3-diethyl-1, 3-dihydro-benzoimidazol-2-one (NS4591), inhibits bladder afferent firing in vitro and bladder overactivity in vivo. Journal of Pharmacology and Experimental Therapeutics. 2009;328(1):28–39. 10.1124/jpet.108.143123 18820135

[31] Gover T, Moreira T, Weinreich D. Role of calcium in regulating primary sensory neuronal excitability In: Sensory Nerves. Springer; 2009 p. 563–587. 10.1007/978-3-540-79090-7_16 19655118

[32] Sah P, Louise Faber E. Channels underlying neuronal calcium-activated potassium currents. Progress in neurobiology. 2002;66(5):345–353. 10.1016/S0301-0082(02)00004-7 12015199

[33] Yoshimura N, Bennett NE, Hayashi Y, Ogawa T, Nishizawa O, Chancellor MB, et al Bladder overactivity and hyperexcitability of bladder afferent neurons after intrathecal delivery of nerve growth factor in rats. The Journal of neuroscience. 2006;26(42):10847–10855. 10.1523/JNEUROSCI.3023-06.2006 17050722 PMC6674760

[34] Takahashi R, Yoshizawa T, Yunoki T, Tyagi P, Naito S, de Groat WC, et al Hyperexcitability of bladder afferent neurons associated with reduction of Kv1. 4 *α*-subunit in rats with spinal cord injury. The Journal of urology. 2013;190(6):2296–2304. 10.1016/j.juro.2013.07.058 23896350 PMC3920734

[35] Yunoki T, Takimoto K, Kita K, Funahashi Y, Takahashi R, Matsuyoshi H, et al Differential contribution of Kv4-containing channels to A-type, voltage-gated potassium currents in somatic and visceral dorsal root ganglion neurons. Journal of neurophysiology. 2014;112(10):2492–2504. 10.1152/jn.00054.2014 25143545 PMC4233265

[36] Hayashi Y, Takimoto K, Chancellor MB, Erickson KA, Erickson VL, Kirimoto T, et al Bladder hyperactivity and increased excitability of bladder afferent neurons associated with reduced expression of Kv1. 4 *α*-subunit in rats with cystitis. American Journal of Physiology-Regulatory, Integrative and Comparative Physiology. 2009;296(5):R1661–R1670. 10.1152/ajpregu.91054.2008 19279288 PMC2689836

[37] Mandge D, Manchanda R. Computational studies on bladder small dorsal root ganglion neurons: Modelling BK channels. In: Engineering in Medicine and Biology Society (EMBC), 2015 37th Annual International Conference of the IEEE. IEEE; 2015. p. 5376–5379. 10.1109/EMBC.2015.7319606 26737506

[38] Mandge D, Bhatnagar A, Manchanda R. Computational model for intercellular communication between DRG neurons via satellite glial cells using ATP. In: Neural Engineering (NER), 2017 8th International IEEE/EMBS Conference on. IEEE; 2017. p. 648–651. 10.1109/EMBC.2019.8857153 31946364

[39] Aruljothi S, Mandge D, Manchanda R. A biophysical model of heat sensitivity in nociceptive C-fiber neurons. In: Neural Engineering (NER), 2017 8th International IEEE/EMBS Conference on. IEEE; 2017. p. 596–599.

[40] Hines ML, Carnevale NT. The NEURON simulation environment. NEURON. 2006;9(6). 10.1162/neco.1997.9.6.1179 9248061

[41] Malykhina A, Qin C, Greenwood-van Meerveld B, Foreman R, Lupu F, Akbarali H. Hyperexcitability of convergent colon and bladder dorsal root ganglion neurons after colonic inflammation: mechanism for pelvic organ cross-talk. Neurogastroenterology & Motility. 2006;18(10):936–948. 10.1111/j.1365-2982.2006.00807.x 16961697

[42] Kanda H, Clodfelder-Miller BJ, Gu JG, Ness TJ, DeBerry JJ. Electrophysiological properties of lumbosacral primary afferent neurons innervating urothelial and non-urothelial layers of mouse urinary bladder. Brain Research. 2016;1648:81–89. 10.1016/j.brainres.2016.06.042 27372884 PMC5027194

[43] Choi JS, Waxman SG. Physiological interactions between Nav 1.7 and Nav 1.8 sodium channels: a computer simulation study. Journal of neurophysiology. 2011;106(6):3173–3184. 10.1152/jn.00100.2011 21940606

[44] Benham C, Evans M, McBain C. Ca2+ efflux mechanisms following depolarization evoked calcium transients in cultured rat sensory neurones. The Journal of physiology. 1992;455(1):567–583. 10.1113/jphysiol.1992.sp019316 1484362 PMC1175659

[45] Thayer SA, Miller RJ. Regulation of the intracellular free calcium concentration in single rat dorsal root ganglion neurones in vitro. The Journal of Physiology. 1990;425(1):85–115. 10.1113/jphysiol.1990.sp018094 2213592 PMC1189839

[46] Deba F, Bessac BF. Anoctamin-1 Cl- channels in nociception: activation by an N-aroylaminothiazole and capsaicin and inhibition by T16A [inh]-A01. Molecular pain. 2015;11(1):55 10.1186/s12990-015-0061-y 26364309 PMC4567824

[47] La JH, Schwartz ES, Gebhart G. Differences in the expression of transient receptor potential channel V1, transient receptor potential channel A1 and mechanosensitive two pore-domain K+ channels between the lumbar splanchnic and pelvic nerve innervations of mouse urinary bladder and colon. Neuroscience. 2011;186:179–187. 10.1016/j.neuroscience.2011.04.049 21549810 PMC3118582

[48] Carnevale NT, Hines ML. The NEURON book. Cambridge University Press; 2006.

[49] Yoshimura N. Bladder afferent pathway and spinal cord injury: possible mechanisms inducing hyperreflexia of the urinary bladder. Progress in neurobiology. 1999;57(6):583–606. 10.1016/S0301-0082(98)00070-7 10221783

[50] Han C, Estacion M, Huang J, Vasylyev D, Zhao P, Dib-Hajj SD, et al Human Na V 1.8: enhanced persistent and ramp currents contribute to distinct firing properties of human DRG neurons. Journal of neurophysiology. 2015;113(9):3172–3185. 10.1152/jn.00113.2015 25787950 PMC4432682

[51] Black JA, Cummins TR, Yoshimura N, de Groat WC, Waxman SG. Tetrodotoxin-resistant sodium channels Nav1.8/SNS and Nav1.9/NaN in afferent neurons innervating urinary bladder in control and spinal cord injured rats. Brain research. 2003;963(1):132–138. 10.1016/S0006-8993(02)03957-4 12560118

[52] Shieh CC, Turner S, Zhang XF, Milicic I, Parihar A, Jinkerson T, et al A-272651, a nonpeptidic blocker of large-conductance Ca2+-activated K+ channels, modulates bladder smooth muscle contractility and neuronal action potentials. British journal of pharmacology. 2007;151(6):798–806. 10.1038/sj.bjp.0707278 17519951 PMC2014127

[53] Scholz A, Gruß M, Vogel W. Properties and functions of calcium-activated K+ channels in small neurones of rat dorsal root ganglion studied in a thin slice preparation. The Journal of Physiology. 1998;513(1):55–69. 10.1111/j.1469-7793.1998.055by.x 9782159 PMC2231273

[54] Zhang XL, Mok LP, Katz EJ, Gold MS. BK_Ca_ currents are enriched in a subpopulation of adult rat cutaneous nociceptive dorsal root ganglion neurons. European Journal of Neuroscience. 2010;31(3):450–462. 10.1111/j.1460-9568.2009.07060.x 20105244 PMC2843514

[55] Strøbæk D, Hougaard C, Johansen TH, Sørensen US, Nielsen EØ, Nielsen KS, et al Inhibitory gating modulation of small conductance Ca2+-activated K+ channels by the synthetic compound (R)-N-(benzimidazol-2-yl)-1, 2, 3, 4-tetrahydro-1-naphtylamine (NS8593) reduces afterhyperpolarizing current in hippocampal CA1 neurons. Molecular pharmacology. 2006;70(5):1771–1782. 10.1124/mol.106.027110 16926279

[56] Passmore GM, Selyanko AA, Mistry M, Al-Qatari M, Marsh SJ, Matthews EA, et al KCNQ/M currents in sensory neurons: significance for pain therapy. Journal of Neuroscience. 2003;23(18):7227–7236. 10.1523/JNEUROSCI.23-18-07227.2003 12904483 PMC6740665

[57] Passmore GM. Dorsal root ganglion neurones in culture: A model system for identifying novel analgesic targets? Journal of pharmacological and toxicological methods. 2005;51(3):201–208. 10.1016/j.vascn.2004.08.007 15862465

[58] Maingret F, Coste B, Padilla F, Clerc N, Crest M, Korogod SM, et al Inflammatory mediators increase Nav1. 9 current and excitability in nociceptors through a coincident detection mechanism. The Journal of general physiology. 2008;131(3):211–225. 10.1085/jgp.200709935 18270172 PMC2248717

[59] Bischoff U, Vogel W, Safronov BV. Na+-activated K+ channels in small dorsal root ganglion neurones of rat. The Journal of physiology. 1998;510(3):743–754. 10.1111/j.1469-7793.1998.743bj.x 9660890 PMC2231080

[60] Fox A, Nowycky M, Tsien R. Kinetic and pharmacological properties distinguishing three types of calcium currents in chick sensory neurones. The Journal of Physiology. 1987;394(1):149–172. 10.1113/jphysiol.1987.sp016865 2451016 PMC1191955

[61] Tong WC, Choi CY, Karche S, Holden AV, Zhang H, Taggart MJ. A computational model of the ionic currents, Ca2+ dynamics and action potentials underlying contraction of isolated uterine smooth muscle. PloS one. 2011;6(4):e18685 10.1371/journal.pone.0018685 21559514 PMC3084699

[62] Fox A, Nowycky M, Tsien R. Single-channel recordings of three types of calcium channels in chick sensory neurones. The Journal of physiology. 1987;394(1):173–200. 10.1113/jphysiol.1987.sp016865 2451017 PMC1191956

[63] Aosaki T, Kasai H. Characterization of two kinds of high-voltage-activated Ca-channel currents in chick sensory neurons. Pflügers Archiv. 1989;414(2):150–156. 2547195 10.1007/BF00580957

[64] Fukumoto N, Kitamura N, Niimi K, Takahashi E, Itakura C, Shibuya I. Ca 2+ channel currents in dorsal root ganglion neurons of P/Q-type voltage-gated Ca^2+^ channel mutant mouse, rolling mouse Nagoya. Neuroscience research. 2012;73(3):199–206. 10.1016/j.neures.2012.04.006 22575052

[65] Hilaire C, Diochot S, Desmadryl G, Richard S, Valmier J. Toxin-resistant calcium currents in embryonic mouse sensory neurons. Neuroscience. 1997;80(1):267–276. 10.1016/S0306-4522(97)00101-2 9252237

[66] Diochot S, Richard S, Valmier J. Diversity of voltage-gated calcium currents in large diameter embryonic mouse sensory neurons. Neuroscience. 1995;69(2):627–641. 10.1016/0306-4522(95)00267-M 8552255

[67] Li L, Bischofberger J, Jonas P. Differential gating and recruitment of P/Q-, N-, and R-type Ca^2+^ channels in hippocampal mossy fiber boutons. Journal of Neuroscience. 2007;27(49):13420–13429. 10.1523/JNEUROSCI.1709-07.2007 18057200 PMC6673086

[68] Matsuyoshi H, Masuda N, Chancellor MB, Erickson VL, Hirao Y, de Groat WC, et al Expression of hyperpolarization-activated cyclic nucleotide-gated cation channels in rat dorsal root ganglion neurons innervating urinary bladder. Brain research. 2006;1119(1):115–123. 10.1016/j.brainres.2006.08.052 16979600

[69] Kouranova E, Strassle B, Ring R, Bowlby M, Vasilyev D. Hyperpolarization-activated cyclic nucleotide-gated channel mRNA and protein expression in large versus small diameter dorsal root ganglion neurons: correlation with hyperpolarization-activated current gating. Neuroscience. 2008;153(4):1008–1019. 10.1016/j.neuroscience.2008.03.032 18450385

[70] Usachev YM, Thayer SA. Ca^2+^ influx in resting rat sensory neurones that regulates and is regulated by ryanodine-sensitive Ca^2+^ stores. The Journal of Physiology. 1999;519(1):115–130. 10.1111/j.1469-7793.1999.0115o.x 10432343 PMC2269497

[71] Luik RM, Wang B, Prakriya M, Wu MM, Lewis RS. Oligomerization of STIM1 couples ER calcium depletion to CRAC channel activation. Nature. 2008;454(7203):538 10.1038/nature07065 18596693 PMC2712442

[72] Jin X, Shah S, Liu Y, Zhang H, Lees M, Fu Z, et al Activation of the Cl^−^ channel ANO1 by localized calcium signals in nociceptive sensory neurons requires coupling with the IP3 receptor. Science signaling. 2013;6(290):ra73 10.1126/scisignal.2004184 23982204 PMC4135425

[73] Salzer I, Gantumur E, Yousuf A, Boehm S. Control of sensory neuron excitability by serotonin involves 5HT2C receptors and Ca^2+^-activated chloride channels. Neuropharmacology. 2016;110:277–286. 10.1016/j.neuropharm.2016.08.006 27511837 PMC6192515

[74] Xiao Q, Yu K, Perez-Cornejo P, Cui Y, Arreola J, Hartzell HC. Voltage-and calcium-dependent gating of TMEM16A/Ano1 chloride channels are physically coupled by the first intracellular loop. Proceedings of the National Academy of Sciences. 2011;108(21):8891–8896. 10.1073/pnas.1102147108 PMC3102354 21555582

[75] Hayashi T, Kondo T, Ishimatsu M, Yamada S, Nakamura Ki, Matsuoka K, et al Expression of the TRPM8-immunoreactivity in dorsal root ganglion neurons innervating the rat urinary bladder. Neuroscience research. 2009;65(3):245–251. 10.1016/j.neures.2009.07.005 19622375

[76] Olivares E, Salgado S, Maidana JP, Herrera G, Campos M, Madrid R, et al TRPM8-dependent dynamic response in a mathematical model of cold thermoreceptor. PloS one. 2015;10(10):e0139314 10.1371/journal.pone.0139314 26426259 PMC4591370

[77] Hamada K, Matsuura H, Sanada M, Toyoda F, Omatsu-Kanbe M, Kashiwagi A, et al Properties of the Na^+^/K^+^ pump current in small neurons from adult rat dorsal root ganglia. British journal of pharmacology. 2003;138(8):1517–1527. 10.1038/sj.bjp.0705170 12721107 PMC1573791

[78] Scheff N, Yilmaz E, Gold M. The properties, distribution and function of Na^+^–Ca^2+^ exchanger isoforms in rat cutaneous sensory neurons. The Journal of physiology. 2014;592(22):4969–4993. 10.1113/jphysiol.2014.278036 25239455 PMC4259538

[79] Courtemanche M, Ramirez RJ, Nattel S. Ionic mechanisms underlying human atrial action potential properties: insights from a mathematical model. American Journal of Physiology-Heart and Circulatory Physiology. 1998;275(1):H301–H321. 10.1152/ajpheart.1998.275.1.H301 9688927

[80] Spiess AN, Neumeyer N. An evaluation of R 2 as an inadequate measure for nonlinear models in pharmacological and biochemical research: a Monte Carlo approach. BMC pharmacology. 2010;10(1):6 10.1186/1471-2210-10-6 20529254 PMC2892436

[81] Frost J. Why Is There No R-Squared for Nonlinear Regression?; 2014. http://blog.minitab.com/blog/adventures-in-statistics-2/why-is-there-no-r-squared-for-nonlinear-regression.

[82] Catterall WA, Goldin AL, Waxman SG. International Union of Pharmacology. XLVII. Nomenclature and Structure-Function Relationships of Voltage-Gated Sodium Channels. Pharmacological Reviews. 2005;57(4):397–409. 10.1124/pr.57.4.4 16382098

[83] Willms AR, Baro DJ, Harris-Warrick RM, Guckenheimer J. An improved parameter estimation method for Hodgkin-Huxley models. Journal of computational neuroscience. 1999;6(2):145–168. 10.1023/A:1008880518515 10333160

[84] Sah P, Davies P. Calcium-activated potassium currents in mammalian neurons. Clinical and Experimental Pharmacology and Physiology. 2000;27(9):657–663. 10.1046/j.1440-1681.2000.03317.x 10972528

[85] Jancsó G, Maggi CA. Distribution of capsaicin-sensitive urinary bladder afferents in the rat spinal cord. Brain research. 1987;418(2):371–376. 10.1016/0006-8993(87)90106-5 2445416

[86] Keast J, de Groat WC. Segmental distribution and peptide content of primary afferent neurons innervating the urogenital organs and colon of male rats. Journal of Comparative Neurology. 1992;319(4):615–623. 10.1002/cne.903190411 1619047

[87] Bahia PK, Suzuki R, Benton DC, Jowett AJ, Chen MX, Trezise DJ, et al A functional role for small-conductance calcium-activated potassium channels in sensory pathways including nociceptive processes. Journal of Neuroscience. 2005;25(14):3489–3498. 10.1523/JNEUROSCI.0597-05.2005 15814779 PMC6725366

[88] Hougaard C, Eriksen B, Jørgensen S, Johansen T, Dyhring T, Madsen L, et al Selective positive modulation of the SK3 and SK2 subtypes of small conductance Ca2+-activated K+ channels. British journal of pharmacology. 2007;151(5):655–665. 10.1038/sj.bjp.0707281 17486140 PMC2014002

[89] Yoshimura N, Seki S, de Groat WC. Nitric oxide modulates Ca^2+^ channels in dorsal root ganglion neurons innervating rat urinary bladder. Journal of neurophysiology. 2001;86(1):304–311. 10.1152/jn.2001.86.1.304 11431511

[90] Yoshimura N, Seki S, Erickson KA, Erickson VL, Chancellor MB, de Groat WC. Histological and electrical properties of rat dorsal root ganglion neurons innervating the lower urinary tract. Journal of Neuroscience. 2003;23(10):4355–4361. 10.1523/JNEUROSCI.23-10-04355.2003 12764124 PMC6741085

[91] Dupont JL, Bossu JL, Feltz A. Effect of internal calcium concentration on calcium currents in rat sensory neurones. Pflügers Archiv European Journal of Physiology. 1986;406(4):433–435. 10.1007/BF00590950 2423955

[92] Yilmaz E, Watkins SC, Gold MS. Paclitaxel-induced increase in mitochondrial volume mediates dysregulation of intracellular Ca 2+ in putative nociceptive glabrous skin neurons from the rat. Cell calcium. 2017;62:16–28. 10.1016/j.ceca.2017.01.005 28109678 PMC5365154

[93] Verkhratsky A, Petersen OH. The endoplasmic reticulum as an integrating signalling organelle: from neuronal signalling to neuronal death. European journal of pharmacology. 2002;447(2):141–154. 10.1016/S0014-2999(02)01838-1 12151006

[94] Sterratt D, Graham B, Gillies A, Willshaw D. Principles of computational modelling in neuroscience. Cambridge University Press; 2011.

[95] Zeilhofer H, Swandulla D, Reeh P, Kress M. Ca2+ permeability of the sustained proton-induced cation current in adult rat dorsal root ganglion neurons. Journal of neurophysiology. 1996;76(5):2834–2840. 10.1152/jn.1996.76.5.2834 8930236

[96] De Schutter E. Modeling intracellular calcium dynamics In: De Schutter E, editor. Computational Modeling Methods for Neuroscientists. Cambridge, Massachusetts: The MIT Press; 2009 p. 93–105.

[97] McHugh J, Kenyon J. An Excel-based model of Ca2+ diffusion and fura 2 measurements in a spherical cell. American Journal of Physiology-Cell Physiology. 2004;286(2):C342–C348. 10.1152/ajpcell.00270.2003 14512292

[98] Fink CC, Slepchenko B, Moraru II, Watras J, Schaff JC, Loew LM. An image-based model of calcium waves in differentiated neuroblastoma cells. Biophysical Journal. 2000;79(1):163–183. 10.1016/S0006-3495(00)76281-3 10866945 PMC1300923

[99] Faville R, Pullan A, Sanders K, Smith N. A biophysically based mathematical model of unitary potential activity in interstitial cells of Cajal. Biophysical journal. 2008;95(1):88–104. 10.1529/biophysj.107.122507 18339738 PMC2426626

[100] Hille B, et al Ion channels of excitable membranes. vol. 507 Sinauer Sunderland, MA; 2001.

[101] Lu SG, Zhang X, Gold MS. Intracellular calcium regulation among subpopulations of rat dorsal root ganglion neurons. The Journal of physiology. 2006;577(1):169–190. 10.1113/jphysiol.2006.116418 16945973 PMC2000672

[102] Scheff NN, Lu SG, Gold MS. Contribution of endoplasmic reticulum Ca 2+ regulatory mechanisms to the inflammation-induced increase in the evoked Ca 2+ transient in rat cutaneous dorsal root ganglion neurons. Cell calcium. 2013;54(1):46–56. 10.1016/j.ceca.2013.04.002 23642703 PMC3671929

[103] Lokuta AJ, Komai H, McDowell TS, Valdivia HH. Functional properties of ryanodine receptors from rat dorsal root ganglia. FEBS letters. 2002;511(1-3):90–96. 10.1016/S0014-5793(01)03312-9 11821055

[104] Li YX, Rinzel J. Equations for InsP 3 receptor-mediated [Ca 2+] i oscillations derived from a detailed kinetic model: a Hodgkin-Huxley like formalism. Journal of theoretical Biology. 1994;166(4):461–473. 10.1006/jtbi.1994.1041 8176949

[105] De Schutter E, Smolen P. Calcium dynamics in large neuronal models. Methods in neuronal modeling: From ions to networks. 1998;2.

[106] Shutov LP, Kim MS, Houlihan PR, Medvedeva YV, Usachev YM. Mitochondria and plasma membrane Ca2+-ATPase control presynaptic Ca2+ clearance in capsaicin-sensitive rat sensory neurons. The Journal of physiology. 2013;591(10):2443–2462. 10.1113/jphysiol.2012.249219 23381900 PMC3678036

[107] Svichar N, Kostyuk P, Verkhratsky A. Mitochondria buffer Ca2+ entry but not intracellular Ca2+ release in mouse DRG neurones. Neuroreport. 1997;8(18):3929–3932. 10.1097/00001756-199712220-00017 9462468

[108] Boyman L, Williams GS, Khananshvili D, Sekler I, Lederer W. NCLX: the mitochondrial sodium calcium exchanger. Journal of molecular and cellular cardiology. 2013;59:205–213. 10.1016/j.yjmcc.2013.03.012 23538132 PMC3951392

[109] Wagner J, Keizer J. Effects of rapid buffers on Ca2+ diffusion and Ca2+ oscillations. Biophysical Journal. 1994;67(1):447–456. 10.1016/S0006-3495(94)80500-4 7919018 PMC1225377

[110] Helmchen F, Imoto K, Sakmann B. Ca2+ buffering and action potential-evoked Ca2+ signaling in dendrites of pyramidal neurons. Biophysical Journal. 1996;70(2):1069–1081. 10.1016/S0006-3495(96)79653-4 8789126 PMC1225009

[111] O’mullane LM, Keast JR, Osborne PB. Co-cultures provide a new tool to probe communication between adult sensory neurons and urothelium. The Journal of urology. 2013;190(2):737–745. 10.1016/j.juro.2013.01.048 23353045 PMC4630218

[112] Grynkiewicz G, Poenie M, Tsien RY. A new generation of Ca2+ indicators with greatly improved fluorescence properties. Journal of Biological Chemistry. 1985;260(6):3440–3450. 3838314

[113] Blatter L, Wier W. Intracellular diffusion, binding, and compartmentalization of the fluorescent calcium indicators indo-1 and fura-2. Biophysical journal. 1990;58(6):1491–1499. 10.1016/S0006-3495(90)82494-2 2275965 PMC1281101

[114] Nagai T, Sawano A, Park ES, Miyawaki A. Circularly permuted green fluorescent proteins engineered to sense Ca2+. Proceedings of the National Academy of Sciences. 2001;98(6):3197–3202. 10.1073/pnas.051636098 PMC30630 11248055

[115] Usachev YM, DeMarco SJ, Campbell C, Strehler EE, Thayer SA. Bradykinin and ATP accelerate Ca^2+^ efflux from rat sensory neurons via protein kinase C and the plasma membrane Ca 2+ pump isoform 4. Neuron. 2002;33(1):113–122. 10.1016/S0896-6273(01)00557-8 11779484

[116] Medvedeva YV, Kim MS, Usachev YM. Mechanisms of prolonged presynaptic Ca2+ signaling and glutamate release induced by TRPV1 activation in rat sensory neurons. Journal of Neuroscience. 2008;28(20):5295–5311. 10.1523/JNEUROSCI.4810-07.2008 18480286 PMC2694046

[117] Shmigol A, Verkhratsky A, Isenberg G. Calcium-induced calcium release in rat sensory neurons. The Journal of physiology. 1995;489(3):627–636. 10.1113/jphysiol.1995.sp021078 8788929 PMC1156834

[118] Lu SG, Gold MS. Inflammation-induced increase in evoked calcium transients in subpopulations of rat dorsal root ganglion neurons. Neuroscience. 2008;153(1):279–288. 10.1016/j.neuroscience.2008.02.006 18367340 PMC2396945

[119] Malykhina AP, Qin C, Foreman RD, Akbarali HI. Colonic inflammation increases Na+ currents in bladder sensory neurons. Neuroreport. 2004;15(17):2601–2605. 10.1097/00001756-200412030-00008 15570160

[120] Cordoba-Rodriguez R, Moore KA, Kao JP, Weinreich D. Calcium regulation of a slow post-spike hyperpolarization in vagal afferent neurons. Proceedings of the National Academy of Sciences. 1999;96(14):7650–7657. 10.1073/pnas.96.14.7650 PMC33596 10393875

[121] Faber EL, Sah P. Calcium-activated potassium channels: multiple contributions to neuronal function. The Neuroscientist. 2003;9(3):181–194. 10.1177/1073858403009003011 15065814

[122] Jenkins DP, Strøbæk D, Hougaard C, Jensen ML, Hummel R, Sørensen US, et al Negative gating modulation by (R)-N-(benzimidazol-2-yl)-1, 2, 3, 4-tetrahydro-1-naphthylamine (NS8593) depends on residues in the inner pore vestibule: pharmacological evidence of deep-pore gating of KCa2 channels. Molecular pharmacology. 2011;79(6):899–909. 10.1124/mol.110.069807 21363929 PMC3102549

[123] Mongan L, Hill M, Chen M, Tate S, Collins S, Buckby L, et al The distribution of small and intermediate conductance calcium-activated potassium channels in the rat sensory nervous system. Neuroscience. 2005;131(1):161–175. 10.1016/j.neuroscience.2004.09.062 15680700

[124] Fang X, McMullan S, Lawson SN, Djouhri L. Electrophysiological differences between nociceptive and non-nociceptive dorsal root ganglion neurones in the rat in vivo. The Journal of physiology. 2005;565(3):927–943. 10.1113/jphysiol.2005.086199 15831536 PMC1464557

[125] Vydyanathan A, Wu ZZ, Chen SR, Pan HL. A-type voltage-gated K+ currents influence firing properties of isolectin B 4-positive but not isolectin B 4-negative primary sensory neurons. Journal of neurophysiology. 2005;93(6):3401–3409. 10.1152/jn.01267.2004 15647393

[126] Nishiguchi J, Sasaki K, Seki S, Chancellor MB, Erickson KA, De Groat WC, et al Effects of isolectin B4-conjugated saporin, a targeting cytotoxin, on bladder overactivity induced by bladder irritation. European Journal of Neuroscience. 2004;20(2):474–482. 10.1111/j.1460-9568.2004.03508.x 15233756

[127] Zinck N, Downie J. IB4 afferent sprouting contributes to bladder dysfunction in spinal rats. Experimental neurology. 2008;213(2):293–302. 10.1016/j.expneurol.2008.06.006 18602393

[128] Fang X, Djouhri L, McMullan S, Berry C, Waxman SG, Okuse K, et al Intense isolectin-B4 binding in rat dorsal root ganglion neurons distinguishes C-fiber nociceptors with broad action potentials and high Nav1. 9 expression. Journal of Neuroscience. 2006;26(27):7281–7292. 10.1523/JNEUROSCI.1072-06.2006 16822986 PMC6673936

[129] Rasband MN, Park EW, Vanderah TW, Lai J, Porreca F, Trimmer JS. Distinct potassium channels on pain-sensing neurons. Proceedings of the National Academy of Sciences. 2001;98(23):13373–13378. 10.1073/pnas.231376298 PMC60878 11698689

[130] Brock MW, Mathes C, Gilly WF. Selective open-channel block of Shaker (Kv1) potassium channels by s-nitrosodithiothreitol (SNDTT). The Journal of general physiology. 2001;118(1):113–134. 10.1085/jgp.118.1.113 11429448 PMC2233744

[131] Bett GC, Dinga-Madou I, Zhou Q, Bondarenko VE, Rasmusson RL. A model of the interaction between N-type and C-type inactivation in Kv1. 4 channels. Biophysical journal. 2011;100(1):11–21. 10.1016/j.bpj.2010.11.011 21190652 PMC3010008

