# Supplementary figures and images for: A biophysically detailed computational model of urinary bladder small DRG neuron soma
Source: PLoS Comput Biol. 2018 Jul 18;14(7):e1006293. doi: 10.1371/journal.pcbi.1006293 (PMC6066259; doi:10.1371/journal.pcbi.1006293)

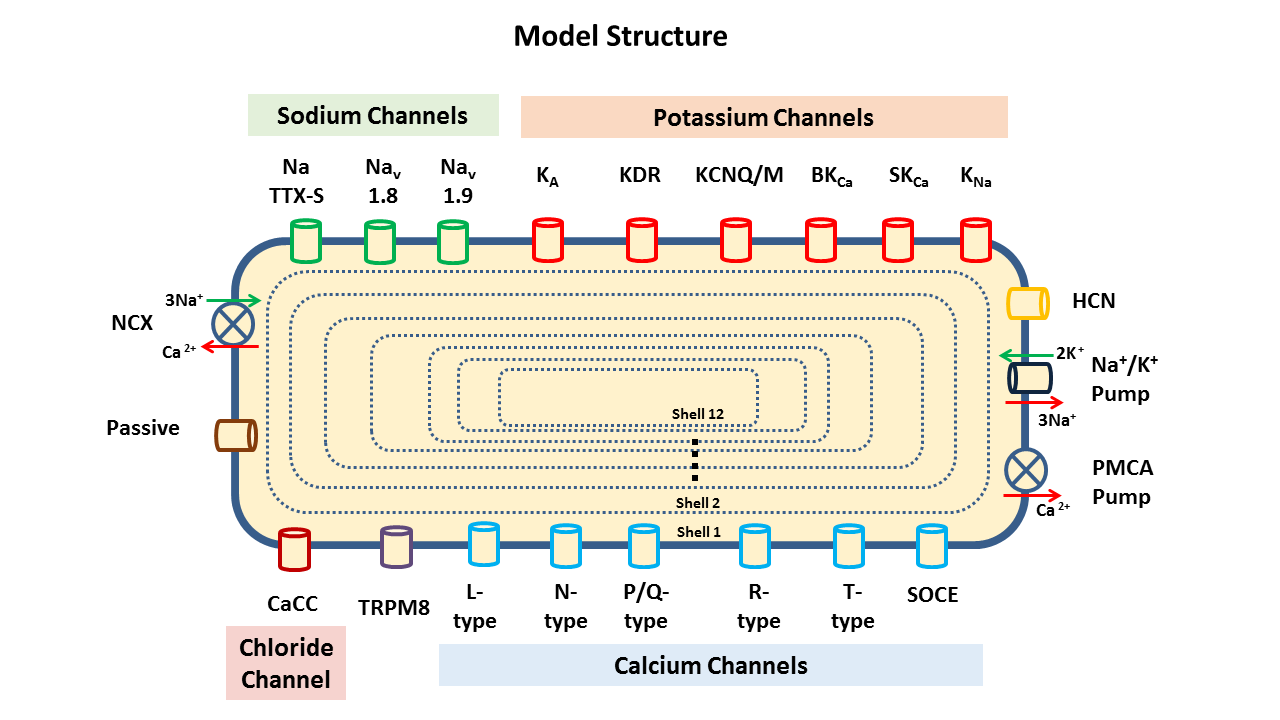

Supplement: S1 Fig — The model consists of 22 membrane mechanisms including Na+, K+, Ca2+, Cl-, and some non-specific ion channels such as TRPM8, HCN and passive channels as well as pumps such as Na+/K+-ATPase Pump, PMCA pump and Exchanger (NCX). The description of mechanisms is given in Methods and S2 Text. The soma is intracellularly divided into 12 concentric shells which facilitate diffusion of Ca2+ and IP3 (inositol 1,4,5 triphosphate) as well as in creating separate Ca2+ pools for different mechanisms. (TIF) [file pcbi.1006293.s001.tif]

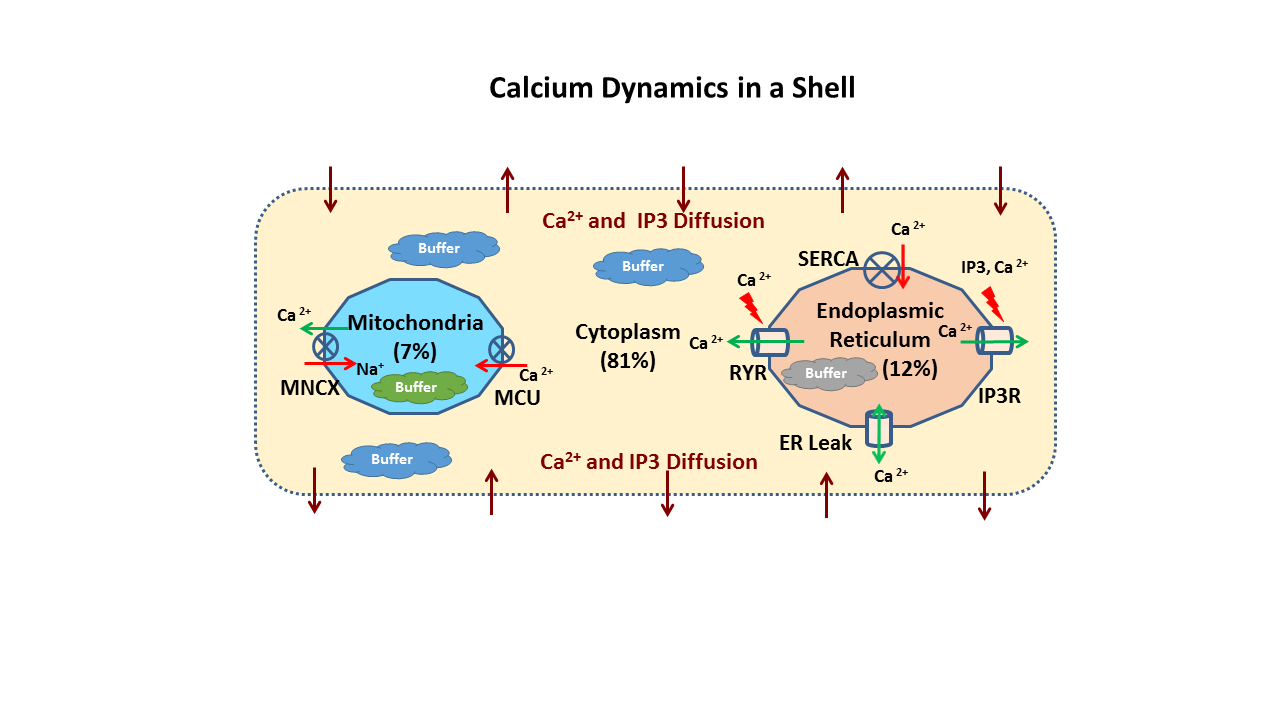

Supplement: S2 Fig — In each shell, 81% of the total volume is cytoplasm, 12% is occupied by endoplasmic reticulum (ER) and mitochondria make 7% of the total volume. Ca2+ and IP3 can diffuse from one shell to the another. Ca2+ coming in the cytoplasm to a shell via diffusion is buffered immediately. The ER has 4 mechanisms: SERCA is responsible for replenishing the ER Ca2+ concentration [Ca]ER caused by release from ryanodine receptors (RYR) which are activated by increase in cytoplasmic Ca2+; IP3R (IP3 receptors) which open on activation by IP3 molecules and Ca2+ ions; and the ER leak channel helps to maintain a steady resting state [Ca]ER. Ca2+ ions entering the ER as well as mitochondria is also buffered. Mitochondrial calcium entry occurs via mitochondrial uniporter (MCU) and calcium is released by mitochondrial sodium-calcium exchanger (MNCX). See Ca2+ dynamics in Methods. (TIF) [file pcbi.1006293.s002.tif]

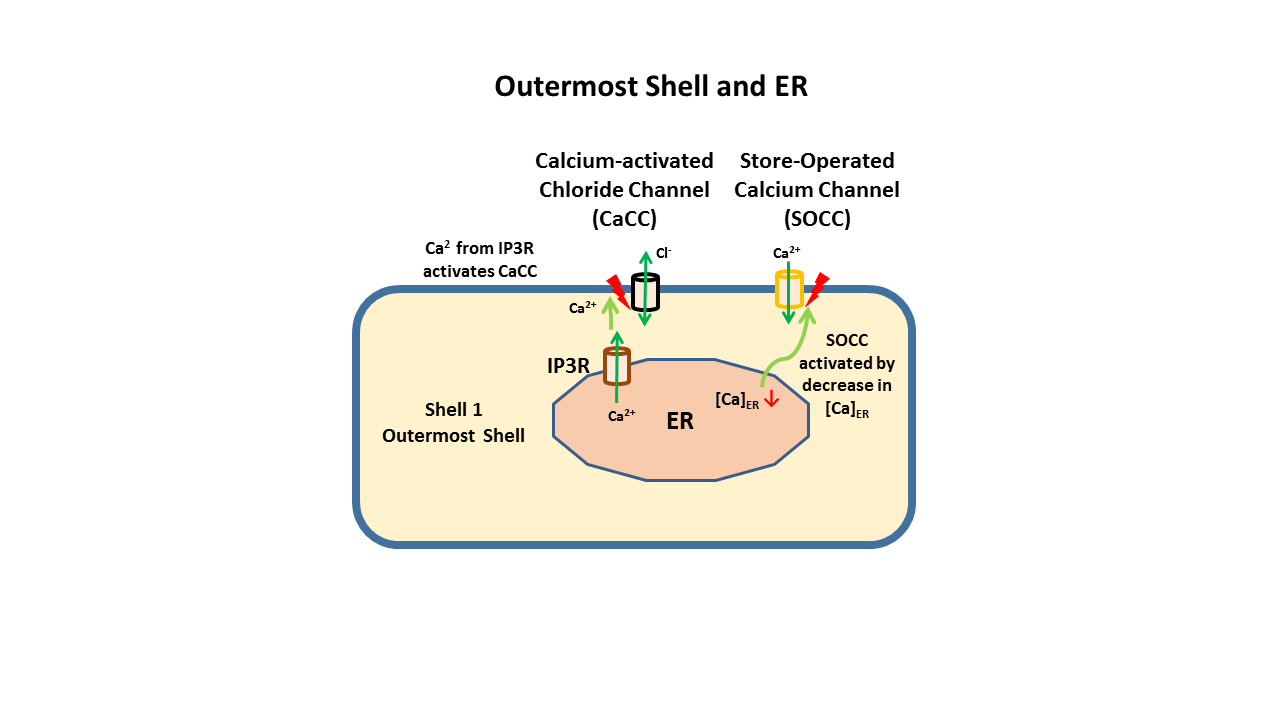

Supplement: S3 Fig — The Ca2+-activated Cl-s (CaCCs) are activated both by membrane potential and intracellular Ca2+. They are more potently gated by IP3R Ca2+ release in the outermost shell than by Ca2+ influx from voltage-gated Ca2+ channels on the membrane. The store-operated Ca2+ channels (SOCCs) are activated when there is a depletion of Ca2+ in the ER. The Orai1 and STIM1 proteins are responsible for store-operated Ca2+ entry in small DRG neurons. See S2 Text for more details. (TIF) [file pcbi.1006293.s003.tif]
