# Supplementary material for: A biophysically detailed computational model of urinary bladder small DRG neuron soma
Source: PLoS Comput Biol. 2018 Jul 18;14(7):e1006293. doi: 10.1371/journal.pcbi.1006293 (PMC6066259; doi:10.1371/journal.pcbi.1006293)
Supplement: S1 Text — (PDF) [file pcbi.1006293.s004.pdf]

Supporting Information: S1 Text.

A biophysically detailed computational model of urinary bladder small DRG neuron soma

Darshan Mandge, Rohit Manchanda

General description of the modelling methods

A general equation used in modelling channel currents is:

$$I_{Channel} = \bar{g} m^x h^y (V_m - E_{ion}) \tag{1}$$

where $I_{Channel}$ is the channel current, $\bar{g}$ is the maximum conductance offered by the channels, m (or n, o) and h are the activation and inactivation parameter of the ion channel current; x and y are their respective exponents representing the number of particles in the model. V_m is the membrane potential and E_{ion} is the reversal potential of the channel (if the channel is permeable to only one ion then it is the Nernst potential of the ion).

Voltage-gated calcium channels were modelled using GHK (Goldman-Hodgkin-Katz) current equation. The equation was used to model the current which uses permeability of the ions and takes into account the changes in extracellular and intracellular concentration:

$$I_{CaChannel} = p_{max} m h h_{ca} \frac{z^2 F^2 * V_m}{RT} \frac{[Ca]_i - [Ca]_o \exp\left(\dfrac{-zFV_m}{RT}\right)}{1 - \exp\left(\dfrac{-zFV_m}{RT}\right)} \tag{2}$$

where p_{max} is the maximum calcium permeability via the channels, h_{ca} is calcium-dependent inactivation (for L-type and N-type $\mathrm{Ca_v}$ channels only), $z = 2$, is the valence of Ca^{2+}, F is the Faraday's constant, V_m is the membrane potential, R is the universal gas constant, T is the temperature, $[Ca]_\mathrm{i}$ is the intracellular calcium concentration and $[Ca]_\mathrm{o}$ is the extracellular calcium concentration.

m and h can be function of $[Ca]_\mathrm{i}$, V_m and time, t. They are calculated using the differential equations:

$$\frac{dm}{dt} = \frac{m_\infty - m}{\tau_m}, \qquad \frac{dh}{dt} = \frac{h_\infty - h}{\tau_h} \tag{3}$$

Here, m_∞ and h_∞ are the steady state values, τ_m and τ_h are the time constants of activation and inactivation, respectively. m_∞ and h_∞ are calculated using either one of the sigmoidal equations:

1. Boltzmann equation:

$$m_\infty = \frac{1}{1 + exp\left(\frac{v_{half1} - V_m}{sf1}\right)}, \qquad h_\infty = \frac{1}{1 + exp\left(\frac{V_m - v_{half2}}{sf2}\right)} \tag{4}$$

where v_{half1} and v_{half2} are the half activation and inactivation parameters, respectively and sf_1 and sf_2 are the corresponding slope factors.

2. Using the forward (β) and reverse (α) rate constants for m (α_m & β_m) and h (α_h & β_h)

$$m_\infty = \frac{\alpha_m}{\alpha_m + \beta_m}, \qquad h_\infty = \frac{\alpha_h}{\alpha_h + \beta_h} \tag{5}$$

3. Hill equation:

$$m_\infty = \frac{[Ca]_i^n}{{K_d}^n + [Ca]_i^n} \tag{6}$$

where K_d is the dissociation constant, n is the Hill coefficient and $[Ca]_i$ is the intracellular calcium concentration. Calcium-dependent inactivation, h_{ca} in calcium current equation above is also coded using a Hill equation.

τ_m and τ_h are the activation and inactivation time constants, respectively. These are modelled using:

1. a Gaussian function

$$\tau_m = A_0 + A \exp\left(-\frac{(V_m - \mu)^2}{B}\right) \tag{7}$$

where μ is the mean of the Gaussian along the V_m axis, A_0, A & B are constants.

$$A = \frac{1}{(2\sigma\sqrt{(\pi)}}$$
$$B = 2\sigma^2$$

where σ is the standard deviation.

2. Using the forward (β) and reverse (α) rate constants for m α_m & β_m and h α_h & β_h.

$$\tau_m = \frac{\alpha}{\alpha + \beta} \tag{8}$$
