# Supplementary material for: A biophysically detailed computational model of urinary bladder small DRG neuron soma
Source: PLoS Comput Biol. 2018 Jul 18;14(7):e1006293. doi: 10.1371/journal.pcbi.1006293 (PMC6066259; doi:10.1371/journal.pcbi.1006293)
Supplement: S2 Text — (PDF) [file pcbi.1006293.s005.pdf]

Supporting Information: S2 Text.
A biophysically detailed computational model of urinary bladder small DRG neuron soma

Darshan Mandge, Rohit Manchanda

Additional membrane mechanisms in the model

TTX-Resistant Na_v1.9 channels. Na_v1.9 channels give rise to persistent inward currents in small DRG neurons. The window of overlap between the steady state activation and inactivation is shifted to more hyperpolarized potentials compared with TTX-S channels and Na_v1.8 channels (cf. Fig Aa in S2 Text with Fig 1A and 2A in Methods). Na_v1.9 channels start activating at potentials 10-15 mV negative to the resting membrane potential (RMP) of small DRG neurons, and hence, can regulate the RMP and boost subthreshold depolarizations [1,2]. Na_v1.9 expression is minimal in bladder small DRG neurons when compared to Na_v1.8 and TTX-S Na currents [3] and hence, its maximum conductance was kept to a small value in the model. The channel model was adapted from [4]. Comparisons of model and experimental data are shown in Fig A and the equations used for modelling are given below:

$$\alpha_m = \frac{1.548}{1+exp\left(\dfrac{V_m - 11.01}{-14.871}\right)} \qquad \beta_m = \frac{8.685}{1+exp\left(\dfrac{V_m + 112.4}{22.9}\right)} \qquad \alpha_h = \frac{0.2574}{1+exp\left(\dfrac{V_m + 63.264}{3.7193}\right)} \tag{1}$$

$$\beta_h = \frac{0.53984}{1+exp\left(\dfrac{V_m + 0.27853}{-9.0933}\right)} \qquad m_\infty = \frac{\alpha_m}{\alpha_m+\beta_m} \qquad h_\infty = \frac{\alpha_h}{\alpha_h+\beta_h} \qquad \tau_m = \frac{1}{\alpha_m+\beta_m} \qquad \tau_h = \frac{1}{\alpha_h+\beta_h} \tag{2}$$

$$\frac{dm}{dt} = \frac{m_\infty - m}{\tau_m} \qquad \frac{dh}{dt} = \frac{h_\infty - m}{\tau_h} \qquad I_{Na_v1.9} = \bar{g} * m * h(V_m - E_{Na}) \qquad \bar{g} = 0.00355\ \mathrm{S/cm^2} \quad E_{Na} = 40mV \tag{3}$$

KCNQ/M channels. KCNQ or K_v7 or K_M channels, are non-inactivating K^+ channels which underlie the K^+ M-currents ($I_{K(M)}$). These currents are inhibited by activation of muscarinic receptors and hence, the name M-current [5]. They can regulate the excitability of small DRG neurons being one of the major subthreshold current [6]. The presence of M-currents has been recently reported in bladder small DRG neurons [7]. Data for modelling was obtained from [5, 6, 8]. Figure B shows validation for the modelled channel. The KCNQ/M channel equations are given below:

$$n_\infty = \frac{1}{1+\exp\left(\frac{-30-V_m}{6}\right)} \qquad \alpha_n = 0.00395\exp\left(\frac{V_m+30}{40}\right) \qquad \beta_n = 0.00395\exp\left(-\frac{V_m+30}{20}\right) \qquad \tau_n = \frac{1}{\alpha_n+\beta_n} \tag{4}$$

$$\frac{dn}{dt} = \frac{n_\infty - n}{\tau_n} \qquad I_{KCNQ/M} = \bar{g} * n * (V_m - E_K) \qquad \bar{g} = 0.00071\ \mathrm{S/cm^2}, \qquad E_K = -84.7mV \tag{5}$$

Na^+-activated K^+ (K_{Na}) channels The K_{Na} channel are activated by intracellular Na^+ concentration ($[Na]_i$). These channels, which form the leakage currents, do not contribute to the action potentials but may be involved in defining the RMP of the small DRG neurons [9] and hence, were added in our soma model. The model was constructed based on the data by [9]. The $[Na]_i$ dependence of activation was modelled using Hill equation with EC_{50} of 38.7 mM

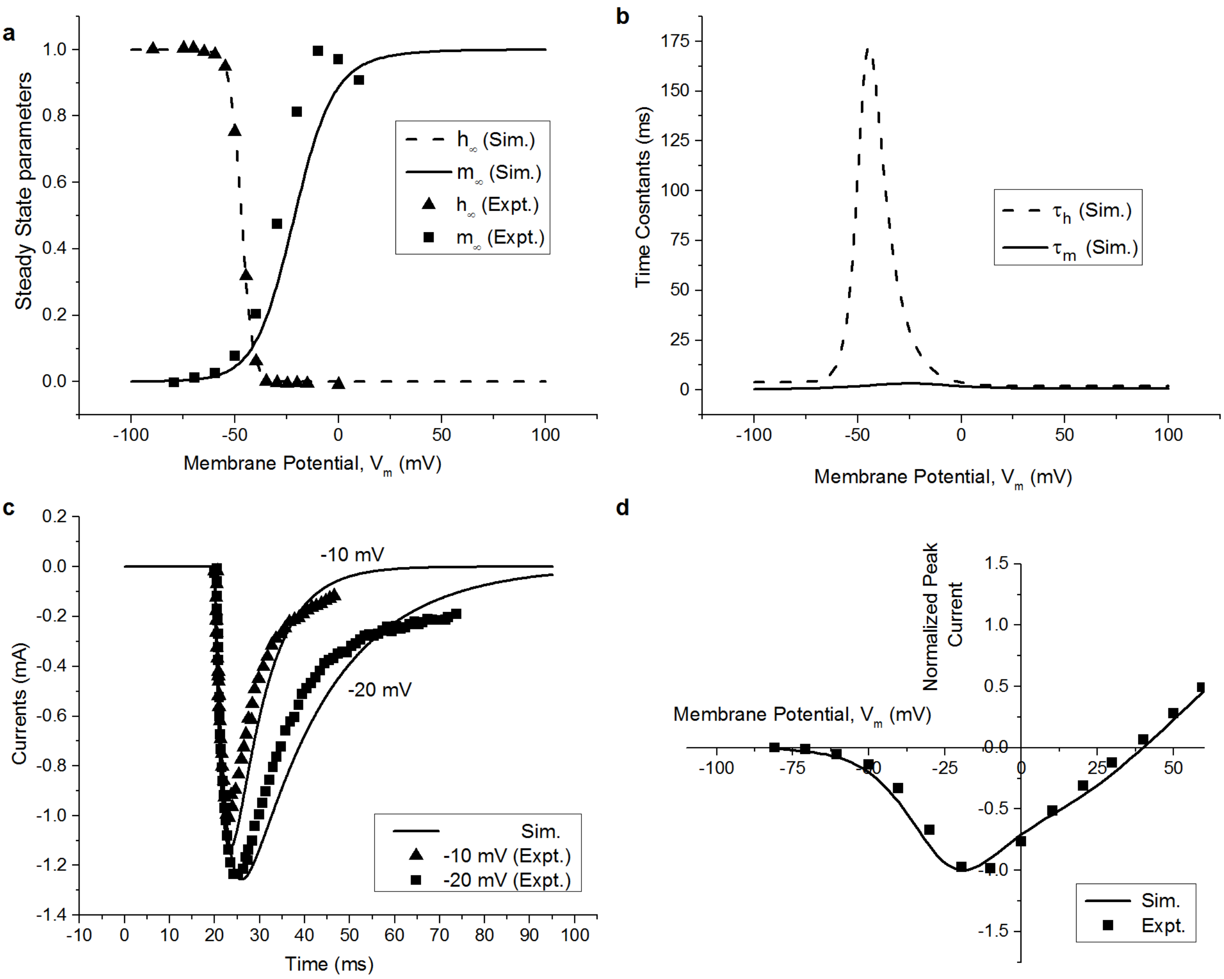

Fig A. Na_v1.9 channel. (a) Voltage dependence of steady state activation (m_∞, solid line) and inactivation (h_∞, dashed line) of the channel from the model (Sim.). Squares (m_∞) and triangles (h_∞) represent the experimental data (Expt., [4]). (b) The voltage dependence of time constants of activation (τ_m, dashed line) and inactivation (τ_h, solid line) for the model. (c) $I_{Nav\ 1.9}$ from the model using rectangular voltage clamp protocol. The holding potential was kept at -130 mV for 20 ms and the test potentials of 75 ms were applied to -20mV (squares) and -10 mV(triangles). The experimental data is from [4]. (d) The current-voltage (I-V) relationship generated from model by plotting the normalized peak current at each test potential. Protocol: Rectangular voltage clamp with a holding potential of -130 mV (20 ms) and test potentials from -80 to 60 mV. Other model parameters: $\bar{g} = 0.00355$ S/cm^2, $E_{Na} = 40$ mV, RMP $= -53.5$ mV, soma capacitance $= 28$ pF, soma diameter $= 24$ μm. The S values for model fits and their 5 % threshold values (given in brackets) are: $m_\infty = 0.153$ (0.05), $h_\infty = 0.008$ (0.05), I-V curve $= 0.065$ nA (0.085 nA), for voltage clamp currents at: -20 mV $= 0.112$ nA (0.061 nA) and -10 mV $= 0.485$ nA (0.056 nA).

and Hill coefficient was 3.5 as shown in Equation 6 and in Fig C.

$$w_\infty = \frac{1}{1 + \left(\frac{38.7}{[Na]_i}\right)^{3.5}} \quad \frac{dw}{dt} = \frac{w_\infty - w}{\tau_w} \quad \tau_w = 1 \qquad I_{K_{Na}} = \bar{g}w(V_m - E_K) \quad \bar{g} = 1.2 * 10^{-6}\ \text{S/cm}^2 \quad E_K = -84.7\ \text{mV} \tag{6}$$

P/Q-type Ca^{2+} (Ca_v 2.1) channels P/Q-type Ca^{2+} channels are formed by alternative splicing of the gene Ca_v 2.1. P/Q-type (and N-type) Ca^{2+} channels were found in the spinal terminals of DRG neurons and they play an important role in nociceptive signal transmission to the spinal dorsal horn neurons [10]. The equations used to model

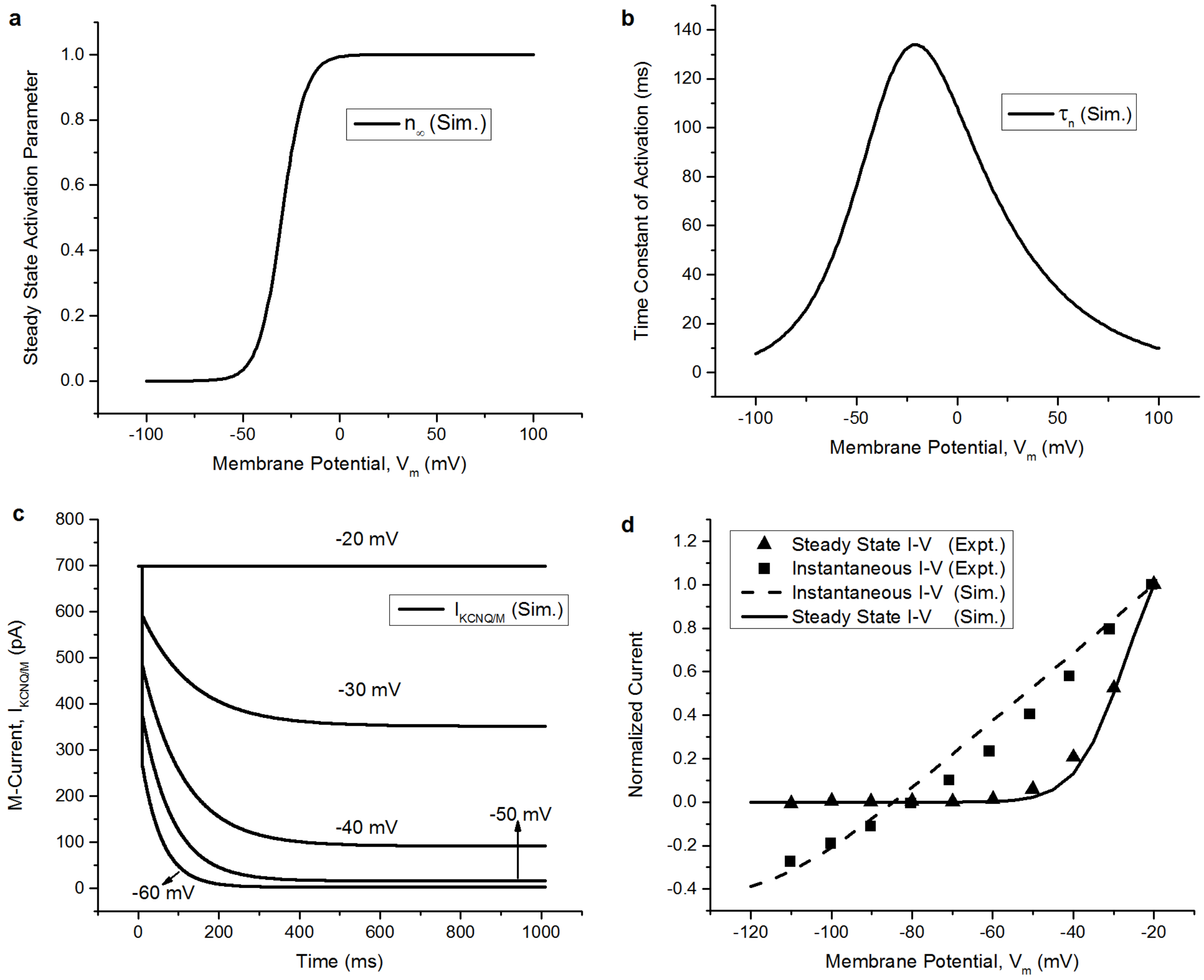

Fig B. KCNQ/M channel. (a) Voltage dependence of steady state of activation (n_∞, solid line) of the modelled channel (derived from [8]). (b) The activation time constant(τ_n) of the modelled channel. (c) $I_{K(M)}$ currents generated by the model using ractangular voltage clamp protocol. The holding potential was kept at −20 mV for 10 ms and test potentials to −20, −30, −40, −50 and −60 mV were applied for 1000 ms. (d) The steady state and instantaneous current-voltage (I-V) relationship generated from the model (solid line = steady state I-V, dashed line = instantaneous I-V curve) and experiments ([6], triangles = steady state I-V and squares = instantaneous I-V curve) The steady state I-V was plotted using the current values near the end of the clamp and the instantaneous I-V was plotted by measuring the instantaneous current at 1 ms from the start of the test clamp. Protocol: Voltage clamp steps were applied from −120 to −20 mV for 1000 ms from a holding potential of −20 mV. Other model parameters: $\bar{g} = 0.00071$ S/cm^2, $E_K = -84.7$ mV, RMP = −53.5 mV, soma capacitance = 28 pF, soma diameter = 24 μm. The S value for fits and their 5 % threshold values (given in brackets) are: steady state I-V curve = 0.03 nA (0.05 nA) and instantaneous I-V curve = 0.09 nA (0.064 nA).

the channels are given below:

$$m_\infty = \frac{1}{1 + \exp\left(\dfrac{-5.1 - V_m}{3.1}\right)} \qquad \tau_m = 0.35 + 5.51 \exp\left(-2\left(\frac{(V_m + 9.73)}{18.14}\right)^2\right) \qquad \frac{dm}{dt} = \frac{m_\infty - m}{\tau_m} \tag{7}$$

$$p_{max} = 0.008 \text{ cm/s} \qquad I_{P/Q-type} = p_{max} m \frac{z^2 F^2 V_m}{RT} \frac{[Ca]_i - [Ca]_o \exp\left(\frac{-zFV_m}{RT}\right)}{1 - \exp\left(\frac{-zFV_m}{RT}\right)} \tag{8}$$

The modelled channel is compared with experimental data in Fig D.

R-type Ca^{2+} channels. They are named so because at the time of their discovery, these channels were resistant to all the known blockers of Ca^{2+} channels. These are also called α1E or Ca_v2.3 channels. These channels are expressed in IB4-negative and TRPV1-positive DRG subgroup of neurons [11] which also include the bladder small DRG neurons.

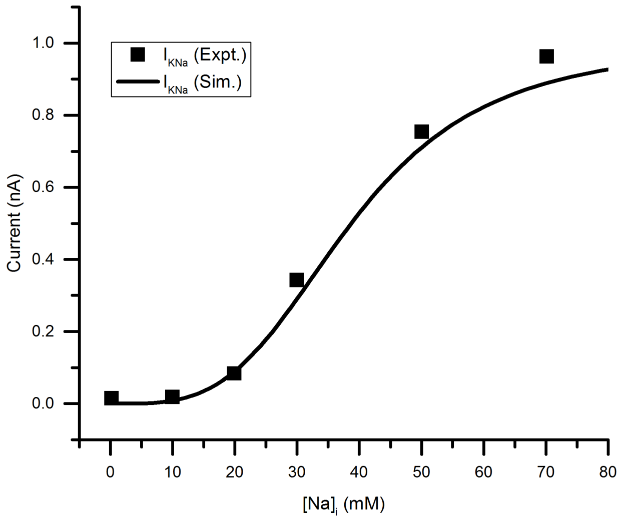

Fig C. Dependence of K_{Na} current on $[Na]_i$. Squares represent the experimental values from [9] and the solid line is from the model. $[Na]_i$ was changed from 0.1 to 80 mM. Other model parameters: $\bar{g}$ = 1*10^{-6} S/cm^2, $[K]_i$ = 85 nM, $[K]_o$= 5.6 mM, E_K = −77 mV, RMP = −53.5 mV, soma capacitance = 28 pF and soma diameter = 24 μm. The S value for K_{Na} current fit and the 5 % threshold value (given in brackets) are: 0.052 nA (0.05 nA).

Hilaire et al. [12] and Diochot et al. [13] studied the toxin resistant channels in embryonic mouse DRG neurons and found that their currents show a biexponential decay with a fast and a slow component as shown in (Fig Ed). To model the currents, two separate inactivation parameters (h_{fast} and h_{slow}) were used (as used in slow K_A channel model) to account for each component. The steady state inactivation curve for both the parameters was assumed to be identical. The parameters for modelling were adapted from [12–14]. The equations used for modelling these channel are given below:

$$m_\infty = \frac{1}{1+\exp\left(\frac{-5-V_m}{5}\right)} \qquad h_\infty = \frac{1}{1+\exp\left(\frac{V_m+51}{12}\right)} \qquad \frac{dm}{dt} = \frac{m_\infty - m}{\tau_m} \tag{9}$$

$$\frac{dh_{fast}}{dt} = \frac{h_\infty - h_{fast}}{\tau_{h_{fast}}} \qquad \frac{dh_{slow}}{dt} = \frac{h_\infty - h_{slow}}{\tau_{h_{slow}}} \qquad p_{max} = 0.0044 \text{ cm/s} \tag{10}$$

$$I_{R-type} = p_{max} m (0.4 * h_{fast} + 0.6 * h_{slow}) \frac{z^2F^2V_m}{RT} \frac{[Ca]_i - [Ca]_o \exp\left(\frac{-zFV_m}{RT}\right)}{1-\exp\left(\frac{-zFV_m}{RT}\right)} \tag{11}$$

The data points obtained from experiments for time constants (τ_m, τ_{hfast} and τ_{hslow}) were used and the FUNCTION_TABLE feature of the NEURON simulator which calculates τ values by linear interpolation.

T-type Ca^{2+} channels. T-type Ca^{2+} channels are low voltage activated (LVA) Ca^{2+} currents that start conducting above a membrane potential of −70 mV and inactivate completely on sustained depolarization [15]. T-type Ca^{2+} channels expression in bladder small DRG neurons is very low [16] compared to other HVA Ca^{2+} channels. Yoshimura et al., 2003 [16] observed very small whole-cell T-type calcium currents (< 0.2 nA) evolved by using a rectangular voltage clamp to −40 mV from −90 mV whereas the L-type and N-type HVA Ca^{2+} currents appear to contribute > 1 nA for a rectangular voltage clamp to 0 mV from −60 mV [17]. The following equations were used for modelling the T-type Ca^{2+} channel:

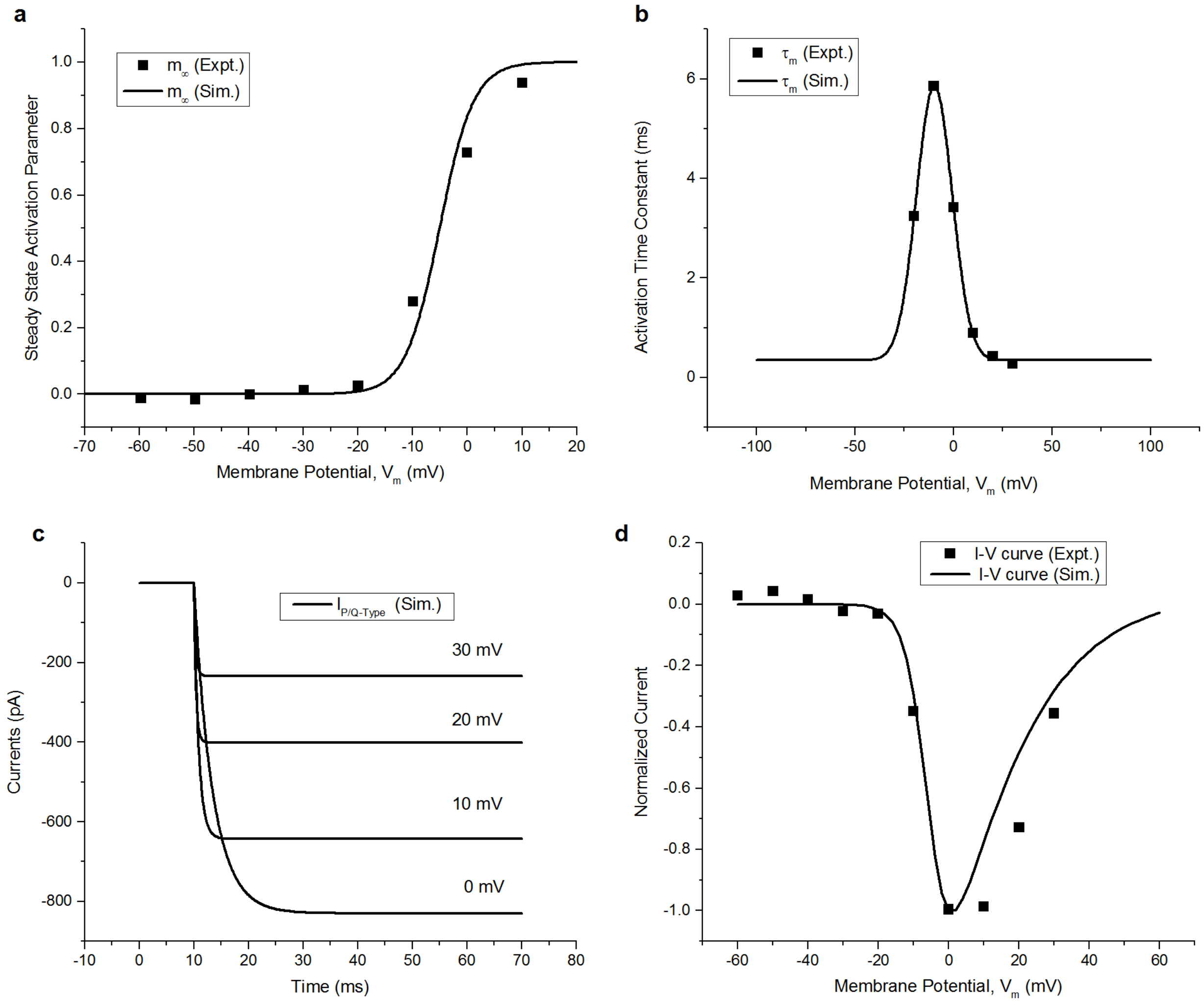

Fig D. P/Q-type Ca^{2+} channel. (a) Voltage dependence of steady state activation (m_∞) of the modelled channel (solid line, Sim. = Simulation) and from experiments (squares, Expt. = experiment data, [10]). (b) Time constants of activation (τ_m) from the model (solid line) and experiments (squares, [10]). (c) Rectangular voltage clamp currents obtained from the model (solid lines). Protocol: The holding potential (h.p.) was kept at −80 mV for 10 ms. The test potentials (t.p.'s), were applied from 0 to 30 mV in steps of 10 mV for 60 ms. (d) The normalized steady state current–voltage (I-V) relationship obtained from the model by using the same rectangular voltage clamp protocol as in (c) but the t.p.'s were applied from −60 to 60 mV. The magnitude of maximum inward current was recorded for t.p. ∼ 0 mV was used to normalize the curve. Other model parameters: p_{max} = 0.008 cm/s, $[\mathrm{Ca}]_\mathrm{i}$ = 1.36*10^{-4} mM, $[\mathrm{Ca}]_\mathrm{o}$ = 0.036 mM, E_{Ca} = 70 mV, RMP = −53.5 mV, soma capacitance = 28 pF and soma diameter = 24 μm. The S value of parameter fits and their 5 % threshold values (in brackets) are: m_∞ = 0.067 (0.05) and I-V curve = 0.114 (0.05).

$$m_\infty = \frac{1}{1+\exp\left(\dfrac{-55.29 - V_m}{6.38}\right)} \qquad h_\infty = \frac{1}{1+\exp\left(\dfrac{V_m + 76.59}{4.46}\right)} \qquad \frac{dm}{dt} = \frac{m_\infty - m}{\tau_m} \qquad \frac{dh}{dt} = \frac{h_\infty - h}{\tau_h} \tag{12}$$

$$I_{CaT-type} = p_{max} m h \frac{z^2 F^2 V_m}{RT} \frac{[Ca]_i - [Ca]_o \exp\left(\frac{-zFV_m}{RT}\right)}{1 - \exp\left(\frac{-zFV_m}{RT}\right)} \qquad p_{max} = 0.0125 \text{ cm/s} \tag{13}$$

The time constant of activation (τ_m, solid line) and inactivation (τ_h, dashed line) were obtained by interpolating data from [18] and [15] (Fig Fb, squares and triangles) as they could not be fit to a curve. The FUNCTION_TABLE feature of the NEURON was used which calculates the values of variables from a table of experimental values by linear interpolation.

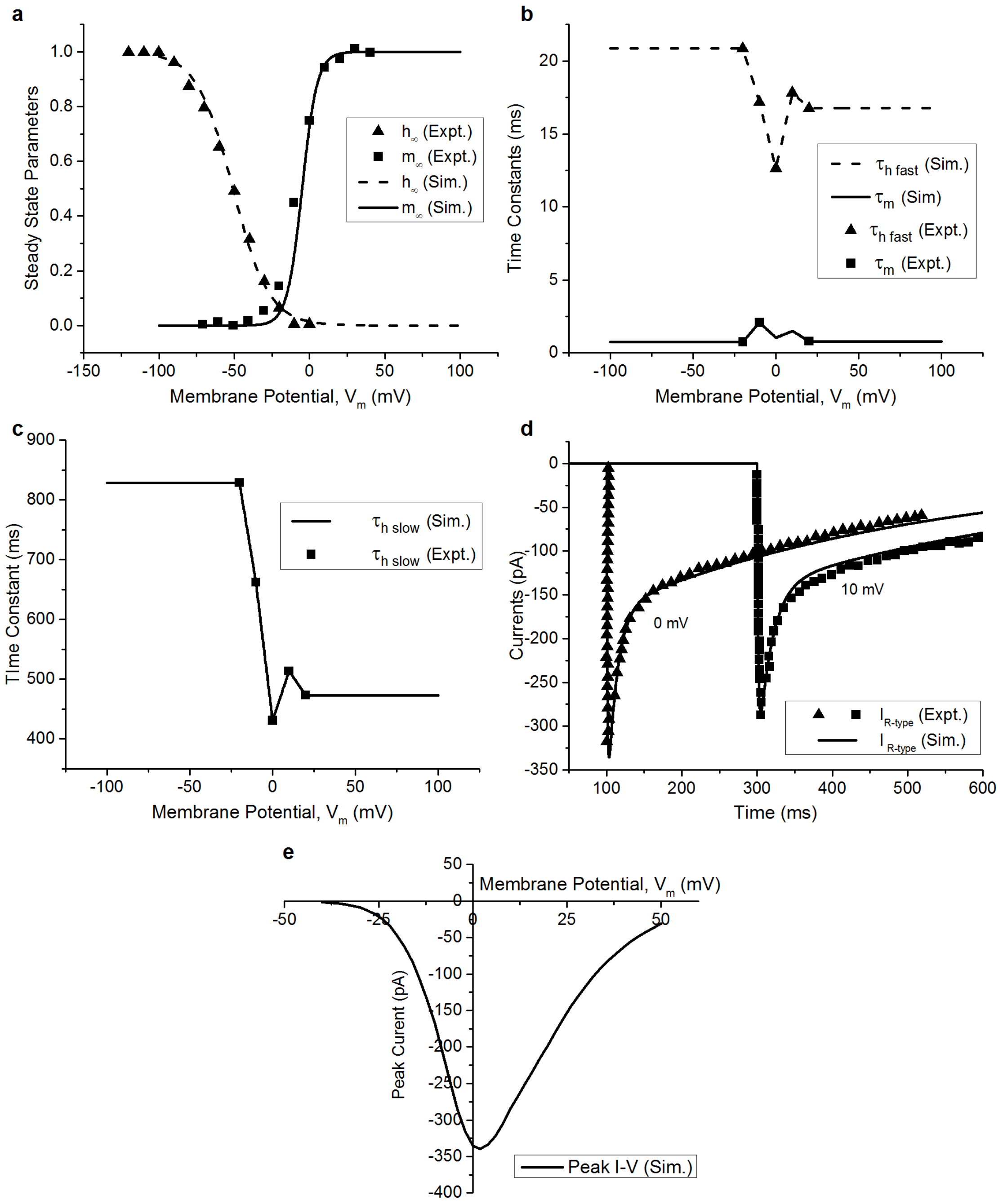

Fig E. R-type Ca^{2+} channel. (a) Voltage dependence of steady state activation (m_∞, solid line) and inactivation parameter (h_∞, dashed line) of the modelled channel (Sim. = Simulation). Squares (m_∞) and triangles (h_∞) represent the experimental data (Expt. = Experimental data) from [14] and [12], respectively. (b) Time constants of activation (τ_m, solid line) and fast inactivation ($\tau_{h_{fast}}$, dashed line) for simulated model. The corresponding experimental data (derived from [12]) are given by symbols (squares = τ_m, triangles = $\tau_{h_{fast}}$). (c) The slow inactivation time constant ($\tau_{h_{slow}}$) from the model (solid line) and experiments ([12], squares). (d) Rectangular voltage clamp currents obtained from the model (solid lines) and experiments (symbols). The triangles represent current from the test potential (t.p.) of 0 mV [12] and the squares represent those from 10 mV [13]. The solid lines are the corresponding simulation results. Protocol: The holding potential (h.p.) was kept at -100 mV for 100 ms (for the t.p. of 0 mV) and for 300 ms (for the t.p. of 10 mV). The t.p.'s, were maintained for 500 ms. (e) The peak current-voltage (I-V) relationship obtained from the model by rectangular voltage clamp protocol. The h.p. was kept at -100 mV for 100 ms and the test potentials from -40 to 50 mV, each of 500 ms duration were used. The peak inward current was recorded at each test potential. Other model parameters: $p_{max} = 0.0044$ cm/s, $[Ca]_i = 1.36*10^{-4}$ mM, $[Ca]_o = 0.037$ mM, E_{Ca} around 70 mV, RMP = -53.5 mV, soma capacitance = 28 pF and soma diameter = 24 μm. The S value for parameter fits and their 5 % threshold values (in brackets) are: $m_\infty = 0.073$ (0.05), $h_\infty = 0.023$ (0.05), currents, for voltage clamp currents: at 0 mV = 117.015 pA (15.616 pA) & at 10 mV = 57.11 pA (13.729 pA).

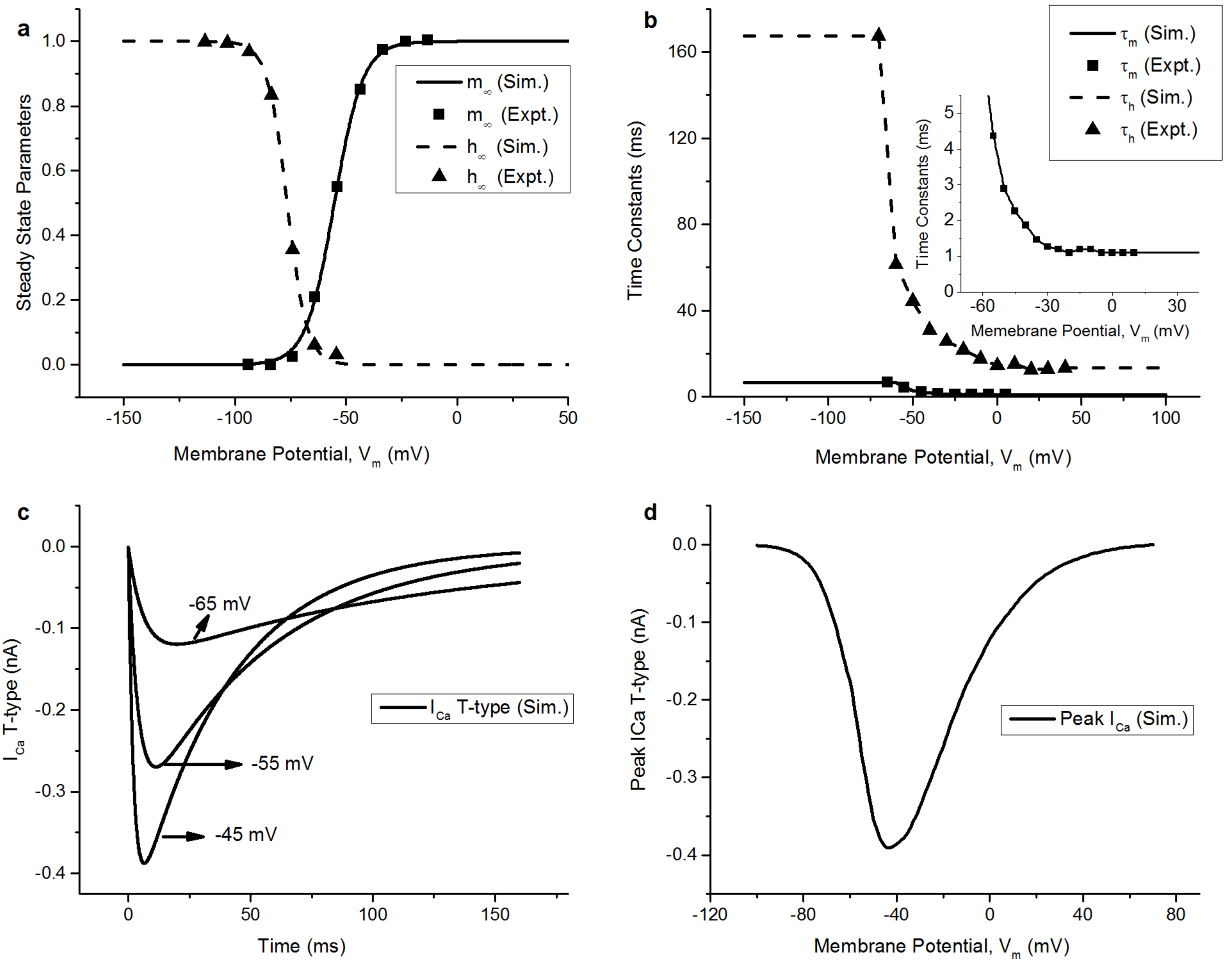

Fig F. T-type Ca^{2+} channel. (a) Voltage dependence of steady state activation (m_∞, solid line) and inactivation parameter (h_∞, dashed line) of the modelled channel (Sim. = Simulation). Squares (m_∞) and triangles (h_∞) represent the experimental data (Expt. = Experimental data) from [15]. (b) Time constants of activation (τ_m, solid line) and fast inactivation (τ_h, dashed line) for the simulated model. The corresponding experimental data (derived from [15, 18]) are given by symbols (squares = τ_m, triangles = τ_h). Inset in (b) shows τ_m on extended y (Time Constants) axis. (c) T-type currents obtained in response to rectangular voltage clamp from the model. The solid lines are the simulation results for indicated test potential. Protocol: Holding potential (h.p.) was -105 mV at the beginning of simulation. The testing potentials were maintained for 160 ms. (d) Peak I-V relationship obtained from the model by rectangular voltage clamp protocol. The h.p. was kept at -100 mV and the test potentials from -100 to 70 mV, each of 160 ms duration were used. The peak inward current was recorded at each test potential. Other model parameters: $p_{max} = 0.0125$ cm/s, $[Ca]_i = 1.36*10^{-4}$ mM, $[Ca]_o = 0.037$ mM, $E_{Ca} \sim 71$ mV, RMP $= -53.5$ mV, soma capacitance = 28 pF, soma diameter = 24 μm. The S values for model fits and their 5 % threshold values (given in brackets) are: $m_\infty = 0.002$ (0.5) and $h_\infty, = 1.3*10^{-5}$ (0.05).

Hyperpolarization-activated cyclic nucleotide-gated (HCN) channels. HCN channel are non-specific cation channel which activate at hyperpolarizing potentials compared to the RMP of DRG neurons (See Fig Ga). They play a role in controlling the frequency of action potentials and afterhyperpolarizations in bladder DRG neurons [19, 20]. Matsuyoshi et al. [19] reported the expression of HCN2 channels in more than 60% of bladder small afferent neurons. Kouronova et al. [21] also showed that HCN2 immunofluorescence is mostly found in small, IB4-negative DRG neurons. HCN current in bladder small DRG neurons have a slower time constant of activation and are less prominent than HCN in bladder medium-diameter DRG neurons [20]. The model was adapted from [21] which was reported for rat lumbar (L4-L6) small DRG neurons HCN channel as some bladder sensory neurons also originate from L6 DRG [22–24]. Fig G shows the comparison of HCN model with the experimental data.

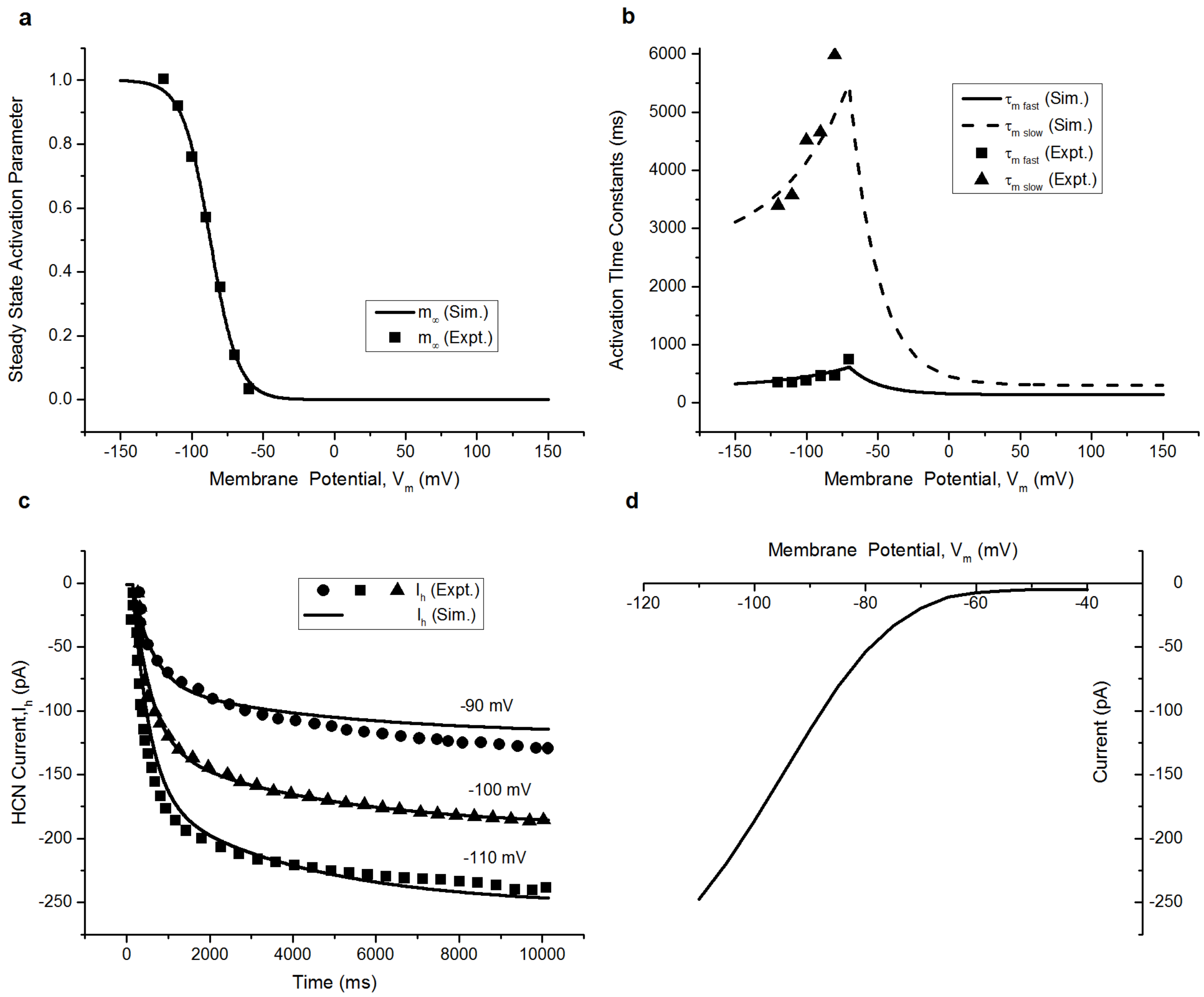

Fig G. HCN channel. (a) Voltage dependence of steady state of activation (m_∞) from the model (solid line) and experiments (squares, [21]. (b) The fast ($\tau_{m_{fast}}$) and slow ($\tau_{m_{slow}}$) time constants of activation. The $\tau_{m_{fast}}$ is shown by solid line (simulation =Sim.) and squares (experimental data = Expt.), and $\tau_{m_{slow}}$ is shown by dashed line (model) and triangles (experiments). (c) The currents generated by the model (solid lines) and the corresponding currents reported in experiments (symbols, [21]). The holding potential was −50 mv for 150 ms and the test potentials were applied to −110 mV (squares), −100 mV (triangles) and −90 mV (circles) for 10 s. The solid lines are the corresponding model traces and the corresponding numbers above the lines are clamp levels. (d) The current-voltage (I-V) curve generated by recording the currents at the end of 10 s voltage clamp test potentials applied from −110 to −40 mV and plotted against corresponding test potentials. Other model parameters: $\overline{g_{fast}} = 1.24 * 10^{-4}$ S/cm^2, $\overline{g_{slow}} = 6.76 * 10^{-5}$ S/cm^2, reversal potential for channel, E_h = −30 mV, RMP = −53.5 mV, soma capacitance = 28 pF, soma diameter = 24 μm. The S values of parameter fits and their 5 % threshold values are: m_∞ = 0.027 (0.05), for voltage clamp currents: at −110 mV = 14.411 pA (11.621 pA), at −100 mV = 8.672 pA (8.916 pA) and at −90 mV = 9.648 pA (6.103 pA).

$$m_\infty = \frac{1}{1 + exp\left(\frac{V_m + 87.2}{9.7}\right)} \qquad \frac{dm_{fast}}{dt} = \frac{m_\infty - m_{fast}}{\tau_{m_{fast}}} \qquad \frac{dm_{slow}}{dt} = \frac{m_\infty - m_{slow}}{\tau_{m_{slow}}} \tag{14}$$

$$\tau_{m_{fast}} = \begin{cases} 250 + 12\exp\left(\frac{V_m + 240}{50}\right) & \text{if } V_m < -70 \text{ mV} \\ 140 + 50\exp\left(\frac{V_m + 25}{-20}\right) & \text{if } V_m \geq -70 \text{ mV} \end{cases} \tag{15}$$

$$\tau_{m_{slow}} = \begin{cases} 2500 + 100\exp\left(\frac{V_m + 240}{50}\right) & \text{if } V_m < -70 \text{ mV} \\ 300 + 542\exp\left(\frac{V_m + 25}{-20}\right) & \text{if } V_m \geq -70 \text{ mV} \end{cases} \tag{16}$$

$$I_{HCN} = I_h = (\overline{g_{fast}} * m_{fast} + \overline{g_{slow}} * m_{slow})(V_m - E_h) \tag{17}$$

$$\overline{g_{fast}} = 1.24 * 10^{-4} \text{ S/cm}^2, \quad \overline{g_{slow}} = 6.76 * 10^{-5} \text{ S/cm}^2 \quad E_h = -30 \text{ mV} \tag{18}$$

Store-operated Ca^{2+} channels (SOCCs). Usachev and Thayer [25] showed the presence of a voltage-independent and intracellular Ca^{2+} store-regulated Ca^{2+} channels in the cell membrane of rat DRG neurons. This store-operated Ca^{2+} entry (SOCE) was also shown in injured and uninjured adult rat small DRG neurons [26]. The SOCE in small DRG neurons occurs via Orai1 and STIM1 proteins. The Orai1 forms the pore on the plasma membrane and STIM1 senses $[\mathrm{Ca}]_{\mathrm{ER}}$ [26]. SOCCs also known as Ca^{2+}-release-activated Ca^{2+} channels (CRACs) were modelled using the data from [27] for jurkat T cells. The activation parameter was made a function of ER Ca^{2+} concentration, $[\mathrm{Ca}]_{\mathrm{ER}}$ using a modified Hill equation with an EC_{50} of 0.069 mM. SOCCs generate a slow rising current (in seconds) for fast changes in $[\mathrm{Ca}]_{\mathrm{ER}}$ [27]. The equations used in the model are:

$$m_\infty = 1 - \frac{[Ca]_{ER}^{4.2}}{[Ca]_{ER}^{4.2} + EC_{50}^{4.2}} \qquad \tau_m = 5000 \qquad \frac{dm}{dt} = \frac{m_\infty - m}{\tau_m} \qquad p_{max} = 1.5 * 10^{-7}\ \mathrm{cm/s} \tag{19}$$

$$I_{SOCE} = p_{max} * m * \frac{z^2F^2V_m}{RT} \frac{[Ca]_i - [Ca]_o \exp\left(\frac{-zFV_m}{RT}\right)}{1 - \exp\left(\frac{-zFV_m}{RT}\right)} \tag{20}$$

Figure H depicts the relation between steady state activation parameter, m_∞ and $[\mathrm{Ca}]_{\mathrm{ER}}$.

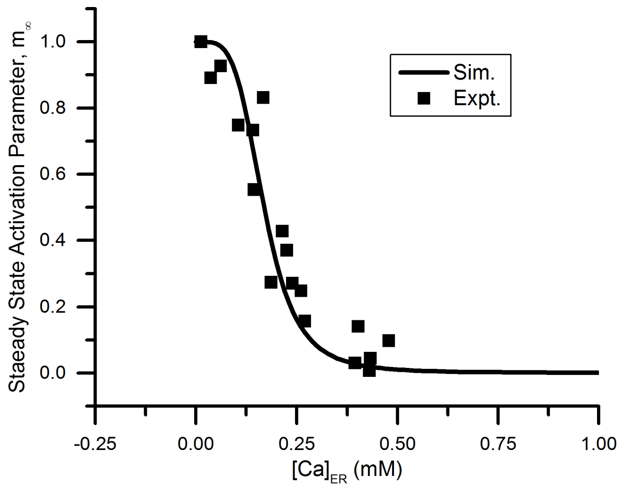

Fig H. Store-operated Ca^{2+} channel. $[\mathrm{Ca}]_{\mathrm{ER}}$ dependence of steady state activation (m_∞) from simulation (Solid line) and Squares (m_∞) represent the experimental data (Expt.) from [27]. Other parameters: $p_{max} = 1.5 * 10^{-7}$cm/s, $[\mathrm{Ca}]_\mathrm{i}$ = 1.36*10^{-4} mM, $[\mathrm{Ca}]_\mathrm{o}$ = 2 mM, E_{Ca} = 122 mV, RMP = −53.5 mV, soma capacitance = 28 pF and soma diameter = 24 μm. The S value of parameter fit and the 5 % threshold value (in brackets) is: m_∞ = 0.127 (0.05).

Ca^{2+}-activated Cl^- channels (CaCCs). Anoctamin 1 (ANO1) or TMEM16A CaCCs have been reported in small DRG neurons by some studies [28, 29]. They have been found to activate nociceptive firing while reducing the excitability of mechanoreceptive and proprioceptive channels [28]. They are activated by $[\mathrm{Ca}]_\mathrm{i}$ and V_m. CaCCs are coupled with IP3 receptors (IP3Rs) and are activated more potently by local Ca^{2+} release through IP3Rs ($[\mathrm{Ca}]_{\mathrm{IP3R}}$) than by the Ca^{2+} influx from voltage-gated Ca^{2+} channels [28]. Hence, the Ca^{2+} release from IP3Rs in outermost shell ($[\mathrm{Ca}]_{\mathrm{IP3R}}$) of the soma model was coupled to CaCC for activation. These were modelled using the data from [30] for HEK293 cells. Hill's equation was used for Ca^{2+} dependence of activation with the half-maximal effective concentration (EC_{50}) and Hill coefficient (HC) both as a function of V_m (Fig I).

$$n_\infty = \frac{1}{1 + \left(\frac{EC_{50}}{[Ca]_{IP3R}}\right)^{HC}} \qquad \frac{dn}{dt} = \frac{n_\infty - n}{\tau_n} \qquad \tau_n = 1 \tag{21}$$

$$HC = \begin{cases} -0.3126 \exp\left(\frac{-V_m}{81.02}\right) + 2.086 & \text{if } V_m \geq -100 \text{ mV} \\ 1.012 & \text{if } V_m < -100 \text{ mV} \end{cases} \tag{22}$$

$$EC_{50} = \begin{cases} 0.39175 \exp\left(\frac{-V_m}{38.307}\right) + 0.468 & \text{if } V_m \geq -100 \text{ mV} \\ 5.798 & \text{if } V_m < -100 \text{ mV} \end{cases} \tag{23}$$

$$I_{CaCC} = \bar{g} * n * (V_m - E_{Cl}) \qquad \bar{g} = 1 * 10^{-6} \text{ S/cm}^2 \qquad E_{Cl} = -32.7 \text{ mV} \tag{24}$$

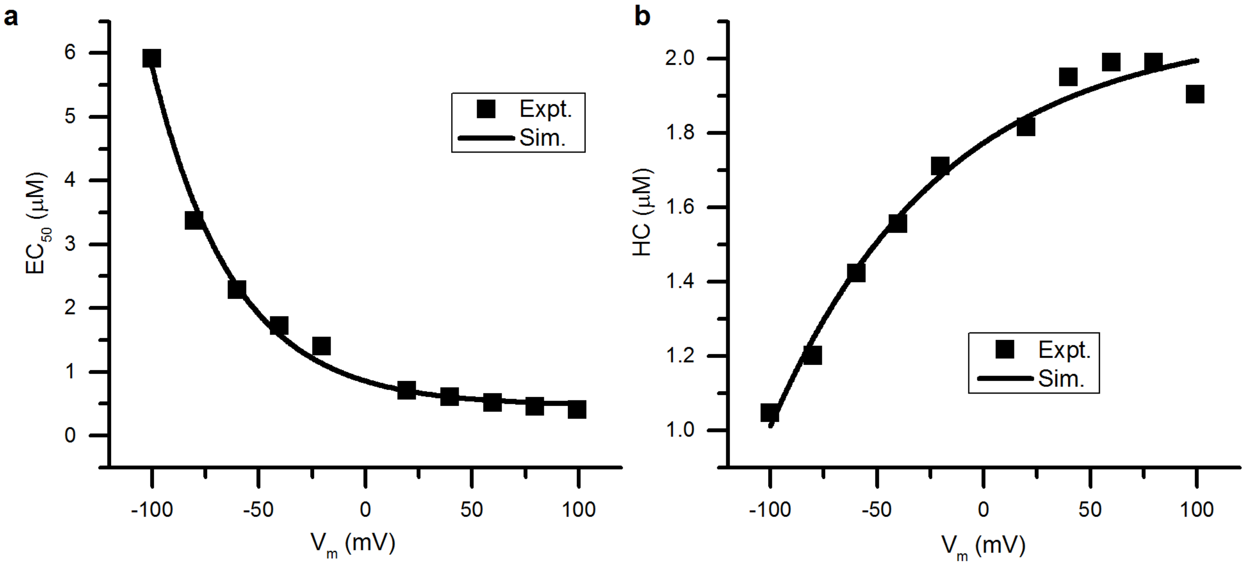

Fig I. Ca^{2+}-activated Cl^- channel. Half-maximal effective concentration, EC_{50} (a) and Hill Coefficient, HC (b) of steady state of activation (m_∞) plotted as a function of membrane potential, V_m. Solid line represent simulations data (Sim.) and squares represent corresponding experimental data (Expt.) from [30]. Other parameters: $\bar{g} = 1 * 10^{-6}$ S/cm^2, $[Cl]_i$ = 8 mM, $[Cl]_o$ = 140 mM, E_{Cl} = -32.7 mV, RMP = -53.5 mV, soma capacitance = 28 pF and soma diameter = 24 μm. The S value of parameter fits and their 5 % threshold values (in brackets) are: EC_{50} = 0.163 μM (0.276 μM) and HC = 0.053 μM (0.047μM).

Transient receptor potential cation channel subfamily M member 8 (TRPM8) The presence of TRPM8 channel has been reported in bladder small DRG neurons [24,31]. These channels are referred as cold receptors which are known to be active at room temperature (< 28°C) [31]. The model was adapted from [32].

$$a_{M8} = \frac{1}{1 + exp(-\frac{z_{M8}F}{RT}(V_m - v_h - \delta V))} \qquad v_h = \frac{CRT - \triangle E}{z_{M8}F} \qquad \frac{d(\delta V)}{dt} = \frac{\delta V_\infty - \delta V}{\tau_{\delta V}} \tag{25}$$

$$\delta V_\infty = \delta V_{min} + \frac{(\delta V_{max} - \delta V_{min})[Ca]_i}{[Ca]_i + K_{Ca}} \qquad \delta V_{min} = 0 \text{ mV} \qquad \delta V_{max} = 200 \text{ mV} \qquad K_{Ca} = 0.005 \text{ mM} \tag{26}$$

$$E_{M8} = 0 \text{ mV} \qquad \bar{g} = 1 * 10^{-7} \text{ S/cm}^2 \qquad pca = 0.01 \tag{27}$$

$$I_{TRPM8} = (1 - p_{ca}) * \bar{g} * a_{M8} * (V_m - E_{M8}) \qquad I_{Ca,TRPM8} = p_{ca} * \bar{g} * a_{M8} * (V_m - E_{M8}) \tag{28}$$

where a_{M8} is the activation parameter, z_{M8} is the temperature dependency factor, F is the Faraday's constant, R is the molar gas constant and T is the temperature (in K), v_h is the half activation voltage, C is the constant related to the pre-exponential factors of rate constants and $\triangle E$ is the difference between the activation energies for channel closing and opening and δV is the potential shift due to the $[\mathrm{Ca}]_\mathrm{i}$, E_{m8} is the reversal potential of TRPM8, $\bar{b}$ is the maximum conductance of TRPM8, $I_{Ca,TRPM8}$ is the fraction of TRPM8 current carried by Ca^{2+} ions and I_{TRPM8} is current carried by other ions.

Na^+/K^+-ATPase pump (Na^+/K^+ pump). Na^+/K^+ pumps maintain the homeostatic ionic balance across the membrane which is essential for keeping a steady RMP, osmotic balance and the excitability of the cells. Hamada et al. [33] have studied the Na^+/K^+ pump in lumbar small DRG neurons. They have shown Na^+/K^+ pump currents to be ouabain-sensitive, time-independent, voltage-dependent with slightly increasing currents for membrane potentials from −120 to 0 mV and generate a relatively constant current for positive potentials. The Na^+/K^+ pump model was adapted from [34]. The currents were modelled as sum of Hill equations with dependence on $[\mathrm{Na}]_\mathrm{i}$. The equations used for modelling are:

$$I_{Pump} = \bar{a}(C_m * 10^{-3})\left(\frac{i_{maxh}}{1+\left(\frac{6.7}{[Na]_i}\right)^3}\right) + (C_m * 10^{-3})\left(\frac{i_{maxl}}{1+\left(\frac{67.6}{[Na]_i}\right)^3}\right) \tag{29}$$

$$I_{Na,Pump} = 3I_{Pump} \qquad I_{K,Pump} = -2I_{Pump} \qquad \bar{a} = 0.001 \quad i_{maxh} = 1.62 \text{ pA/pF} \quad i_{maxl} = 0.99 \text{ pA/pF} \tag{30}$$

$\bar{a}$ is a constant that determines the amplitude of pump current, C_m ($\mu F/\mathrm{cm}^2$) is the specific membrane capacitance, i_{maxh} and i_{maxl} high- and low-affinity site maximum currents densities and I_{Pump} is the total pump current. $[Na]_i$ is in mM.

Na^+/Ca^{2+} Exchanger (NCX) The intracellular Ca^{2+} activation threshold of NCX is relatively high for sensory neurons [35]. NCX plays a major role Ca^{2+} extrusion in IB4-positive cutaneous DRG neurons with small diameters ($\leq 30\mu$m) but not in IB4-negative small DRG neurons [35, 36]. As most of the bladder small DRG neurons are IB4-negative ($> 75\%$) [24], we coded minimal effect of NCX on bladder small DRG neuron Ca^{2+} dynamics. The model for NCX was adapted from [37]:

$$k = 10^3 * R * T/F \qquad K_{qa} = \exp\left(\frac{0.35V_m}{k}\right) \qquad K_B = \exp\left(\frac{-0.65V_m}{k}\right) \qquad K_{Na} = 87.5 \text{ mM} \qquad K_{Ca} = 1.38 \text{ mM} \tag{31}$$

$$I_{maxNCX} = 1.1 * 10^{-5} \text{ mA/cm}^2 \qquad I_{NCX} = I_{maxNCX} * \frac{K_{qa} * [Na]_i^3 * [Ca]_o - K_B * [Na]_o^3 * [Ca]_i}{(K_{Na}^3 + [Na]_o^3) * (K_{Ca} + [Ca]_o) * (1 + 0.1K_B)} \tag{32}$$

$$I_{Na} = 3 * I_{NCX} \qquad I_{Ca} = -2 * I_{NCX} \tag{33}$$

where F is the Faraday's constant, R is the molar gas constant, T is the temperature (in K), k is a constant, K_{qa} and K_B are $\mathrm{V_m}$ dependent parameters, K_{Na} is the half-saturation constant for $[Na]_o$ and K_{Ca} is the half-saturation constant for $[Ca]_o$ and I_{maxNCX} is the maximum NCX current.

References

1. Herzog R, Cummins T, Waxman S. Persistent TTX-resistant Na^+ current affects resting potential and response to depolarization in simulated spinal sensory neurons. Journal of neurophysiology. 2001;86(3):1351–1364.

2. Dib-Hajj S, Black JA, Cummins TR, Waxman SG. NaN/Nav1.9: a sodium channel with unique properties. Trends in neurosciences. 2002;25(5):253–259.

3. Black JA, Cummins TR, Yoshimura N, de Groat WC, Waxman SG. Tetrodotoxin-resistant sodium channels Nav1.8/SNS and Nav1.9/NaN in afferent neurons innervating urinary bladder in control and spinal cord injured rats. Brain research. 2003;963(1):132–138.

4. Baker MD. Protein kinase C mediates up-regulation of tetrodotoxin-resistant, persistent Na^+ current in rat and mouse sensory neurones. The Journal of physiology. 2005;567(3):851–867.

5. Passmore GM. Dorsal root ganglion neurones in culture: A model system for identifying novel analgesic targets? Journal of pharmacological and toxicological methods. 2005;51(3):201–208.

6. Passmore GM, Selyanko AA, Mistry M, Al-Qatari M, Marsh SJ, Matthews EA, et al. KCNQ/M currents in sensory neurons: significance for pain therapy. Journal of Neuroscience. 2003;23(18):7227–7236.

7. Kanda H, Clodfelder-Miller BJ, Gu JG, Ness TJ, DeBerry JJ. Electrophysiological properties of lumbosacral primary afferent neurons innervating urothelial and non-urothelial layers of mouse urinary bladder. Brain Research. 2016;1648:81–89.

8. Maingret F, Coste B, Padilla F, Clerc N, Crest M, Korogod SM, et al. Inflammatory mediators increase Nav1. 9 current and excitability in nociceptors through a coincident detection mechanism. The Journal of general physiology. 2008;131(3):211–225.

9. Bischoff U, Vogel W, Safronov BV. Na^+-activated K^+ channels in small dorsal root ganglion neurones of rat. The Journal of physiology. 1998;510(3):743–754.

10. Fukumoto N, Kitamura N, Niimi K, Takahashi E, Itakura C, Shibuya I. Ca^{2+} channel currents in dorsal root ganglion neurons of P/Q-type voltage-gated Ca^{2+} channel mutant mouse, rolling mouse Nagoya. Neuroscience research. 2012;73(3):199–206.

11. Fang Z, Hwang JH, Kim JS, Jung SJ, Oh SB. R-type calcium channel isoform in rat dorsal root ganglion neurons. The Korean Journal of Physiology & Pharmacology. 2010;14(1):45–49.

12. Hilaire C, Diochot S, Desmadryl G, Richard S, Valmier J. Toxin-resistant calcium currents in embryonic mouse sensory neurons. Neuroscience. 1997;80(1):267–276.

13. Diochot S, Richard S, Valmier J. Diversity of voltage-gated calcium currents in large diameter embryonic mouse sensory neurons. Neuroscience. 1995;69(2):627–641.

14. Li L, Bischofberger J, Jonas P. Differential gating and recruitment of P/Q-, N-, and R-type Ca^{2+} channels in hippocampal mossy fiber boutons. Journal of Neuroscience. 2007;27(49):13420–13429.

15. Fox A, Nowycky M, Tsien R. Kinetic and pharmacological properties distinguishing three types of calcium currents in chick sensory neurones. The Journal of Physiology. 1987;394(1):149–172.

16. Yoshimura N, Seki S, Erickson KA, Erickson VL, Chancellor MB, de Groat WC. Histological and electrical properties of rat dorsal root ganglion neurons innervating the lower urinary tract. Journal of Neuroscience. 2003;23(10):4355–4361.

17. Yoshimura N, Seki S, de Groat WC. Nitric oxide modulates Ca^{2+} channels in dorsal root ganglion neurons innervating rat urinary bladder. Journal of neurophysiology. 2001;86(1):304–311.

18. Steephen JE, Manchanda R. Differences in biophysical properties of nucleus accumbens medium spiny neurons emerging from inactivation of inward rectifying potassium currents. Journal of computational neuroscience. 2009;27(3):453.

19. Matsuyoshi H, Masuda N, Chancellor MB, Erickson VL, Hirao Y, de Groat WC, et al. Expression of hyperpolarization-activated cyclic nucleotide-gated cation channels in rat dorsal root ganglion neurons innervating urinary bladder. Brain research. 2006;1119(1):115–123.

20. Masuda N, Hayashi Y, Matsuyoshi H, Chancellor MB, de Groat WC, Yoshimura N. Characterization of hyperpolarization-activated current (I_h) in dorsal root ganglion neurons innervating rat urinary bladder. Brain research. 2006;1096(1):40–52.

21. Kouranova E, Strassle B, Ring R, Bowlby M, Vasilyev D. Hyperpolarization-activated cyclic nucleotide-gated channel mRNA and protein expression in large versus small diameter dorsal root ganglion neurons: correlation with hyperpolarization-activated current gating. Neuroscience. 2008;153(4):1008–1019.

22. Jancsó G, Maggi CA. Distribution of capsaicin-sensitive urinary bladder afferents in the rat spinal cord. Brain research. 1987;418(2):371–376.

23. Keast J, De Groat W. Segmental distribution and peptide content of primary afferent neurons innervating the urogenital organs and colon of male rats. Journal of Comparative Neurology. 1992;319(4):615–623.

24. de Groat WC, Yoshimura N. Afferent nerve regulation of bladder function in health and disease. In: Sensory Nerves. Springer; 2009. p. 91–138.

25. Usachev YM, Thayer SA. Ca^{2+} influx in resting rat sensory neurones that regulates and is regulated by ryanodine-sensitive Ca^{2+} stores. The Journal of Physiology. 1999;519(1):115–130.

26. Gemes G, Bangaru MLY, Wu HE, Tang Q, Weihrauch D, Koopmeiners AS, et al. Store-operated Ca^{2+} entry in sensory neurons: functional role and the effect of painful nerve injury. Journal of Neuroscience. 2011;31(10):3536–3549.

27. Luik RM, Wang B, Prakriya M, Wu MM, Lewis RS. Oligomerization of STIM1 couples ER calcium depletion to CRAC channel activation. Nature. 2008;454(7203):538.

28. Jin X, Shah S, Liu Y, Zhang H, Lees M, Fu Z, et al. Activation of the Cl^- channel ANO1 by localized calcium signals in nociceptive sensory neurons requires coupling with the IP3 receptor. Science signaling. 2013;6(290):ra73.

29. Salzer I, Gantumur E, Yousuf A, Boehm S. Control of sensory neuron excitability by serotonin involves 5HT2C receptors and Ca^{2+}-activated chloride channels. Neuropharmacology. 2016;110:277–286.

30. Xiao Q, Yu K, Perez-Cornejo P, Cui Y, Arreola J, Hartzell HC. Voltage-and calcium-dependent gating of TMEM16A/Ano1 chloride channels are physically coupled by the first intracellular loop. Proceedings of the National Academy of Sciences. 2011;108(21):8891–8896.

31. Hayashi T, Kondo T, Ishimatsu M, Yamada S, Nakamura Ki, Matsuoka K, et al. Expression of the TRPM8-immunoreactivity in dorsal root ganglion neurons innervating the rat urinary bladder. Neuroscience research. 2009;65(3):245–251.

32. Olivares E, Salgado S, Maidana JP, Herrera G, Campos M, Madrid R, et al. TRPM8-dependent dynamic response in a mathematical model of cold thermoreceptor. PloS one. 2015;10(10):e0139314.

33. Hamada K, Matsuura H, Sanada M, Toyoda F, Omatsu-Kanbe M, Kashiwagi A, et al. Properties of the Na^+/K^+ pump current in small neurons from adult rat dorsal root ganglia. British journal of pharmacology. 2003;138(8):1517–1527.

34. Tigerholm J, Petersson ME, Obreja O, Lampert A, Carr R, Schmelz M, et al. Modeling activity-dependent changes of axonal spike conduction in primary afferent C-nociceptors. Journal of neurophysiology. 2014;111(9):1721–1735.

35. Scheff N, Yilmaz E, Gold M. The properties, distribution and function of Na^+–Ca^{2+} exchanger isoforms in rat cutaneous sensory neurons. The Journal of physiology. 2014;592(22):4969–4993.

36. Lu SG, Zhang X, Gold MS. Intracellular calcium regulation among subpopulations of rat dorsal root ganglion neurons. The Journal of physiology. 2006;577(1):169–190.

37. Courtemanche M, Ramirez RJ, Nattel S. Ionic mechanisms underlying human atrial action potential properties: insights from a mathematical model. American Journal of Physiology-Heart and Circulatory Physiology. 1998;275(1):H301–H321.
