# Supplementary material for: A biophysically detailed computational model of urinary bladder small DRG neuron soma
Source: PLoS Comput Biol. 2018 Jul 18;14(7):e1006293. doi: 10.1371/journal.pcbi.1006293 (PMC6066259; doi:10.1371/journal.pcbi.1006293)
Supplement: S1 Table — (PDF) [file pcbi.1006293.s006.pdf]

Supporting Information: S1 Table.
A biophysically detailed computational model of urinary bladder small DRG neuron soma

Darshan Mandge, Rohit Manchanda

Table for ionic mechanism parameters

Mechanism	Parameter	Description	Value	Units
Passive Channel	g	maximum conductance	0.0001	S/cm^2
Tetrodotoxin-Sensitive (TTX-S) Channel	$\bar{g}$	maximum conductance	0.0001	S/cm^2
Na_v1.8 Channel	$\bar{g}$	maximum conductance	0.0087177	S/cm^2
Na_v1.9 Channel	$\bar{g}$	maximum conductance	1*10^{-5}	S/cm^2
A-type K^+ (K_A) Channel	$\bar{g}$	maximum conductance	0.00136	S/cm^2
Delayed Rectifier (KDR) Channel	$\bar{g}$	maximum conductance	0.002688	S/cm^2
Large-Conductance Ca^{2+}-Activated K^+ Channels (BK_{Ca})	$\bar{g}$	maximum conductance	0.0009	S/cm^2
Small-Conductance Ca^{2+}-Activated K^+ Channels (SK_{Ca}): hSK3	$\bar{g}$	maximum conductance	0.0009	S/cm^2
KCNQ/M Channel	$\bar{g}$	maximum conductance	0.0001	S/cm^2
Na^+ activated (KNa) Channel	$\bar{g}$	maximum conductance	1*10^{-5}	S/cm^2
L-type Ca^{2+} Channel	p_{max}	maximum permeability	2.75*10^{-5}	cm/s
N-type Ca^{2+} Channel	p_{max}	maximum permeability	2.8*10^{-5}	cm/s
P/Q-type Ca^{2+} Channel	p_{max}	maximum permeability	8*10^{-6}	cm/s
R-type Ca^{2+} Channel	p_{max}	maximum permeability	1*10^{-8}	cm/s
T-type Ca^{2+} Channel	p_{max}	maximum permeability	1*10^{-8}	cm/s
Hyperpolarization-Activated Cyclic Nucleotide-Gated Channel	$\overline{g_{\text{fast}}}$<br>$\overline{g_{\text{slow}}}$	maximum conductance for m_{fast}<br>maximum conductance for m_{slow}	1.352*10^{-5}<br>6.7615*10^{-5}	S/cm^2<br>S/cm^2
Store-Operated Ca^{2+} Channel (SOCCs)	p_{max}	maximum permeability	1*10^{-9}	cm/s
Ca^{2+}-activated Cl^- Channel (CaCC)	$\bar{g}$	maximum conductance	1*10^{-6}	S/cm^2
Transient Receptor Potential Cation Channel Subfamily M Member 8	$\bar{g}$	maximum conductance	1*10^{-7}	S/cm^2
Na^+/K^+-ATPase Pump	$\bar{a}$	scaling factor	0.001	-
Na^+/Ca^{2+} Exchanger (NCX)	I_{max}	maximum current density	1.1*10^{-5}	mA/cm^2
Plasma Membrane Ca^{2+}-ATPase (PMCA) Pump	$pump0$	initial free pump density	4.232*10^{-13}	mol/cm^2
